# Dissemination of carbapenemase-producing *Enterobacterales* in Ireland from 2012 to 2017: a retrospective genomic surveillance study

**DOI:** 10.1099/mgen.0.000924

**Published:** 2023-03-14

**Authors:** Nazreen F. Hadjirin, Andries J. van Tonder, Beth Blane, John A. Lees, Narender Kumar, Niall Delappe, Wendy Brennan, Elaine McGrath, Julian Parkhill, Martin Cormican, Sharon J. Peacock, Catherine Ludden

**Affiliations:** ^1^​ Department of Medicine, University of Cambridge, Box 157, Addenbrooke’s Hospital, Hills Rd, Cambridge, CB2 0QQ, UK; ^2^​ Nuffield Department of Population Health, University of Oxford, Oxford OX3 7LF, UK; ^3^​ Department of Veterinary Medicine, University of Cambridge, Madingley Rd, Cambridge, CB3 0ES, UK; ^4^​ MRC Centre for Global Infectious Disease Analysis, School of Public Health, Imperial College London, London, UK; ^5^​ Wellcome Trust Sanger Institute Wellcome Trust Genome Campus, Hinxton, Cambridge, CB10 1SA, UK; ^6^​ National CPE Reference Laboratory, University Hospital Galway, Galway, Ireland; ^7^​ Antimicrobial Resistance and Microbial Ecology Group, School of Medicine, University of Galway, Galway, Ireland; ^8^​ Department of Infection Biology, Faculty of Infectious and Tropical Diseases, London School of Hygiene & Tropical Medicine, London WC1E 7HT, UK

**Keywords:** carbapenemase-producing *Enterobacterales *(CPE), hospital transmission, whole genome analysis, Ireland national survey, SNP-based phylogeny

## Abstract

The spread of carbapenemase-producing *Enterobacterales* (CPE) is of major public health concern. The transmission dynamics of CPE in hospitals, particularly at the national level, are not well understood. Here, we describe a retrospective nationwide genomic surveillance study of CPE in Ireland between 2012 and 2017. We sequenced 746 national surveillance CPE samples obtained between 2012 and 2017. After clustering the sequences, we used thresholds based on pairwise SNPs, and reported within–host diversity along with epidemiological data to infer recent putative transmissions. All clusters in circulating clones, derived from high-resolution phylogenies, of a species (* Klebsiella pneumoniae *, *Escherichia coli *, *Klebsiella oxytoca *, *Enterobacter cloacae *, *Enterobacter hormaechei* and *Citrobacter freundii *) were individually examined for evidence of transmission. Antimicrobial resistance trends over time were also assessed. We identified 352 putative transmission events in six species including widespread and frequent transmissions in three species. We detected putative outbreaks in 4/6 species with three hospitals experiencing prolonged outbreaks. The *bla*_OXA-48_ gene was the main cause of carbapenem resistance in Ireland in almost all species. An expansion in the number of sequence types carrying *bla*_OXA-48_ was an additional cause of the increasing prevalence of carbapenemase-producing *K. pneumoniae* and *E. coli *.

## Data Summary

The data supporting this work is available under NCBI bioprojects PRJEB19435, PRJEB22264, PRJEB19229, PRJEB24082, PRJEB24083, PRJEB24084, PRJEB24085, PRJEB24086, and PRJEB24087.

**Impact Statement**

Here we present the first nationwide report, to the best of our knowledge, that applies whole genome sequencing data in combination with epidemiological data to systematically infer and to improve our understanding of the dissemination dynamics of six carbapenemase-producing *Enterobacterales* (CPE) species (*n*=746 isolates) in Ireland between 2012 and 2017. We identified molecular evidence that supports widespread and frequent transmission among *K. pneumoniae *, *K. oxytoca* and *E. hormaechei,* with some hospitals experiencing prolonged outbreaks involving complex transmission dynamics. We clarify the role of the *bla*_OXA-48_ gene as the main cause of carbapenem resistance in Ireland in almost all species. Whole genome sequencing integrated with epidemiological data can contribute to healthcare-associated infection investigations by inferring evidence for or against transmission events as well as helping to characterize CPE clones circulating within the clinical setting. Transmission dynamics of CPE in hospitals can help improve targeted infection control and public health interventions in the future.

## Introduction

The spread of carbapenemase-producing *Enterobacterales* (CPE) is of major public health concern [1, 2] with multi-drug resistance being reported worldwide [3–5]. The European survey of CPE (EuSCAPE) from November 2013 to May 2014 was the first comprehensive surveillance study to provide a systematic overview of the occurrence and epidemiology of carbapenem-resistant *Enterobacterales* in the clinical setting and showed that while prevalence varied between countries, *K. pneumoniae* (37%) and *E. coli* (19%) were the major carbapenemase producers [3].

Carbapenemases are enzymes which hydrolyse carbapenem antibiotics with a broad spectrum of activity, conferring resistance, and are commonly produced by a range of *Enterobacterales* species. The most common carbapenemases acquired by *Enterobacterales* include *K. pneumoniae* carbapenemase (KPC), Verona integron-mediated metallo-β-lactamase (VIM), New Delhi metallo-beta-lactamase (NDM) and OXA-48-like, although considerable variation in their epidemiology exists across the world [3, 6, 7]. Acquisition frequently occurs via transfer on mobile genetic elements (MGEs), such as plasmids, and therefore transmissions both within and between species have occurred [6–10]. The spread of carbapenemases within *Enterobacterales* may also be driven by clonal expansion of successful CPE sequence types (STs) that stably maintain carbapenemase genes, for example ST258/ST512 in *K. pneumoniae* and ST131 in *E. coli* [5]. Other high-risk multi-drug-resistant and virulent STs of *K. pneumoniae* include ST11, ST15, ST37 and ST147 [11, 12].

Multiple genomic studies have documented the potential of carbapenemase-positive *K. pneumoniae* to spread in hospital environments, while more recently, several have explored nosocomial transmissions in other CPE in hospital environments [9–11, 13–17]. The transmission dynamics of CPE in the clinical setting can be further complicated due to diverse dissemination mechanisms (e.g. plasmid spread between single or multiple species, dissemination of multiple STs, diversity in reservoirs) that could contribute to a rapid increase of resistance [8, 18, 19]. Therefore, a comprehensive understanding of the nature and frequency of CPE nosocomial transmission is needed to help develop strategies for intervention to reduce the burden of CPE infections.

CPE was first reported in Ireland in 2009 and was subsequently detected in multiple hospitals and associated with outbreaks. Growing concern regarding the expansion of CPE in Ireland led to the declaration of a public health emergency by the Minister of Health in October 2017 followed by a series of measures to manage this phenomenon. Here, we describe a retrospective nationwide genomic surveillance study of CPE in Ireland between 2012 and 2017 to improve understanding of the CPE transmission dynamics, the extent of within- and between–hospital transmissions, as well as resistance trends during this time period.

## Methods

### Study isolates and setting

A voluntary enhanced surveillance system for all CPE isolates has been in place in Ireland since 2011, which was made mandatory in January 2017. We sequenced a total of 840 CPE that were submitted to the National CPE Reference Laboratory, Galway University Hospital, Ireland. We defined the study period as from April 2012 to June 2017 since collection of isolates ceased at the end of July 2017 because resources to extend the project were not available. Accordingly we sequenced the samples collected from 33 hospitals in Ireland during this period. One isolate per patient of any *Enterobacterales* species confirmed as a carbapenemase-producer from any specimen type was included. Out of our 840 initial samples, 94 were excluded from the analysis based on reasons such as lack of growth, the low quality of sequence data or species/carbapenem species misidentification. Full details of the sample selection is given in the Supplementary text. The remaining 746 samples were included in our analysis.

Among the 746 samples, 735 were from patient body sites (491 colonizing, 233 invasive and 11 unknown sites) and 11 from the environment that was associated with a patient. Colonizing samples were detected on programmes of surveillance culture of rectal swabs or faeces from people admitted to healthcare facilities and identified as at high risk of colonization based on national guidance. Invasive isolates were detected from diagnostic samples submitted in the course of investigation of clinical evidence of infection.

**Fig. 1.**
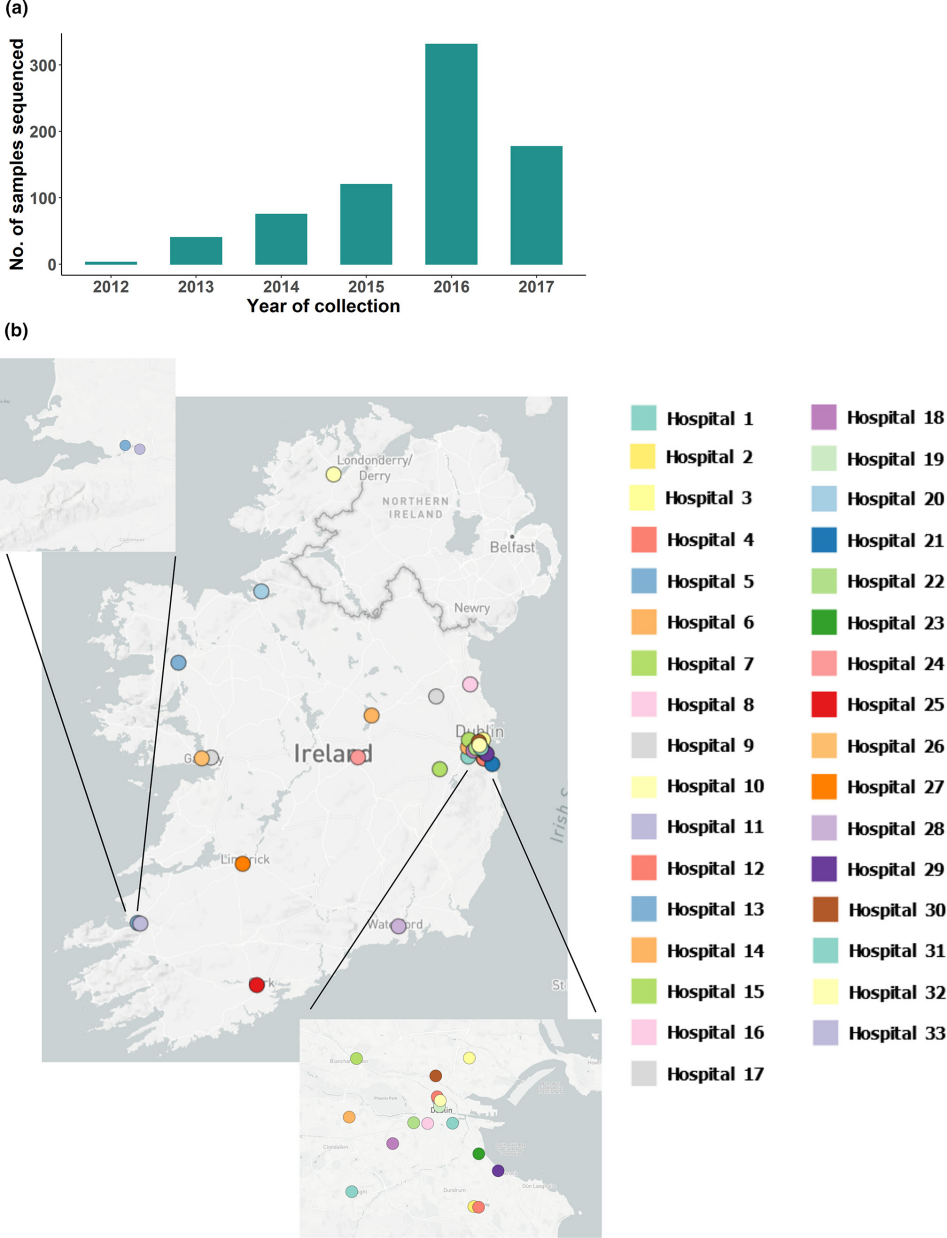
Collection details of 746 CPE samples from 2012 to 2017 that were sequenced and analysed. (a) The frequency of the absolute count of the samples collected between April 2012 and June 2017*. (b) The geographical locations of participating hospitals from which the samples were collected. *Data for 2017 are limited to the first 6 months of the year and thus are not representative of the trend in 2017.

The sampling sites of the invasive samples are summarized in Table S1 (available with the online version of this paper). Geographically, the samples were from hospitals in the following counties: Dublin (*n*=18), Kerry (*n*=2), Galway (*n*=2), Donegal (*n*=1), Mayo (*n*=1), Westmeath (*n*=1), Kildare (*n*=1), Louth (*n*=1), Meath (*n*=1), Sligo (*n*=1), Offaly (*n*=1), Cork (*n*=1), Limerick (*n*=1) and Waterford (*n*=1). They were subsequently designated Hospitals 1–33 for the purposes of this analysis. Fig. 1(a, b) illustrate the sample collection details.

### Antimicrobial susceptibility testing

Antimicrobial susceptibilities were evaluated for a total of 13 antibiotics, using established methods. The antimicrobial agents tested were ampicillin, amikacin, amoxicillin/clavulanic acid, cefpodoxime, cefpodoxime/clavulanic acid, cefoxitin, cefotaxime, ciprofloxacin, ertapenem, gentamicin, meropenem, piperacillin/tazobactam and tigecycline. Details of the procedures are given the supplementary text.

### Sequencing, taxonomic identification, multilocus sequence typing and annotation

DNA extractions were carried out as previously described [20] and are provided in detail in the supplementary text. Illumina Library preparation was carried out according to the methods described by Quail *et al*. [21]. Whole genomes were sequenced on the HiSeq 2000 following the manufacturer’s instructions (Illumina) at the Welcome Sanger Institute, Cambridge, UK, to generate 100 bp paired-end reads. The sequence reads for a further 165 isolates sequenced by the CPE Reference Laboratory, Ireland, were retrieved from the European Nucleotide Archive. Raw reads were filtered and trimmed for bases with phred quality >20 (trimmomatic v0.36.4). Filtered reads were used to generate *de novo* genome assemblies for each strain using SPAdes v3.14.0 [22]. Taxonomic identity was assigned using Kraken [23] for all species other than *Klebsiella* spp. and Kleborate [24] for *Klebsiella* spp. multilocus sequence types (MLSTs) were assigned using mlst_check [25]. In Ireland, library preparation was carried out using illumina Nextera DNA Library Preparation kits (Illumina) followed by illumina MiSeq sequencing to generate 301 bp paired-end reads.

### Identification of carbapenem resistance determinants

We used ARIBA v2.10.0 [26] to identify carbapenem resistance determinants directly from the filtered paired-end sequencing reads using reference databases from both CARD [27] and ResFinder [28]. The sequence identity threshold to define presence or absence of a resistance genotype against a reference was set at 90 %. The tool relies on mapping the reads to reference sequence clusters followed by the formation of local assemblies of the mapped reads and is further able to confirm the intactness of resistance genes.

### Population cluster analysis

PopPUNK (v2.2.0) [29] was performed as a ‘top-level’ approach to rule out transmission by splitting the population into genetically and temporally distant groups. Each species, as defined by Kraken, was assigned into variable-length-k-mer clusters, or VLKCs using PopPUNK. k-mer lengths of 15, 17, 19, 21, 23, 25, 27 and 29 were used to determine core and accessory distances using pp-sketchlib (v1.5.1), with a sketch size of 10 000. These defaults have been shown to work well for bacterial genomes of this length and within species [29].

Six genome assemblies were removed due to being more than five standard deviations away from the average length. For each species we followed a forward process of model refinement, stopping when a good fit to the population was found:

1. Fit the Gaussian mixture model (GMM) [1] with a number of components (--K), chosen by inspection of the distance plot.
2. Fit the DBSCAN model (--dbscan).
3. Use the refine fit mode to find a boundary, also looking at core-only or accessory-only clusters (--indiv-refine).
4. Define clusters using nearest neighbours (--lineage-clustering).

During PopPUNK variable-length-k-mer clustering, six genome assemblies that did not fit in with the default settings, i.e. for being more than five standard deviations away from the average length, were removed. The appropriateness of each species’ distance cluster fit was judged by having a reported network fit score of ≥0.9, and minimal polyphyly when placed on the neighbour-joining tree of core distances. *C. freundii *, *E. cloacae* and *K. oxytoca* were fit using a GMM (step 1). *E. hormaechei* and *E. coli* were fit with a model in refine mode (step3), as was *K. pneumoniae *, but using core distances only as including the accessory distances yielded a worse network score, and a vertical shape of the distances suggested this was an appropriate choice. *Citrobacter* sp*. FDAARGOS_156* showed little diversity, and did not fit well even with step 4. We increased the sketch size to 3×10^5^, but still found zero distances in the data. As this dataset consists of one genetic clone, the PopPUNK model did not fit well for *C.* sp*. FDAARGOS_156* and was not used for further analysis. PopPUNK-assigned clusters were designated as VLKCs.

### Reconstruction of phylogenetic trees

We reconstructed maximum-likelihood phylogenies for each VLKC that had four or more isolates in *K. pneumoniae *, *E. coli *, *K. oxytoca *, *E. hormaechei *, *E. cloacae* and *C. freundii *. In cases where there were a large number of VLKCs that contained two to three isolates, for example in *K. pneumoniae *, *E. coli* and *K. oxytoca *, a single tree per species was generated using these isolates. Isolate sequence data were mapped to appropriate reference genomes (Table S2) using Snippy v4.4.0 [30]. Briefly, the reference genomes for *K. pneumoniae* VLKCs 1, 2 and 3 [11, 31, 32], *E. coli* VLKCs 1 and 2 [33, 34], and *C. freundii* VLKC 1 [35] were based on previously published studies. Complete genomes in NCBI, where available, were used for the others. The NCBI genomes were checked for quality based on their metrics available on the European nucleotide archive, prior to usage. Additionally, the taxonomic identities and MLSTs were verified using tools from the Centre for Genomic Epidemiology (https://www.genomicepidemiology.org). For clusters where no reference genomes were available, the best available *de novo* assembly of the most common ST within a VLKC was used, based on assembly metrics generated using quast v5.02 [36]. The main parameters assessed for best availability were draft genomes with the highest N50 values and largest sized contigs but with the least number of contigs, mismatches and indels. Quality parameters for all references are given in Table S3.

Regions of recombination within the alignments were identified using Gubbins v2.4.1 [37] and were masked using understandingGubbins.py (https://github.com/jameshadfield/understandingGubbins). snp-sites v 2.5.1 [38] was used to calculate the number of variant sites in the resulting alignments. These were subsequently used in the reconstruction of high-resolution maximum-likelihood phylogenies with IQ tree v2.1.2 [39] implementing the built-in model testing (-m MFP) to determine the best phylogenetic model and 1000 ultrafast bootstraps (-bb 1000). Details of the phylogenetic tree models are given in Table S2. Pairsnp v0.2 ·0 (https://github.com/gtonkinhill/pairsnp) was used to calculate pairwise SNP distances between isolates from the filtered polymorphic sites alignment files. Trees were visualized in iTOL (https://itol.embl.de).

### Transmission analysis

Transmission analysis was performed for three reasons: first, to identify clones circulating within hospitals; second, to identify putative recent (within 90 days) transmissions that occurred within and between hospitals; and third, to identify potential outbreaks. For analysis, we first split the population into genetically distanced groups (VLKCs) between which transmissions can be ruled out. We followed this by reconstructing SNP-based phylogenies of the VLKCs and then identified circulating clones within those VLKCs. Next we derived isolate pairwise SNP distance thresholds for all six *Enterobacterales* species. This then allowed us to perform a finer scaled transmission analysis within each circulating clone. A conceptual flow diagram showing the transmission analysis is given in Fig. S1. Of note, transmission analyses including outbreak investigations were performed separately for each circulating clone (numbered numerically) of a VLKC (representing a single ST or multiple STs) using a threshold.

### Determination of circulating clones

We defined a circulating clone as a group of isolates with an isolation date overlap of maximum 2.5 years that resided in a monophyletic clade in a VLKC of interest. When assigning monophyletic clades in nosocomial *K. pneumoniae *, SNP threshold values such as 16 (over 1 year), 24 (over 2 years), 21 (over 6 months) and 13–214 (over 20 years) have been reported [11, 13, 40, 41]. We determined the circulating clones manually by looking at the core genome maximum-likelihood phylogenies in combination with epidemiological data. Based on our grouping, the minimum and maximum pairwise SNP distance of isolates in a clone were 0 and 39. Specifically, the minimum and maximum pairwise SNP distance of isolates in a clone were 0–16 (over 2.5 years) in *K. pneumoniae *, 0–18 (over 1 year) in *E. coli *, 0–10 (over 2.5 years) in *K. oxytoca *, 0–39 (over 1 year) in *E. hormaechei *, 6–24 (over 1 year) in *E. cloacae* and 7–19 (over 1.5 years) in *C. freundii *.

### Definition of transmission events

For the purpose of this analysis, we define a single transmission event as one that occurs between two patients consisting of two closely related samples, a small-scale transmission event as one that consists of one or two transmission events (two or three patients) while an outbreak is an event composed of five or more transmissions (a minimum of four patients) (Fig. S1). Detection of putative recent transmission links between isolate pairs was limited to 90 days. The choice of a 90 day window was arbitrary and was selected to be conservative in the proportion of putative transmissions ruled out as well as allowing for a fine-scale resolution of transmission.

### Determination of transmission thresholds

Thresholds for identifying transmission events [42] were determined using (1) published data from within–host diversity information, where available, or (2) using estimates of pairwise SNP distance distributions (Fig. S2) that would capture 95 % of cases within 21 SNPs [11] isolated within 90 days (Table S4). An SNP threshold of 21 has been used previously to discriminate between hospital clusters in *K. pneumoniae* over a 180 day period [11] and we have adopted this as the boundary, for all *Enterobacterales* species studied, within which to derive thresholds for recent (90 days) transmissions. In *K. pneumoniae* and *E. coli *, where published within–host diversity informed transmission thresholds, the values were further compared with those derived from the pairwise SNP distance distributions for validation.

In method 1, we added the published within–host diversity SNP number to the number of SNPs expected to accumulate over 3 months, in the source and recipient hosts. For example, in *K. pneumoniae* ST258/512, the within–host diversity of 7 SNPs [43] was added to 2.5 core genome SNPs (substitution/core genome/3 months rounded to 5)×2, calculated from published substitution rates [44], which resulted in a threshold of 12 SNPs (Table S4). In method 2, we plotted the distribution of SNP distances vs. isolate pairs for epidemiologically linked patients, i.e. cases within 21 SNPs isolated within 90 days, and determined as thresholds the SNP distances that captured 95 % of epidemiologically linked cases. For example, in *K. pneumoniae* ST258/512 the 95th percentile value of the isolate pair distribution was 12 SNPs (Fig. S3).

The plots for determination of threshold for epidemiologically linked cases for all six *Enterobacterales* species, based on method 2, are illustrated in Fig. S3. Of note, for *K. pneumoniae *, ST258/ST512 the method 1 threshold value were directly compared with that of method 2 by deriving the value from corresponding STs. The value of 18 SNPs was used as the threshold for *E. coli* based on method 1 (Table S4). For method 1, the *E. coli* threshold was based on *E. coli* ST73 while the method 2 value was derived from all *E. coli* STs due to lack of corresponding STs in the collection and hence the two values could not be directly compared. For every other species, isolates from all STs studied were examined as a whole and thresholds determined based on method 2.

### Inference of putative transmission events

Putative within–hospital transmissions were reported if the following criteria were met: samples (1) had pairwise SNP distances below the thresholds in Table S4, (2) belonged to the same ST, (3) carried the same carbapenemase gene, (4) were isolated from the same hospital, and (5) showed an isolation date overlap of 3 months. The same criteria were used for defining between–hospital transmissions, with the exception that isolates must be collected from different hospitals.

## Results

### Temporal changes in CPE prevalence

Nineteen CPE species were identified within our dataset, which were collected from 33 hospitals. *K. pneumoniae* prevalence was the highest (243/746, 32.6%) followed by that of *E. coli* (191/746, 25.6%), *K. oxytoca* (87/746, 11·6%), *E. hormaechei* (76/746, 10.2%), *C. freundii* (42/746, 5.6%), *C. sp. FDAARGOS_156* (31/746, 4.2%) and *E. cloacae* (31/746, 4.2%). The prevalence of the remaining 12 species was less than 3 %. Hospital 1 in Dublin (36.1%), Hospital 27 in Limerick (21.7%), Hospital 28 in Waterford (8.4%) and Hospital 26 in Galway (6.5%) accounted for more than 50 % of the total CPE isolates collected during the study period.

While a large number of unique STs were identified among *E. coli* (*n*=88 in 191 isolates), *K. pneumoniae* (*n*=70 in 243 isolates), *K. oxytoca* (*n*=27 in 87 isolates), *C. freundii* (*n*=23 in 42 isolates) and *E. cloacae* (*n*=14 in 31 isolates), only a few different STs were detected in *E. hormaechei* (*n*=7 in 87 isolates) and *C.* sp*. FDAARGOS_156* (*n*=2 in 31 isolates) collections. A large proportion of carbapenemase-positive *K. pneumoniae* and *E. coli* were represented by minor STs. All STs are described in Supplementary Table S5. Here we define ‘common’ STs as STs representing >5 % of the isolates in a species and ‘minor’, when present below this threshold.

**Fig. 2.**
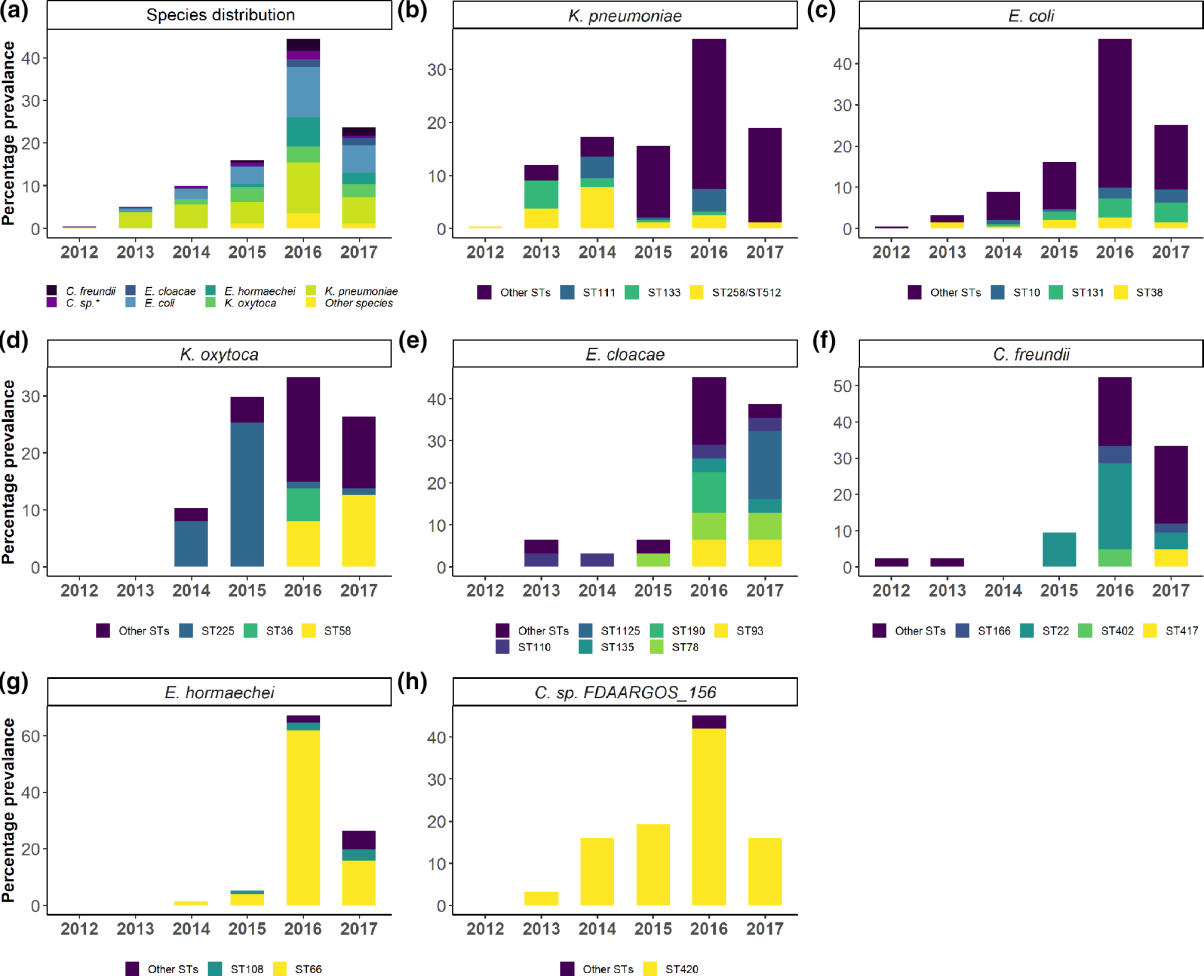
The prevalence of *Enterobacterales* species in Ireland between April 2012 and June 2017*. (a) The distribution of all species. (b–h) The distribution of *Klebsiella pneumoniae *, *Escherichia coli *, *Klebsiella oxytoca *, *Enterobacter cloacae *, *Citrobacter freundii *, *Enterobacter hormaechei* and *Citrobacter* sp*. FDAARGOS_156*, respectively. Other STs denote ‘minor’ STs that represent STs that fall below <5 % of the isolates in a species. *Data for 2017 are limited to the first 6 months of the year and thus are not representative of the trend in 2017.

Four STs (ST258/512, ST111 and ST33) were identified as common *K. pneumoniae* STs in Ireland, accounting for 32·6 % of *K. pneumoniae* isolates (Table S6). ST131, ST38 and ST10 were common in *E. coli* accounting for 27.6 % of *E. coli* isolates (Table S6). An increase in the prevalence of *K. pneumoniae* and *E. coli *, overall, was evident (Fig. 2a), which could be mainly attributed to the increase in minor STs (Fig. 2b, c and Table S5). The percentage increases of the prevalence of minor STs from 2015 and 2016 were large, i.e. about twofold (32 to 69 isolates) and about threefold (22 to 69 isolates) in *K. pneumoniae* and *E. coli *, respectively. New STs appear to have replaced existing ones in *K. oxytoca *, *E. cloacae* and *C. freundii* from 2016 onwards whereas expansion of existing clones was noted in *E. hormaechei* and *C.* sp*. FDAARGOS_156* (Fig. 2).

### Prevalence of carbapenemases in Ireland

Almost all (729/736, 99.0%) of the isolates were resistant to three or more of the beta-lactams tested. By contrast, based on 732 isolates that had aminoglycoside and 677 that had tigecycline susceptibility data, 329 (44.9%) and 308 (45.4%) were susceptible to at least one aminoglycoside (gentamicin, amikacin or tobramycin) and tigecycline, respectively (Tables S6 and S7).

**Fig. 3.**
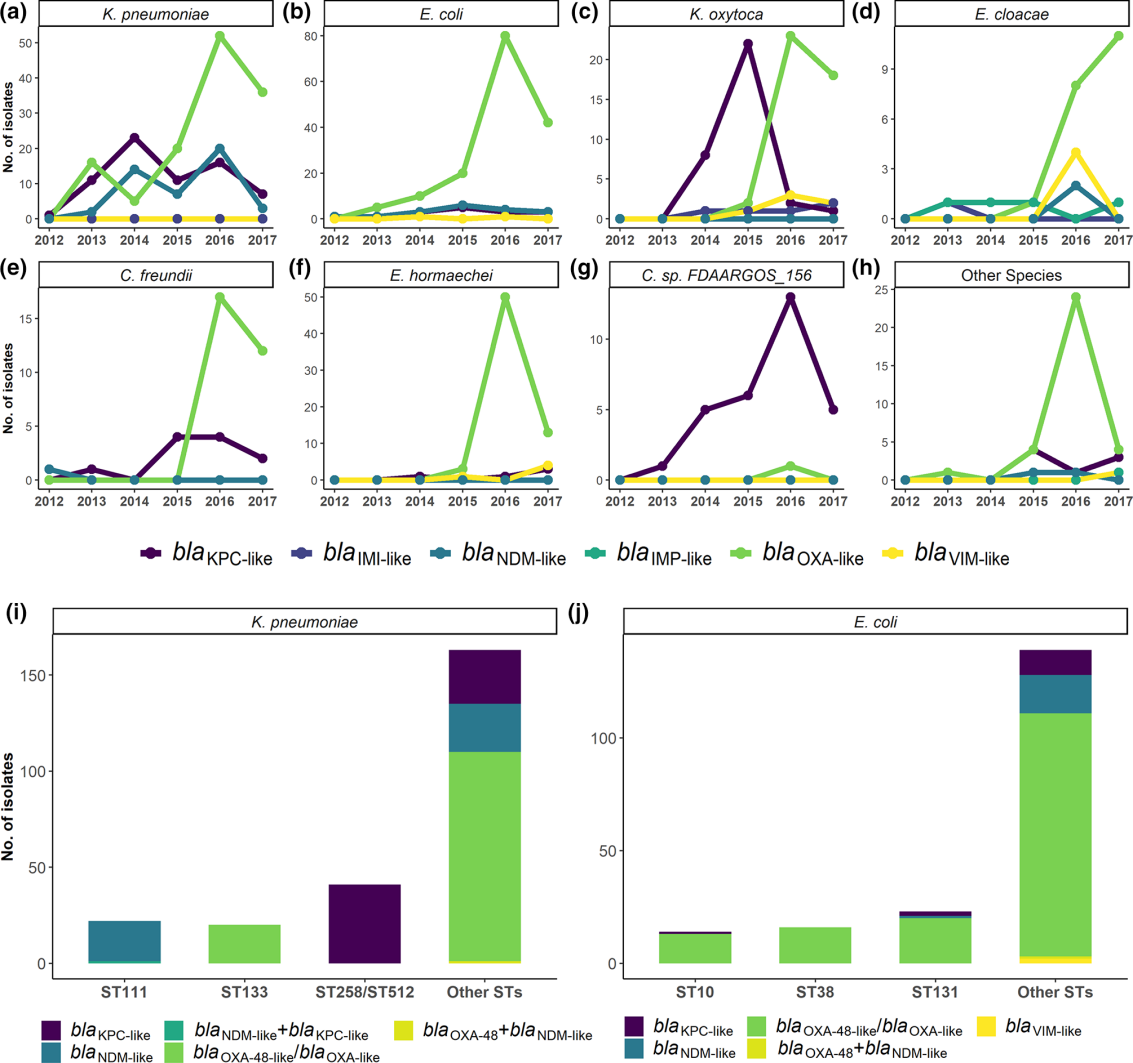
Carbapenemase prevalence trends. (a–h) Distribution of carbapenemases among *Enterobacterales *, Ireland, April 2012 to June 2017*. (i, j) The frequency of carbapenemases by ST in *K. pneumoniae* and *E. coli. bla*_KPC-like_ represents *bla*_KPC-2_ and *bla*_KPC-3_, *bla*_NDM-like_ represents *bla*_NDM-1_, *bla*_NDM*-5*_ and *bla*_NDM-19_, *bla*_OXA-like_ represents *bla*_OXA-48_, *bla*_OXA-181_, *bla*_OXA-244_ and *bla*_OXA-162_ in (a) to (h) and *bla*_OXA-181_, *bla*_OXA-244_ and *bla*_OXA-162_ in (i) and (j). *Data for 2017 are limited to the first 6 months of the year and thus are not representative of the trend in 2017.

Our *in silico* examination of the draft genomes revealed 15 gene variants of carbapenemases, with *bla*_OXA-48_ (*n*=459) being identified as the most prevalent carbapenemase gene (Table S8). There were three isolates that had more than one carbapenemase gene. Unlike *bla*_KPC-like_ and *bla*_NDM-like_, prevalence of *bla*_OXA-48_/*bla*_OXA-like_ carriage increased steadily in all species except *C.* sp*. FDAARGOS_156* up to 2016 (Fig. 3a–h). Overall, *bla*_OXA-48_ */bla*_OXA-like_ was present in 63.4 % of all CPEs included in this study, suggesting this carbapenemase family to be the main cause of carbapenem resistance in hospital infections.

In *K. pneumoniae *, the common STs, ST258/512, ST133 and ST111, were each associated with *bla*_KPC-2_/*bla*_KPC-3_, *bla*_OXA-48_ and *bla*_NDM-1_ determinants, respectively. In contrast, the majority (108/161, 60.0%) of the minor STs carried *bla*_OXA-48_/*bla*_OXA-like_ (Fig. 3i). In *E. coli* both the commonly occurring STs (49/53, 92.4%) as well as the minor STs (109/138, 78.9%) were predominantly OXA-48 producers (Fig. 3j).

### Within–hospital transmissions and outbreak events present in most species

Next, we sought evidence of within– and between–hospital transmission events. We detected molecular evidence, supported by epidemiological data, that indicated putative small-scale within–hospital transmissions (two or three patients) in all six species as well as outbreaks (i.e. five or more transmissions per case involving closely related samples from a minimum of four patients) in 4/6 species. Putative between–hospital transmission events were fewer in number than within–hospital transmission events in four species. Of the 172 isolates that contributed to putative transmission events, two (one each of *E. hormaechei* and *C. freundii *) were associated with patient environments. The results of the analysis are shown in Table 1.

**Table 1.** Within– and between–hospital putative transmission events in six *Enterobacterales* species isolated in Ireland from 2012 to 2017

Species	Variable-length k-mer cluster (no. of isolates)	Sequence type (ST)	No. of clones	No. of putative transmission events (clone identifier)*	Total within–hospital events	Total between–hospital events	Total putative outbreaks
* K. pneumoniae *	1 (53)	ST258/512/11	10	4(1), 7(2), 1(3), 1(4), 3(5), 6(6), 1(7), 1(8), 10(10)	26	8	2
* K. pneumoniae *	2 (21)	ST111	2	28(1), 18(2)	46	0	2
* K. pneumoniae *	3 (20)	ST133	3	3(1), 2(2), 3(3)	6	2	0
* K. pneumoniae *	4 (15)	ST20/336/3515	2	6(1), 8(2)	14	0	2
* K. pneumoniae *	5 (10)	ST14/15/3514	0	0	0	0	0
* K. pneumoniae *	6 (9)	ST323	2	5(1), 2(2)	5	2	1
* K. pneumoniae *	7 (9)	ST37/309	2	3(2)	3	0	0
* K. pneumoniae *	8 (6)	ST26	1	7(1)	7	0	1
* K. pneumoniae *	9 (6)	ST36/834	0	0	0	0	0
* K. pneumoniae *	10(6)	ST922	2	1(2)	1	0	0
* K. pneumoniae *	11(5)	ST1307	1	2	2	2	0
* K. pneumoniae *	12(5)	ST23	1	1	1	0	0
* K. pneumoniae *	13(4)	ST48	1	10	10	0	1
* K. pneumoniae *	14(4)	ST29	1	1	1	0	0
* K. pneumoniae *	15(4)	ST147	1	0	0	0	0
* K. pneumoniae *	Others (58)	Multiple	4	1, 1, 1	3	0	0
* E. coli *	1(25)	ST10	4	3(2)	4	0	0
* E. coli *	2 (24)	ST131	4	1(2), 2(3), 5(4)	8	0	1
* E. coli *	3 (16)	ST38	3	0	0	0	0
* E. coli *	4 (13)	ST410	1	0	0	0	0
* E. coli *	5 (9)	ST58	1	1	1	0	0
* E. coli *	6 (5)	ST162	1	0	0	0	0
* E. coli *	7 (5)	ST12	0	0	0	0	0
* E. coli *	8 (5)	ST69	1	1	1	0	0
* E. coli *	9 (4)	ST401	0	0	0	0	0
* E. coli *	Others (54)	Multiple	2	1(1), 1(2)	2	0	0
* K. oxytoca *	1 (26)	ST225_1	6	11(1), 1(3), 1(4), 14(6)	27	0	2
* K. oxytoca *	2 (18)	ST58	3	6(1), 19(2), 3(3)	28	0	2
* K. oxytoca *	3 (5)	ST36	1	10(1)	10	0	1
* K. oxytoca *	4 (5)	ST225_2	1	4(1)	4	0	0
* K. oxytoca *	Others (14)	multiple	0	0	0	0	0
* C. freundii *	1 (17)	ST22	5	3(2), 1(4)	3	1	0
* E. cloacae *	1 (12)	ST1125/190/93	2	6(2)	3	3	0
* E. cloacae *	2 (5)	ST78	1	0	0	0	0
* E. cloacae *	3 (4)	ST110	0	0	0	0	0
* E. hormaechei *	1 (61)	ST66	10	3(1), 6(2), 2(3), 3(4), 6)7), 3(8), 86(9), 5(10)	116	2	4
* E. hormaechei *	2 (8)	ST108	0	0	0	0	0

*Clone numbers are reported only for variable-length k-mer clusters (VLKCs) that had four or more isolates.

### *K. pneumoniae* epidemiology

**Fig. 4.**
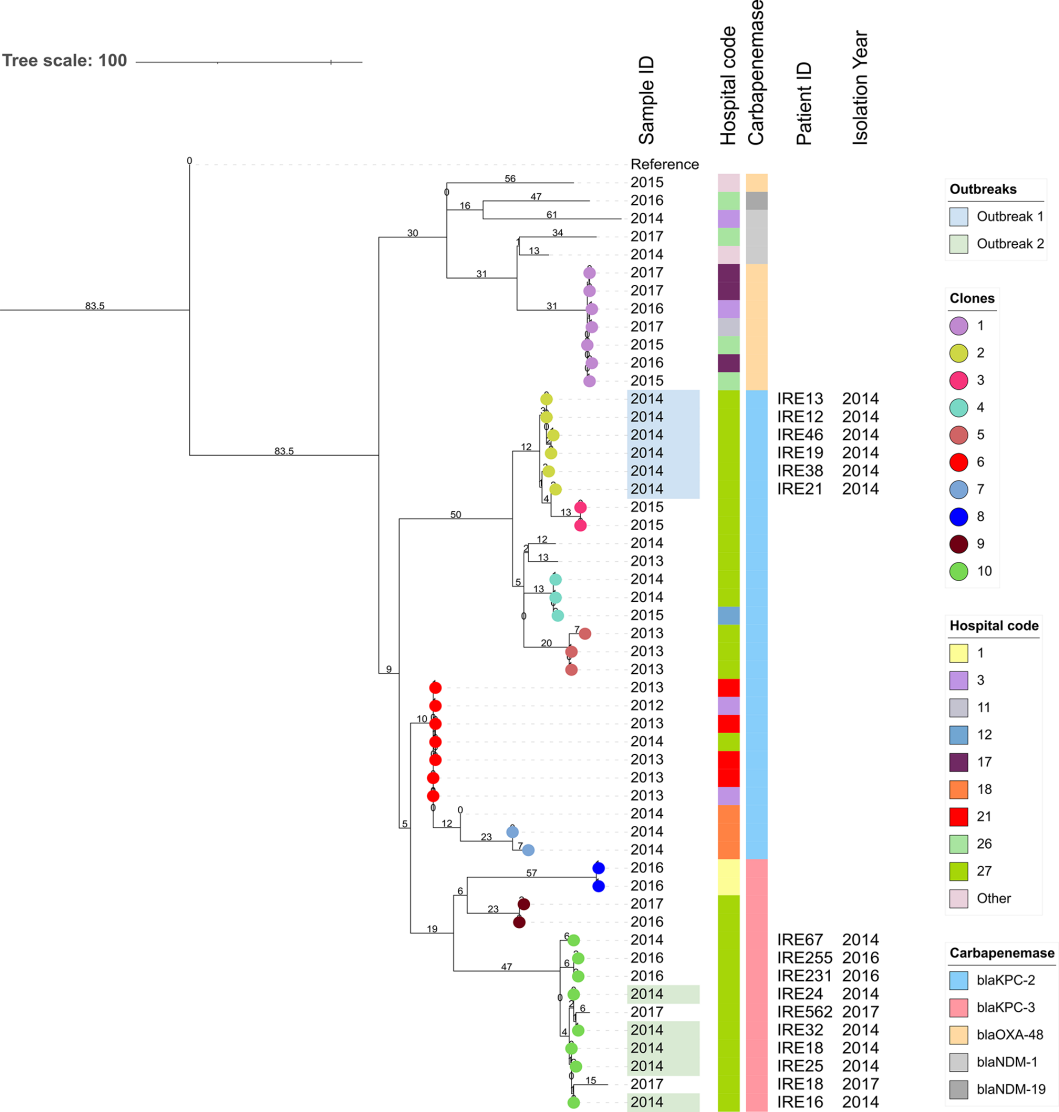
Maximum-likelihood phylogeny of *Klebsiella pneumoniae* VKLC 1 (ST258/512/11). The tree is rooted at the midpoint. The ST258 reference genome CP006923 was used for read mapping. The majority of the main internal tree nodes were supported by ≥99 % bootstrap support (data not shown). The branch lengths are measured in SNPs and are indicated at the nodes, with putative outbreak events highlighted in blue and green. Hospital codes and the carbapenemase enzyme types present within circulating clones (coloured circles) are annotated. Patient IDs and isolation year of isolates in clones associated with outbreak events are also given. An outbreak is defined as an event composed of five or more transmission events involving a minimum of four patients while a single transmission event is one that occurs between two patients consisting of two closely related samples. Detection of putative recent transmission links between isolate pairs was limited to 90 days when inferring outbreaks. We defined a circulating clone as a group of isolates that resides in a monophyletic clade in a VLKC with an isolation date overlap of maximum 2.5 years. VLKC, variable-length-k-mer cluster (assigned by PopPUNK [29].

When determining SNP threshold values to discriminate transmission events, we found that the threshold value of 12 SNPs, derived from published [43] within–host diversity data was the same as that of the second method involving pairwise distance distribution for *K. pneumoniae* (Figs S2 and S3). A total of 32 circulating clones were identified in the 15 main (that had four or more isolates per cluster) *K. pneumoniae* VLKCs. This included 10 clones within VLKC 1 (ST258/512/11, *n*=53 isolates, Fig. 4), i.e. VLKC 1 had 10 monophyletic clades each of which contained groups of isolates with an isolation date overlap of maximum 2.5 years. A total of 140 putative transmissions (Table 1) were observed in *K. pneumoniae *, where five VLKCs contributed to the majority (113/140, 80·7%) of events while six, including VLKC 1 (Fig. 4), showed evidence of putative outbreaks (Table 1).

We also observed 14 putative between–hospital transmissions in five *K. pneumoniae* VLKCs. For example, in VLKC 1 (Clone 1), two patients from Hospital 17 (Dublin) yielded isolates that were closely related to an isolate from a patient in Hospital 11 (Meath) and Hospital 3 (Dublin) within a period of 90 days. Furthermore, Hospital 27 in Limerick had the largest number of transmissions (58/140, 41.4%) including four outbreaks, followed by Hospital 28 in Waterford with two outbreaks (22/140, 15.7%). Among the four outbreaks in Hospital 27, two were NDM-1 specific whereas the other two were KPC-2 and KPC-3 specific, respectively. In Hospital 28 the two outbreaks were caused by either OXA-48 or NDM-1 producers.

### *E. coli* epidemiology

**Fig. 5.**
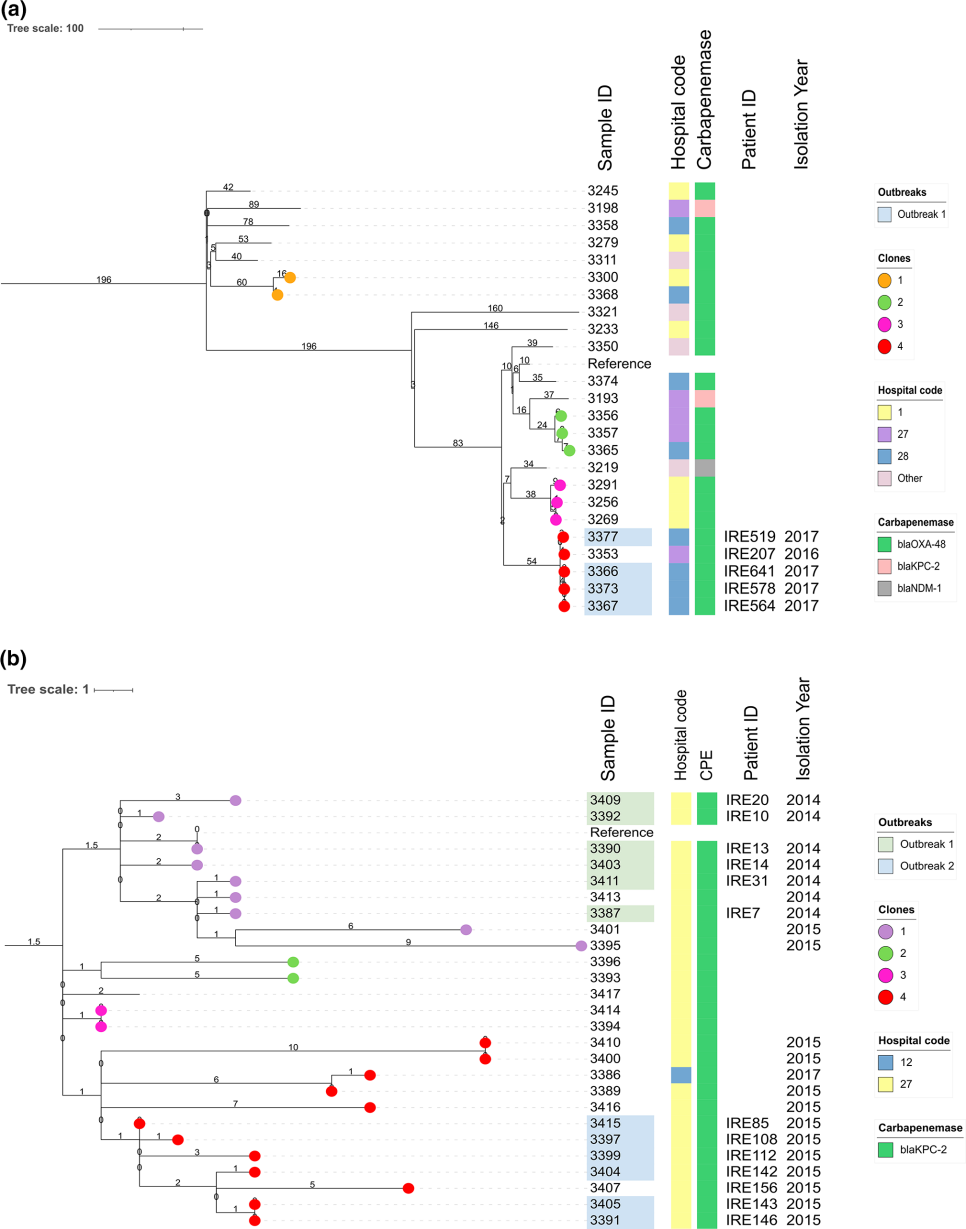
Maximum-likelihood phylogenies of *Escherichia coli* VKLC 2 (ST131) and *Klebsiella oxytoca* VKLC 1 (ST225_1). The trees are rooted at the midpoint. (a) *E. coli* strain NCTC13441 (GenBank Accession no.: NZ_UFZF01000006·1) was used as the reference genome (Reference) for read mapping. The majority of the main internal tree nodes were supported by ≥87 % bootstrap support (data not shown). (b) *K. oxytoca* tree with isolate 3390 used as the reference genome (Reference) for mapping. The majority of the main internal tree nodes were supported by ≥74 % bootstrap values (data not shown). In both (a) and (b), the branch lengths are measured in SNPs and are indicated at the nodes, with putative outbreak events highlighted in pastel colours. The trees are annotated with the hospital codes and the type of carbapenemase enzyme present within circulating clones (coloured circles). Patient IDs and isolation year of isolates in clones associated with outbreak events are also shown. An outbreak is defined as an event composed of five or more transmission events involving a minimum of four patients while a single transmission event is one that occurs between two patients consisting of two closely related samples. Detection of putative recent transmission links between isolate pairs was limited to 90 days when inferring outbreaks. We defined a circulating clone as a group of isolates that resides in a monophyletic clade in a VLKC with an isolation date overlap of maximum 2.5 years. VLKC, variable-length-k-mer cluster (assigned by PopPUNK [29]).

Similar to *K. pneumoniae *, we compared the SNP threshold values for *E. coli* transmission derived from the two methods shown in Table S4. The threshold of 18 SNPs obtained for the within–host diversity method was in agreement with the threshold of 15 used in a recent *E. coli* transmission analysis [20]. Compared to *K. pneumoniae *, *E. coli* VLKCs were more diverse and fewer circulating clones (*n*=15) were present overall. The numbers of putative transmission events (*n*=16) and outbreaks (*n*=1, ST131, Fig. 5a) within circulating clones were also fewer.

### *K. oxytoca* epidemiology

*K. oxytoca* isolates in all VLKCs lacked diversity. Based on pairwise SNP distances, there was no clear delineation between isolates obtained above or below the 90 day period (Fig. 5b). Nevertheless, a 17 SNP threshold identified putative transmission events in isolate pairs within 90 days (Fig. S3). We distinguished four, three and one circulating clones in the three main *K. oxytoca* VLKCs: VLKC 1 (ST225_1, spanning 27 transmissions), VLKC 2 (ST58, spanning 28 transmissions) and VLKC 3 (ST36, spanning 10 transmissions), respectively.

VLKC 1 (Fig. 5b) was present in Hospital 27 while VLKCs 2 and 3 were confined to Hospital 1. In total we detected five putative outbreaks, two each among VLKC 1 (2014 and 2015) and 2 (2016 and 2017) and one in VLKC 3 (2016). All isolates involved in the outbreaks had a median pairwise SNP distance ≤10 and an interquartile range of ≤4.5. Taken together, our data suggest that Hospitals 27 and 1 were reservoirs for closely related isolates circulating within their boundaries (or local areas) over a prolonged period of time.

### *E. hormaechei* epidemiology

Like *K. oxytoca *, *E. hormaechei* circulating clones within the two main VLKCs (ST66 and ST108) showed little heterogeneity. We determined a threshold for transmission to be 19 SNPs, but noted that there were isolate pairs with isolation dates of less than 90 days that had SNP distances up to 50 (Figs S2 and S3). Consistent with the larger sample size of VLKC 1 (61/76 isolates, 80·2%), this cluster had the largest number of clones (*n*=10) and the majority (9/10) of which circulated within Hospital 1 (Table 1, Fig. S4). VLKC1 also had the greatest number of putative transmission events (*n*=118) including four outbreaks. Outbreaks 2–4 (Fig. S4) occurred within Hospital 1 with Outbreak 4 (Clone 10) involving 88 potential transmissions. Of note, isolate 3164 within Clone 10 was associated with patient IRE468’s environment. All three outbreak isolates in VLKC 1 were dated between September and November 2016, suggesting their long-term persistence within Hospital 1.

### *E. cloacae* epidemiology

The *E. cloacae* phylogeny demonstrated that all three VLKCs had considerable heterogeneity, similar to *K. pneumoniae* and *E. coli *. A clear delineation was observed between isolates with isolation dates above or below 90 days when using the pairwise distribution graph (Fig. S2). Of the three VLKCs examined, only VLKC 1 (Fig. S5A) had isolates associated with transmission events. Furthermore, an isolate from this cluster was linked to Hospital 4 (Dublin), suggesting between–hospital transmission.

### *C. freundii* epidemiology

Among the four clones in the main VLKC (ST22), three were closely related and were in circulation in Hospital 27. Three putative within–hospital transmissions and one between–hospital transmission event (Hospitals 27 and 9) were observed in the *C. freundii* VLKC (Fig. S5B). Among the three within–hospital events, one sample was associated with patient IRE371’s environment.

## Discussion

Here, we present a retrospective genomic study of CPE in Ireland, conducted to improve understanding of resistance trends and nosocomial transmission dynamics. This study describes national CPE genomic surveillance data representing six species from 2012 to 2017.

First, an important finding of this study was the increase in the number of minor STs of carbapenemase-producing *K. pneumoniae* and *E. coli* during the study period. STs that were present in <5 % of the isolates of each species accounted for the majority of carbapenemase-producing *K. pneumoniae* (66.2%) and *E. coli* (72.2%) during the study period. Given that the majority of our isolates were from colonization, this is in contrast to *K. pneumoniae* where prevalence was highest in invasive isolates in mainland Europe [11] and the UK [45]. In these studies, a collection of dominant or common clones such as ST258 and its single-locus variant ST512 contributed to the majority of carbapenemase-associated infections. However, an abundance of minor STs in carbapenemase-producing *E. coli* (from all isolation sources) has been previously seen in the UK [45] and Spain [46]. Our study highlights the need for surveillance of CPEs from both carriage and infection and from other reservoirs in order to detect emerging high-risk clones. Our observations also suggest that the abundance of minor carbapenemase-producing STs circulating within hospitals may have a probable reservoir in the community or in the environment. The other is that the minor STs represent transient carriage of plasmids that are not stable in those STs.

Second, blaOXA-48/blaOXA-like carbapenemases were the most prevalent carbapenemases identified in all species, except *C.* sp*. FDAARGOS_156,* accounting for 63.4 % of the total CPE up to 2017 and beyond [47, 48] in Ireland, suggesting this to be the main cause of carbapenem resistance. While KPC-like, overall, was the predominant carbapenemase in the mainly invasive *K. pneumoniae* collections in mainland Europe [3, 11], we found OXA-48 to be the most common among invasive (*n*=99, 60.6%) and non-invasive (*n*=140, 47.1%) isolates in Ireland. Irrespective of the isolation source, OXA-48 also appears to be the most common carbapenemase in *E. coli *, both in mainland Europe [3] and the UK [45] as well as in our study. The ST diversity of *K. pneumoniae* and *E. coli* carrying OXA-48-like in our study suggests that horizontal gene transfer might contribute more towards the dissemination of these determinants than successful clonal expansion. However, further plasmid investigations using long read assemblies as opposed to our short read data are warranted to support this hypothesis and such analysis was outside the scope of this study. Of note, however, a preliminary investigation by stand-alone blast of the short read assemblies indicated putative plasmid locations for many of the carbapenem species studied (data not shown).

Finally, we were able to confirm recent transmissions, i.e. within 90 days, in six CPE species, *K. pneumoniae *, *E. coli *, *K. oxytoca *, *E. hormaechei, E. cloacae* and *C. freundii,* as well as putative outbreaks in 4/6 species. At the species level, *K. pneumoniae *, *K. oxytoca* and *E. hormaechei* indicated frequent and widespread within–hospital transmissions. These species were also characterized by a large number of circulating clones, indicative of their success as some of the leading hospital-acquired pathogens. Furthermore, we have shown evidence of hospitals experiencing prolonged persistence and outbreaks of *K. oxytoca* and *E. hormaechei *. Prolonged outbreaks with isolates that lack diversity is suggestive of complex transmission dynamics and probably involves environmental or human reservoirs [15, 49]. Nosocomial transmissions of carbapenemase-producing *K. pneumoniae *, *E. hormaechei* and *E. cloacae* have been reported previously in Europe, North Carolina in the USA, Northern Italy and Queensland in Australia, while prolonged outbreaks involving *E. cloacae* and *K. pneumoniae* have also been documented, and as such our findings agree with current literature [9–11, 13, 15, 43]. Transmission is reported to be less common and somewhat sporadic in carbapenemase-producing *E. coli* and our findings also reflect these observations [18].

Our study has several limitations. Testing for CPE was not performed among hospital staff or in the community. We also lacked patient pathway data that could enhance interpretation of between–hospital transmissions. Sampling of the hospital environment was infrequent during the period 2012 to 2017 and therefore there were few isolates from the environment (e.g. sinks, plumbing). The transmission process typically involves both within–host evolution and transmission between hosts [20, 42, 43, 50]. Within–host diversity data were not available as individual patient (single colony) isolates were sequenced, but threshold values to infer putative transmissions were based on either using published within–host diversity information or pairwise SNP distance distributions. This, in combination with systematic in-depth examination of all circulating clones derived from high-resolution phylogenies using close references, provided added confidence to our interpretations.

In conclusion, we have clarified the dissemination dynamics of six CPE species in Ireland from 2012 to 2017 by combining genomic and epidemiological data. An increase in *K. pneumoniae* and *E. coli* minor STs carrying carbapenemases highlights the need for their enhanced surveillance to monitor the emergence of high-risk clones. Transmission events were frequent among *K. pneumoniae *, *K. oxytoca* and *E. hormaechei* with some hospitals experiencing prolonged outbreaks. Our data on CPE transmissions and the potential causes of carbapenem resistance in Ireland would be of relevance to future infection control and intervention strategies.

## Data Bibliography

The data supporting this work is available under NCBI bioprojects PRJEB19435, PRJEB22264, PRJEB19229, PRJEB24082, PRJEB24083, PRJEB24084, PRJEB24085, PRJEB24086 and PRJEB24087.

## Supplementary Data

Supplementary material 1
