# Supplementary material for: Dissemination of carbapenemase-producing Enterobacterales in Ireland from 2012 to 2017: a retrospective genomic surveillance study
Source: Microb Genom. 2023 Mar 14;9(3):mgen000924. doi: 10.1099/mgen.0.000924 (PMC10132065; doi:10.1099/mgen.0.000924)
Supplement: Supplementary material 1 [file mgen-9-924-s001.pdf]

Supplementary data

Supplementary text

Antimicrobial susceptibility testing

The panel of antimicrobial agents tested were ampicillin, amikacin, amoxicillin/clavulanic acid, cefpodoxime, cefpodoxime/clavulanic acid, cefoxitin, cefotaxime, ciprofloxacin, ertapenem, gentamicin, meropenem, piperacillin/tazobactam and tigecycline. Testing was carried out both at the CPE reference laboratory in Ireland and at the University of Cambridge, UK. The Vitek 2 system (BioMérieux UK Limited, Basingstoke, UK) was used to evaluate the antimicrobial susceptibilities in Cambridge while the disc diffusion method(1) was used in Ireland. The Vitek-2 procedure was according to the manufacturer's protocol and was performed with the AST-N350 card. Additionally, Etests (BioMérieux UK Limited, Basingstoke, UK) in Cambridge and in Ireland were performed to determine minimum inhibitory concentrations (MIC) for meropenem, ertapenem and tigecycline. While testing methods varied between the two countries, the EUCAST (The European Committee on Antimicrobial Susceptibility Testing 2016) clinical breakpoints were used to determine antimicrobial susceptibility (Supplementary Table S6 and S7).

Study sample selection

Of the 840 samples, 94 were excluded from the analysis: 55 due to mismatches between phenotypic bacterial identification and species identification from the sequence data, three that failed to grow, two that did not pass sequencing QC and 34 that were excluded due to discrepancies between the carbapenemases detected by PCR at the reference lab and *in silico* PCR performed with the sequence data. The remaining 746 sequences were included in the subsequent analyses.

DNA extraction

At Cambridge, a single colony from a pure culture was used to inoculate 1ml BHI Broth (Oxoid, Basingstoke, UK) in 2 ml 96 well S- block plates (Qiagen, Valencia, CA, USA) and incubated overnight at 37ºC in air with shaking (2400rpm). Cells from the overnight culture were pelleted by centrifugation at 4000rpm for 5 min at room temperature. After removing the supernatant, the pellet was re-suspended in 1ml sterile PBS. A 200ul aliquot was used for the next step, and the remainder stored at -80°C. Lysozyme/EDTA (10ul) and RNAse (4ul) were added to the wells containing the cells and incubated at 37°C in air with shaking (100 rpm) for 30 mins. Extractions were then carried out in QIAmp 96 plates (Qiagen), using the QIACube (Qiagen, Valencia, CA, USA) according to manufacturer’s instructions. The extracted DNA was quantified using gel electrophoresis. DNA extracts with a minimum concentrations of 500ng DNA per approximately 100ul elute were sent for whole genome sequencing.

In Ireland, 3-5 colonies from a pure culture were re-suspended in 200µl G2 Buffer (Qiagen,Valencia, CA, USA). Extractions were then carried out on Qiagen EZ1 Advanced XL using EZ1 DNA Tissue Kit (Qiagen,Valencia, CA, USA). Samples were eluted in 100µl and 10µl used in genome sequencing procedures.

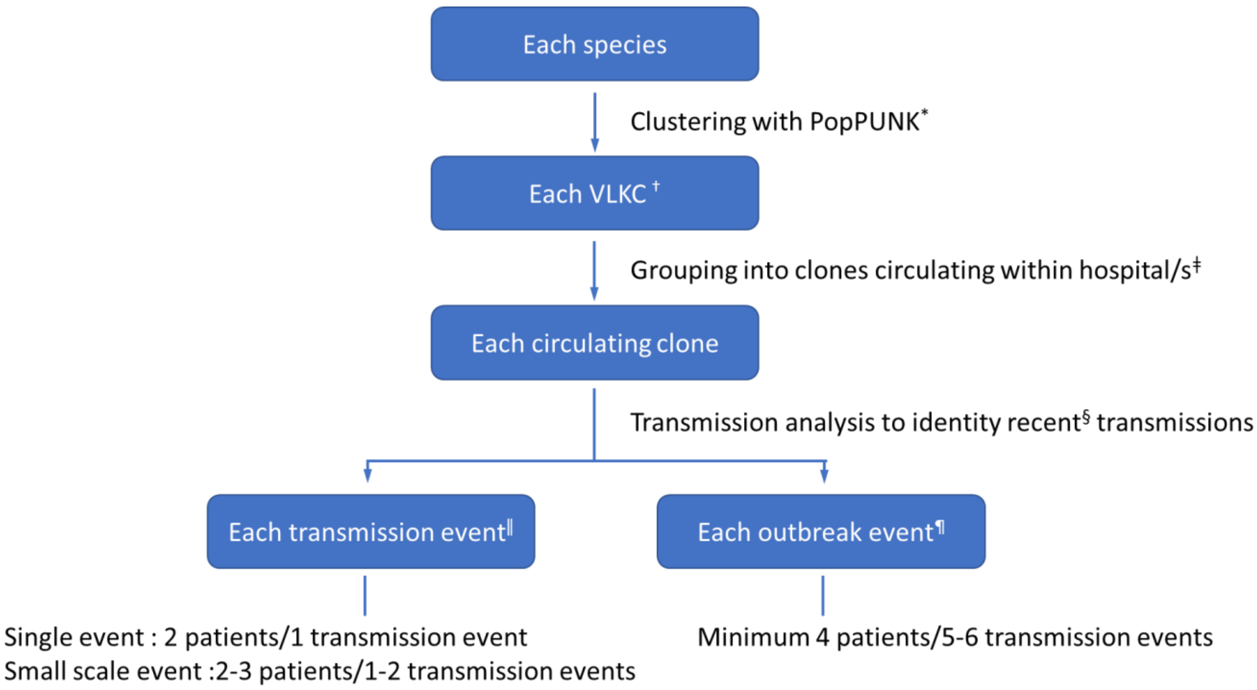

Supplementary Figure S1. Process for inferring within-hospital and between hospital putative transmission events

*PopPUNK software (v2.2.0) (2)

† Each variable-length-k-mer-cluster (VLKC) assigned by PopPUNK may represent one or more closely related Sequence Types (STs)

‡ A circulating clone is a monophyletic clade consisting of isolates with an isolation date overlap of a maximum 2.5 years in a VLKC

§ A recent putative transmission that occurred within 90 days within- and between-hospitals

‖ A putative transmission event that occurs within- and between-hospitals

¶ A putative outbreak event that occurs within a hospital

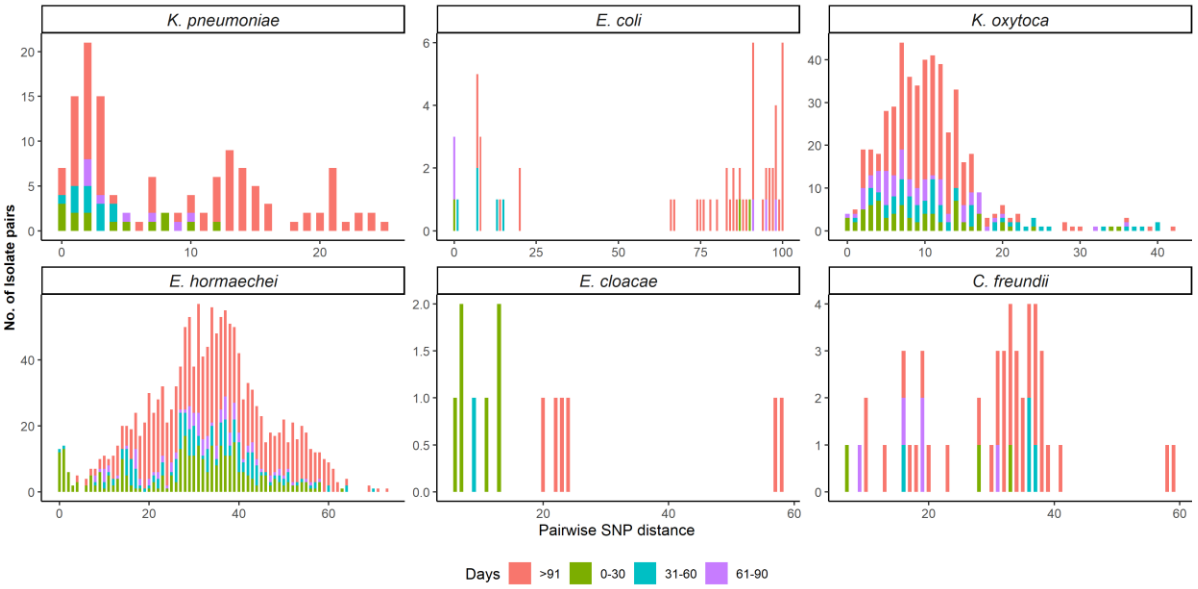

Supplementary Figure S2. The relationship of pairwise SNP distance distribution against number of isolate pairs in six Enterobacterales species. For *Klebsiella pneumoniae*, ST258/ST512 threshold values were directly compared with a corresponding published (3) threshold value by using only the ST258/512 pairwise SNP distance distributions. For every other species, isolates from all STs studied were examined as a whole. SNP = nucleotide polymorphism, ST = sequence type, *K. pneumoniae* = *Klebsiella pneumoniae*, *E. coli* = *Escherichia coli*, *K. oxytoca* = *Klebsiella oxytoca*, *E. hormaechei* = *Enterobacter hormaechei*, *E. cloacae* = *Enterobacter cloacae* and *C. freundii* = *Citrobacter freundii*.

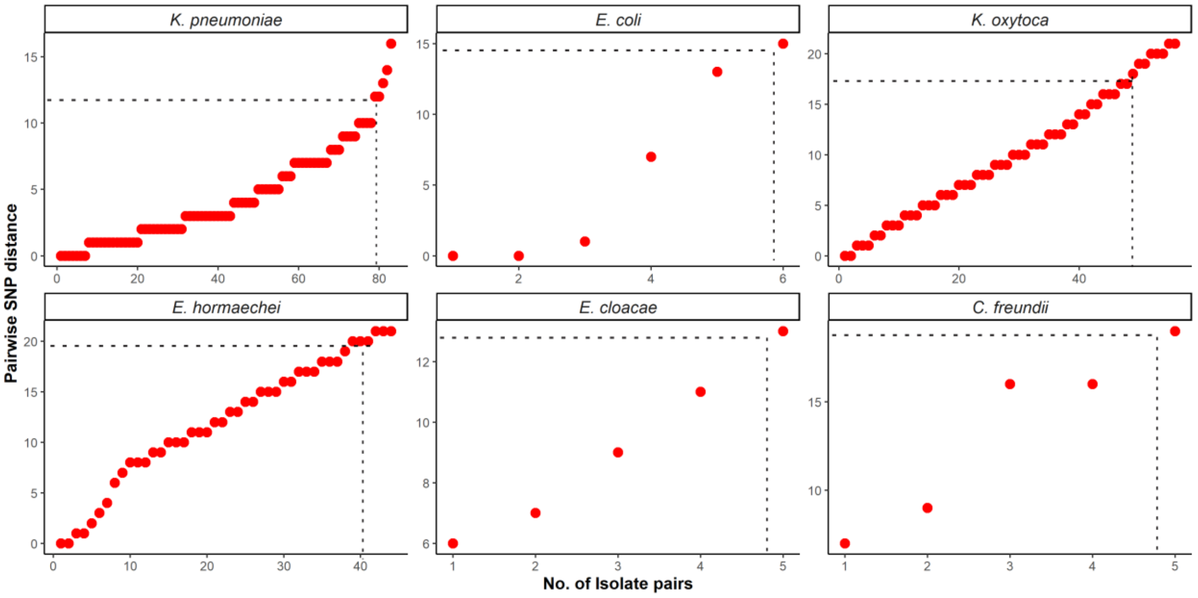

Supplementary Figure S3. Distribution of SNP distances among epidemiologically linked isolate pairs. Isolates less than 90 days apart and within 21 SNPs (4) were defined as epidemiologically linked. Threshold values were SNP distance that captures 95% of epidemiologically linked cases (5). The indicated vertical dotted lines intercept at the 95th percentiles of the number of isolate pairs. The indicated horizontal dotted lines intercept at the pairwise SNP distance values identified as the thresholds. For *Klebsiella pneumoniae*

ST258/512 isolates were used in the threshold derivation while for every other species, isolates from all STs studied were examined as a whole. SNP = nucleotide polymorphism, ST = sequence type, *K. pneumoniae* = *Klebsiella pneumoniae*, *E. coli* = *Escherichia coli*, *K. oxytoca* = *Klebsiella oxytoca*, *E. hormaechei* = *Enterobacter hormaechei*, *E. cloacae* = *Enterobacter cloacae* and *C. freundii* = *Citrobacter freundii*.

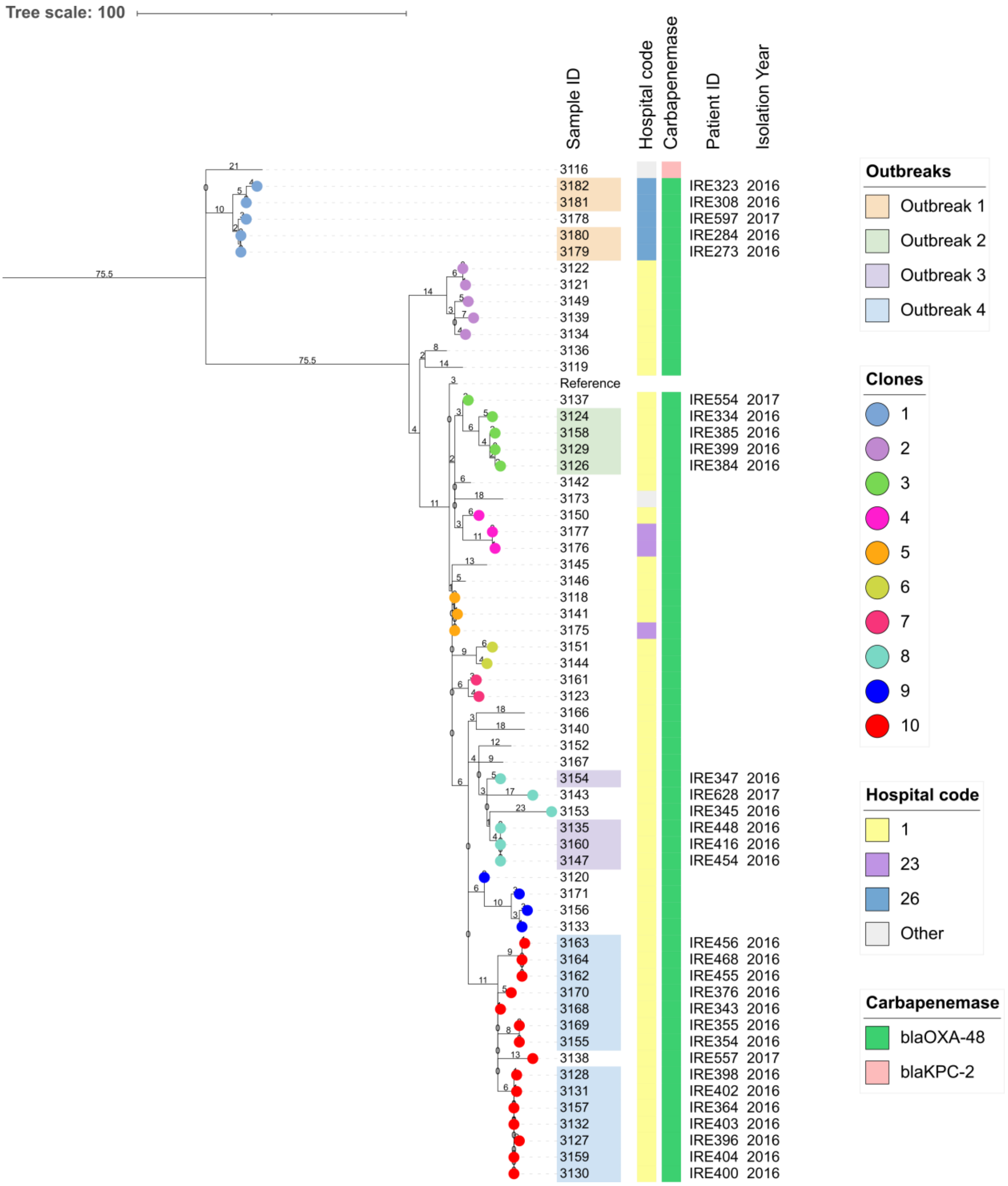

Supplementary Figure S4. Maximum-likelihood phylogeny of *Enterobacter hormaechei* VKLC 1 (ST66). The tree is rooted at the midpoint Isolate 3118 was used as the reference genome (Reference) for read mapping. The majority of the main internal tree nodes were supported by ≥99% bootstrap values (data not shown). The branch lengths are measured in

single nucleotide polymorphisms in the figure and are indicated at the nodes, with putative outbreak events highlighted in orange, green, purple and blue. The tree is annotated with the hospital codes and the type of carbapenemase enzyme present within circulating clones (coloured circles). Patient IDs and isolation year of isolates in clones associated with outbreak events are also shown. An outbreak is defined as an event composed of five or more transmission events involving a minimum of four patients while a single transmission event is one that occurs between two patients consisting of two closely related samples. Detection of putative recent transmission links between isolate pairs was limited to 90 days when inferring outbreaks. We defined a circulating clone as a group of isolates that resides in a monophyletic clade in a VLKC with an isolation date overlap of maximum 2.5 years. VLKC= variable-length-k-mer clusters (assigned by PopPUNK (2)).

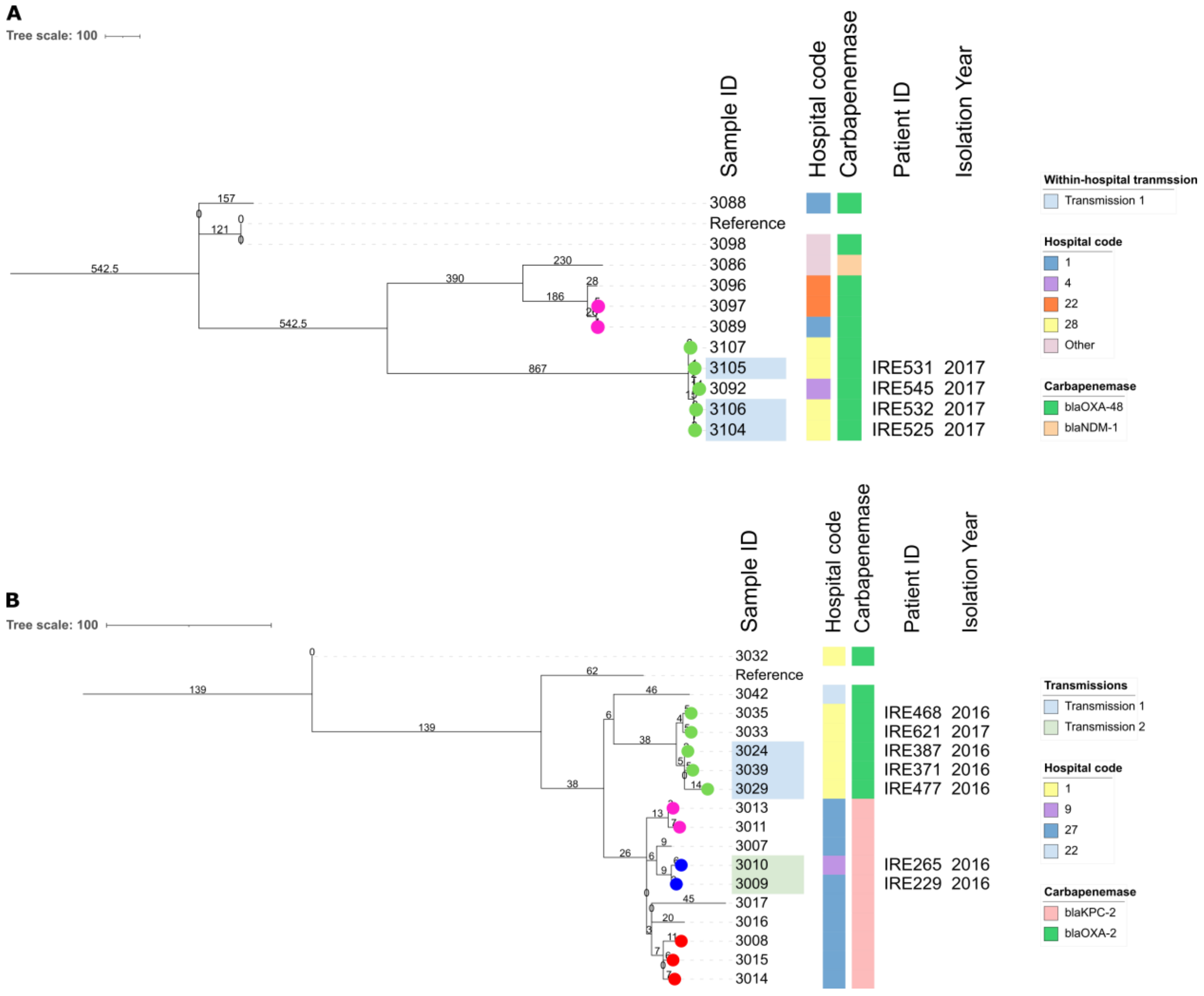

Supplementary Figure S5. Maximum-likelihood phylogenies of *Enterobacter cloacae* VKLC 1 (CC1125) and C*itrobacter freundii* VKLC 1 (ST22) The trees are rooted at the midpoint A. *E. cloacae* tree with isolate 3098 used as the reference genome (Reference) for read mapping. B. *C. freundii* tree, with strain CAV1321 (GenBank Accession no. CP011612.1) used as the reference genome (Reference) for read mapping. The majority of the main internal tree nodes in both Figures A and B were supported by 100% bootstrap values (data not shown). Figures A and B branch lengths are measured in single nucleotide polymorphisms and are indicated at the nodes, with putative small scale transmission events highlighted in pastel colours. The trees are annotated with the hospital codes and the type of

carbapenemase enzyme present within circulating clones (A: Clone 1 =pink circle, Clone 2 = green circle and B: Clone 1 = green circle, Clone 2 = pink circle, Clone 3 = blue circle, Clone 4 = red circle). Patient IDs and isolation year of isolates in clones associated with small scale transmission events are also shown. A transmission is defined as an event that occurs between two patients consisting of two closely related samples and a small scale transmission event as one that consists of 1-2 transmission events (2-3 patients) Detection of putative recent transmission links between isolate pairs was limited to 90 days. We defined a circulating clone as a group of isolates that resides in a monophyletic clade in a VLKC with an isolation date overlap of maximum 2.5 years. VLKC= variable-length-k-mer clusters (assigned by PopPUNK (2)).

Supplementary Table S1. Summary of the sampling sites of the invasive samples included in the study

Sampling site	Number
Abdominal	6
Abscess	1
Bronchoalveolar Lavage	2
Bile	4
Blood	24
Drainage	8
Ears	1
Femoral	1
Foot	1
Gallbladder	1
High vaginal swab	2
Illeostomy	1
Left hip	1
Leg	1
Line Tip	1
Nephrostomy	2
Peritoneum	2
Plaque	2
Pleural	2
Pus	2
Heel	1
Sacrum	3
Sputum	26
Stoma	1
Tip	2
Tissue	2
Toe	2
Urine	116
Vas Cath Tip	1
Vulva	1
Wound	14

Supplementary Table S2. Reference genomes and IQ tree models used in the reconstruction of maximum likelihood trees

Species	Variable length k-mer cluster (VLKC) No. (no. of isolates)	Main Sequence Type (ST)*	Reference Genome (complete genome or best assembly	IQ tree model
K. pneumoniae	1 (53)	ST258	CP006923·1	TVM+F+I
K. pneumoniae	2 (21)	ST111	CP058361·1	HKY+F+I
K. pneumoniae	3 (20)	ST133	CP063939·1	TPM2+F+I
K. pneumoniae	4 (15)	ST20	CP035531·1	TPM3+F
K. pneumoniae	5 (10)	ST15	CP029137·1	TVM+F
K. pneumoniae	6 (9)	ST323	CP024515·1	HKY+F
K. pneumoniae	7 (9)	ST37	CP014762·1	TPM2+F
K. pneumoniae	8 (6)	ST26	3494	HKY+F
K. pneumoniae	9 (6)	ST36	CP028391·1	TVM+F+I
K. pneumoniae	10 (6)	ST922	3677	HKY+F
K. pneumoniae	11 (5)	ST307	CP025143·1	HKY+F
K. pneumoniae	12 (5)	ST23	CP016813·1	K3Pu+F
K. pneumoniae	13 (4)	ST48	CP029388·1	HKY+F
K. pneumoniae	14 (4)	ST29	CP030172·1	K3Pu+F
K. pneumoniae	15 (4)	ST147	CP012745·1	HKY+F+I
K. pneumoniae	Others (30)	multiple	CP006923·1	TVM+F+R2
E. coli	1(25)	ST10	U00096·3	TVM+F+I
E. coli	2 (24)	ST131	NZ_UFZF01000006·1	K3Pu+F+I
E. coli	3 (16)	ST38	3341	K3Pu+F+I
E. coli	4 (13)	ST410	3346	TVM+F+I
E. coli	5 (9)	ST58	3297	K3Pu+F
E. coli	6 (5)	ST162	3315	HKY+F
E. coli	7 (5)	ST12	3276	K3Pu+F
E. coli	8 (5)	ST69	3305	K3Pu+F
E. coli	9 (4)	ST401	3218	K3Pu+F
E. coli	Others (54)	multiple	U00096·3	TVM+F+R2
K. oxytoca	1 (26)	ST225_1	3390	HKY+F+I
K. oxytoca	2 (18)	ST58	3421	F81+F
K. oxytoca	3 (5)	ST36	3433	F81+F
K. oxytoca	4 (5)	ST225_2	3388	F81+F
K. oxytoca	Others (12)	multiple	3390	GTR+F+R10
C. freundii	1 (17)	ST22	CP011612·1	HKY+F
E. cloacae	1 (12)	ST1125	3098	TVM+F+I
E. cloacae	2 (5)	ST78	3103	HKY+F
E. cloacae	3 (4)	ST110	3083	HKY+F
E. hormaechei	1 (61)	ST66	3118	TVM+F+I
E. hormaechei	2 (8)	ST108	3184	HKY+F+I

* In cases where there were more than one Sequence Type (ST) within a variable length k-mer clusters (VLKCs), the ST with the largest number of isolates was chosen as a reference.

Supplementary Table S3. Quality metrics of complete genomes and draft genome best assemblies used as references for read mapping

Species	ID/Accession	N50 (Kbp)	Largest contig (Kbp)	Length (Mbp)	Mismatches/100kbp	Indels/100kbp	No of contigs	Sequence Type (ST)
K. pneumoniae	3494	198.8	501.3	5.7	737.62	14.03	274	26
K. pneumoniae	3677	203.8	464.8	5.4	710.62	14.58	186	922
E. coli	3341	362.2	1075.5	5.1	2217.69	28.34	117	38
E. coli	3315	269.6	586	4.9	1270.43	18.06	95	162
E. coli	3346	233.5	464.9	4.9	1156.71	15.69	182	410
E. coli	3305	285.4	537.5	5.1	2146.98	27.6	145	69
E. coli	3276	307.1	491.1	5.1	2533.6	28.29	205	12
E. coli	3218	93.5	263.9	5.1	1263.81	18.36	330	401
E. coli	3297	158.6	590.1	5	1264.74	17.71	281	58
K oxytoca	3421	365.3	569.6	6.2	525.38	10.28	210	58
K. oxytoca	3388	301.3	685.7	6.4	3990.79	11.95	139	225_2
K. oxytoca	3390	485.9	1122.1	6.1	3923.46	15.19	98	225_1
K. oxytoca	3433	238.9	864.2	6.3	559.11	9.4	148	36
E. hormachei	3118	441.2	969.2	5	2514.55	40.9	109	66
E. hormachei	3184	263.2	716.2	5.1	1968.7	33.9	121	108
E. cloacae	3098	663.9	938	4.9	3095.98	20.83	59	1125
E. cloacae	3103	493.4	2157.4	4.9	3053.63	33.75	95	78
E. cloacae	3083	745	1155.2	5.2	2683.66	16.98	126	110
K. pneumoniae	CP014762.1	5351509	5351.5	5.4	NA	NA	1	37
K. pneumoniae	CP016813.1	5412960	5413.0	5.4	NA	NA	1	23
K. pneumoniae	CP012745.1	5344576	5344.6	5.3	NA	NA	1	147
K. pneumoniae	CP017934.1	5387681	5387.7	5.4	NA	NA	1	45
K. pneumoniae	CP018447.1	5497872	5497.9	5.5	NA	NA	1	101
K. pneumoniae	CP028391.1	5375449	5375.4	5.4	NA	NA	1	36
K. pneumoniae	CP024542.1	5337491	5337.5	5.3	NA	NA	1	37
K. pneumoniae	CP024515.1	5228900	5228.9	5.2	NA	NA	1	323
K. pneumoniae	CP025143.1	5388622	5388.6	5.4	NA	NA	1	307
K. pneumoniae	CP014123.1	5264538	5264.5	5.3	NA	NA	1	13
K. pneumoniae	CP029388.1	5288751	5288.8	5.3	NA	NA	1	48
K. pneumoniae	CP030172.1	5214209	5214.2	5.2	NA	NA	1	29
K. pneumoniae	CP031810.1	5348492	5348.5	5.3	NA	NA	1	25
K. pneumoniae	CP035531.1	5235159	5235.2	5.2	NA	NA	1	20

Supplementary Table S4. Threshold values for transmission analysis

Species	Methods for calculating thresholds to infer recent transmissions	Substitutions per site per year/ST	Reference	Threshold
Klebsiella pneumoniae	1.Within host diversity of 7 SNPs + (substitution /genome/3 months) x 2<br>2. Pairwise SNP distance that captures 95% cases within 21 SNPs isolated within 90 days	$1 \cdot 9 \times 10^{-6}$ /ST258	(3, 5, 6)	12<br>12
Escherichia coli	1.Within host diversity of 17 SNPs + (substitution /genome/3 months) x 2<br>2. Pairwise SNP distance that captures 95% cases within 21 SNPs isolated within 90 days	$2 \cdot 26 \times 10^{-7}$ /ST73	(5, 7, 8)	18<br>15
Klebsiella oxytoca	Pairwise SNP distance that captures 95% cases within 21 SNPs isolated within 90 days	NA	(5)	17
Enterobacter cloacae	Pairwise SNP distance that captures 95% cases within 21 SNPs isolated within 90 days	NA	(5)	13
Enterobacter. hormaechei	Pairwise SNP distance that captures 95% cases within 21 SNPs isolated within 90 days	NA	(5)	19
Citrobacter freundii	Pairwise SNP distance that captures 95% cases within 21 SNPs isolated within 90 days	NA	(5)	19

Supplementary Table S2 shows the methodologies used in the calculation of threshold values for the transmission analysis. 21 SNPs has previously been reported to discriminate between hospital clones in *K. pneumoniae* over a six month period (4) and here we used the same threshold to derive thresholds for recent (90 days) transmission. Substitution rates were available for *Klebsiella oxytoca* and *Klebsiella cloacae* but not for *Enterobacter hormaechei* or *Citrobacter freundii*. Since within host diversity data was unavailable for these four species, thresholds were calculated based on pairwise SNP distance distributions that captures 95% cases within 21 SNPs isolated within 90 days. NA Not applicable. SNP = nucleotide polymorphism, ST = sequence type.

Supplementary Table S5. Frequency of Sequence Types (STs). Isolates that occurred in less than 5% of each species are designated as minor STs.

K. pneumoniae

ST	Count
Common STs	
258	30
111	21
133	20
512	11
Minor STs	
11	12
323	9
37	9
336	7
20	6
26	6
922	6
23	5
48	5
307	5
14	4
29	4
13	3
15	3
36	3
45	3
147	3
1322	3
1373	3
3514	3
3516	3
25	2
27	2
101	2
240	2
252	2
309	2
834	2
1380	2
2891	2
2974	2
3181	2
3515	2
34	1
113	1
151	1
160	1
188	1
191	1
219	1
250	1
268	1
274	1
392	1
412	1
441	1
465	1
490	1
528	1
551	1
556	1
610	1
661	1
719	1
791	1
857	1
883	1
1307	1
1662	1
2981	1
3517	1
3518	1
3519	1
3520	1
3521	1

E. coli

ST	Count
Common STs	
131	23
38	16
10	14
Minor STs	
410	6
12	5
167	5
162	4
401	4
58	3
69	3
88	3
95	3
127	3
155	3
349	3
405	3
940	3
1049	3
73	2
101	2
141	2
357	2
399	2
617	2
636	2
648	2
1079	2
1193	2
1485	2
1727	2
6958	2
7401	2
23	1
28	1
46	1
57	1
68	1
120	1
135	1
156	1
216	1
295	1
297	1
336	1
348	1
354	1
361	1
367	1
372	1
404	1
409	1
423	1
501	1
506	1
538	1
540	1
542	1
744	1
973	1
1170	1
1236	1
1316	1
1442	1
1611	1
1702	1
1722	1
2015	1
2083	1
2599	1
2622	1
3018	1
3056	1
3221	1
4093	1
4360	1
4959	1
4981	1
5317	1
5778	1
6178	1
7236	1
7400	1
7416	1
7696	1
8818	1
8819	1
8820	1
8822	1

K. oxytoca

ST	Count
Common STs	
225	31
58	18
36	5
Minor STs	
199	4
77	3
34	2
178	2
222	2
223	2
21	1
37	1
40	1
49	1
50	1
88	1
135	1
141	1
172	1
180	1
183	1
184	1
201	1
224	1
228	1
229	1
230	1
231	1

E. hormaechei

ST	Count
Common STs	
66	63
Minor STs	
108	6
1131	2
269	2
1128	1
1130	1
97	1

C. freundii

ST	Count
Common STs	
22	16
166	3
402	2
417	2
Minor STs	
8	1
18	1
62	1
64	1
95	1
98	1
114	1
116	1
169	1
185	1
414	1
415	1
416	1
419	1
421	1
423	1
424	1
427	1
428	1

E. cloacae

ST	Count
Common STs	
1125	5
78	5
110	4
93	4
190	3
Minor STs	
135	2
32	1
45	1
102	1
796	1
1126	1
1127	1
1129	1
1132	1

C. FDAARGOS_156

ST	Count
Common STs	
420	30
Minor STs	
128	1

K. variicola

ST	Count
2978	2
355	1
360	1
697	1
919	1
1478	1
1563	1

C. braakii

ST	Count
110	1
370	1
422	1

K. sp. M5al

ST	Count
186	2
227	1

E. asburiae

ST	Count
24	2

K. sp. LTGPAF-6

ST	Count
85	1

C. farmeri

ST	Count
NA	3

L. adecarboxyla

ST	Count
NA	1

P. stuartii

ST	Count
NA	1

R. ornithinolytic

ST	Count
NA	3

S. marcescens

ST	Count
NA	6

Supplementary Table S6. Characteristics of Enterobacterale isolates. Species that were present at >5% of each species are shown.

Species (n)	No. isolates submitted as carbapenem positive %	No. of STs (and STs with >5% isolates)*	No. of Hospitals with >5% isolates (Hospital Codes)	Carbapenemase genes (and no. of isolates)	No. resistant* to 3 or more other beta – lactams*	No. resistant to 1 or more amniogly – cosides**	No. resistant to tigecycline
Klebsiella pneumoniae (243)	32·6	69 [ST258/ST512 (16·8%), ST111 (8·6%), ST133 (8·2%]	4 [1(19·0%), 10 (5·8%), 27 (24·8%), 28 (17·4%)	bla_{KPC-2} (53), bla_{KPC-3} (16), bla_{OXA-48} (125), $bla_{OXA-like}$ (2), $bla_{NDM-like}$ (45), $bla_{NDM-like}$ + bla_{KPC-2} (1), bla_{OXA-48} + $bla_{NDM-like}$ (1)	240/243 tested	147/243 tested	88/235 tested
Escherichia coli (191)	25·6	88 [ST131 (12·0%), ST38 (8·3%), ST10 (7·3%)]	4 [1 (43·5%), 26 (5·8%), 27 (11·0%), 28 (10·5%)]	bla_{KPC-2} (14) bla_{OXA-48} (148), $bla_{OXA-like}$ (13), bla_{NDM-2} (17), bla_{OXA-48} + $bla_{NDM-like}$,(1), $bla_{VIM-like}$ (2)	188/189 tested	47/89 tested	9/177 tested
Klebsiella oxytoca (87)	11·7	27 [ST36 (5·7%) ST58 (20·7%), ST225 (35·6%)	3 [1 (43·7%), 26 (8·0%), 27 (35·6%)]	bla_{KPC-2} (33), bla_{OXA-48} (43), $bla_{VIM-like}$ (6), $bla_{IMP-like}$ (5)	84/84 tested	17/87 tested	16/73 tested
Enterobacter hormaechei (76)	10·2	7 [ST66 (82·9%), ST108 (7·9%)]	3 [1(72·35), 26 (14·5%), 27 (6·6%)	bla_{KPC-2} (5), bla_{OXA-48} (65), $bla_{OXA-like}$ (1), $bla_{VIM-like}$ (5)	75/75 tested	72/76 tested	23/64 tested
Citrobacter freundii (42)	5·6	23 [ST22 (38·0%), ST166 (7·1%), ST402 (5·0%), ST417 (5·0%)	[1(52·4%) 26 (7·1%), 27 (28·5%)	bla_{KPC-2} (12), bla_{OXA-48} (28), $bla_{OXA-like}$ (1), bla_{NDM-2} (1)	40/42 tested	28/42 tested	9/33 tested
Citrobacter sp. FDAARGOS _156 (31)	4·2	2 [ST420 (96·8%)]	2 [1 (9·7%), 27	bla_{KPC-2} (30), bla_{OXA-48} (1)	31/31 tested	30/31	20/28 tested
Enterobacter cloacae (31)	4·2	14 [ST78 (16·1%), ST93 (12·9%) ST110 (12·9%), ST135 (6·5%), ST190 (9·7%), ST1125 (16·1%)]	9 [1 (9·7%), 2 (6·5%), 4 (6·5%), 12 (6·5%), 13 (13·0%), 22 (6·5%), 23 (9·7%), 26 (13·0%), 28 (16·1%)]	bla_{OXA-48} (20), $bla_{IMP-like}$ (4), $bla_{IMI-like}$ (1), bla_{NDM-2} (2), $bla_{VIM-like}$ (4)	30/30 tested	20/31 tested	10/25 tested

* Susceptibility data was available for at least 7/9 beta-lactams tested.

** Susceptibility data was available for at least 2/3 aminoglycosides teste

Supplementary Table S7. Details of all carbapenemase producing Enterobacterales isolates sequenced

Sample Name	Accession No.	VLKC	Isolation Date	Patient ID	Sequence Type	Species	Source	Hospital Code	CPE	AMP	AMC	CPD	CPDCV	CTX	ERT	FOX	MERO	TZP	GENT	AMK	TOB	TIGE
3000	ERR3143140	-	10/12/2015	IRE193	370	*C. braakii*	Rectal	27	blaKPC_2	R	R	R	R	R	R	R	R	R	I	-	-	-
3001	ERR3143119	-	25/10/2016	IRE345	110	*C. braakii*	Faeces	1	blaOXA_48	R	R	R	R	I	R	R	I	R	S	S	S	S
3002	ERR3143168	-	16/11/2016	IRE449	422	*C. braakii*	NA	1	blaOXA_48	R	R	R	R	R	R	R	S	R	S	S	S	S
3005	ERR3143202	-	13/11/2013	IRE714	NA	*C. farmeri*	Rectal	3	blaOXA_162	-	-	-	-	-	R	-	S	-	-	S	S	-
3003	ERR3143176	-	16/03/2017	IRE581	NA	*C. farmeri*	Rectal	27	blaKPC_2	-	-	-	-	-	R	-	R	-	S	S	R	S
3004	ERR3143209	-	19/01/2017	IRE513	NA	*C. farmeri*	Rectal	27	blaKPC_2	-	-	-	-	-	R	-	R	-	S	S	S	-
3007	ERR3143139	1	08/12/2015	IRE189	22	*C. freundii*	Urine	27	blaKPC_2	R	R	R	R	R	R	R	R	R	R	-	R	R
3031	ERR3143178	16	30/03/2017	IRE594	417	*C. freundii*	Rectal	1	blaOXA_48	R	R	R	R	R	R	I	S	R	S	S	S	S
3012	ERR3143180	17	06/04/2017	IRE605	421	*C. freundii*	NA	27	blaKPC_2	-	-	-	-	-	R	-	R	-	-	-	R	S
3017	ERR3143212	1	21/12/2013	IRE719	428	*C. freundii*	Tissue	27	blaKPC_2	R	R	R	R	R	R	R	I	R	R	-	R	S
3016	ERR3143127	1	07/08/2015	IRE134	22	*C. freundii*	Rectal	27	blaKPC_2	R	R	R	R	R	R	R	R	R	R	S	R	-
3041	ERR3143150	12	08/07/2016	IRE285	415	*C. freundii*	Toe	2	blaOXA_48	R	R	R	R	R	R	R	I	R	S	S	R	S
3039	ERR3143208	1	29/09/2016	IRE371	22	*C. freundii*	Environ	1	blaOXA_48	R	R	R	R	R	R	I	S	R	R	S	R	R
3029	ERR3143169	1	07/12/2016	IRE477	22	*C. freundii*	Rectal	1	blaOXA_48	R	R	R	R	R	R	R	S	R	R	S	R	S
3036	ERR3143194	21	13/01/2017	IRE503	416	*C. freundii*	Rectal	1	blaOXA_48	R	R	R	R	R	R	R	S	R	S	S	S	S
3027	ERR3143165	2	18/10/2016	IRE407	166	*C. freundii*	Faeces	1	blaOXA_48	R	R	R	R	R	R	R	S	R	R	S	R	S
3038	ERR3143201	22	24/05/2017	IRE639	414	*C. freundii*	Wound	1	blaOXA_48	-	-	-	-	R	R	R	S	R	R	I	R	-
3042	ERR3143155	1	22/07/2016	IRE294	22	*C. freundii*	Urine	22	blaOXA_48	R	R	R	R	R	R	R	S	R	R	S	R	S
3045	ERR3143130	6	27/01/2017	IRE524	98	*C. freundii*	Rectal	26	blaOXA_181	R	R	R	R	R	S	R	S	R	I	S	R	S
3010	ERR3143148	1	24/05/2016	IRE265	22	*C. freundii*	Rectal	27	blaKPC_2	R	R	R	R	R	R	R	R	R	R	S	R	R
3025	ERR3143163	3	11/10/2016	IRE389	402	*C. freundii*	Faeces	1	blaOXA_48	R	R	R	R	R	R	I	I	R	R	S	R	S
3024	ERR3143162	1	11/10/2016	IRE387	22	*C. freundii*	NA	1	blaOXA_48	R	R	R	R	R	R	R	S	R	R	S	R	S
3032	ERR3143182	1	27/04/2017	IRE620	22	*C. freundii*	Urine	1	blaOXA_48	-	-	-	-	I	R	R	R	R	R	I	R	-
3020	ERR3143132	8	04/04/2017	IRE599	62	*C. freundii*	Rectal	1	blaOXA_48	-	-	-	-	R	R	R	S	R	R	S	R	S
3008	ERR3143141	1	18/01/2016	IRE203	22	*C. freundii*	Rectal	27	blaKPC_2	R	R	R	R	R	R	R	R	R	R	S	-	-
3018	ERR3143133	9	15/11/2012	EM678	64	*C. freundii*	Faeces	18	blaNDM_1	R	R	R	R	R	R	R	R	R	R	-	R	R
3028	ERR3143123	13	12/09/2016	IRE320	114	*C. freundii*	NA	1	blaOXA_48	R	R	R	R	R	R	I	S	S	S	S	S	S
3023	ERR3143122	2	08/09/2016	IRE325	166	*C. freundii*	Faeces	1	blaOXA_48	R	R	R	R	R	R	R	S	R	S	S	S	R
3047	ERR3143172	15	07/02/2017	IRE536	8	*C. freundii*	Rectal	28	blaOXA_48	R	R	R	R	R	R	R	I	I	S	R	R	S
3033	ERR3143183	1	27/04/2017	IRE622	22	*C. freundii*	Rectal	1	blaOXA_48	-	-	-	-	R	R	S	S	R	R	S	R	-
3015	ERR3143206	1	19/07/2016	IRE292	22	*C. freundii*	Rectal	27	blaKPC_2	R	R	R	R	R	R	R	R	R	R	S	R	R
3011	ERR3143170	1	08/12/2016	IRE479	22	*C. freundii*	Rectal	27	blaKPC_2	R	R	R	R	R	R	R	R	R	R	S	R	R
3026	ERR3143164	3	12/10/2016	IRE390	402	*C. freundii*	Urine	1	blaOXA_48	R	R	R	R	R	R	R	I	R	S	S	S	S
3034	ERR3143192	20	25/10/2016	IRE415	95	*C. freundii*	Faeces	1	blaOXA_48	R	R	R	R	R	R	I	S	R	S	S	S	S
3013	ERR3143189	1	20/04/2015	IRE95	22	*C. freundii*	Rectal	27	blaKPC_2	R	R	R	R	R	R	R	R	R	R	-	R	S
3035	ERR3143193	1	13/12/2016	IRE468	22	*C. freundii*	Envirom	1	blaOXA_48	R	R	R	R	R	R	R	S	R	R	S	R	-
3040	ERR3143211	23	26/04/2017	IRE616	417	*C. freundii*	Rectal	1	blaOXA_48	-	-	-	-	I	R	R	R	R	S	S	S	-
3009	ERR3143145	1	15/03/2016	IRE229	22	*C. freundii*	Rectal	27	blaKPC_2	R	R	R	R	R	R	R	R	R	R	S	R	R
3044	ERR3143128	4	27/09/2016	IRE366	423	*C. freundii*	Rectal	26	blaOXA_48	R	R	R	R	R	R	R	R	R	S	S	S	S
3014	ERR3143197	1	12/05/2015	IRE100	22	*C. freundii*	Rectal	27	blaKPC_2	R	R	R	R	R	R	R	R	R	R	-	R	R
3030	ERR3143171	14	16/12/2016	IRE486	169	*C. freundii*	Rectal	1	blaOXA_48	R	R	R	R	R	R	I	I	R	R	S	R	S
3037	ERR3143196	2	04/04/2017	IRE600	166	*C. freundii*	Toliet R	1	blaOXA_48	-	-	-	-	R	R	R	S	R	S	S	S	-
3019	ERR3143131	7	31/01/2017	IRE529	424	*C. freundii*	Faeces	1	blaOXA_48	R	R	R	R	R	R	I	S	R	S	S	S	S
3046	ERR3143181	18	18/04/2017	IRE598	18	*C. freundii*	Rectal	26	blaOXA_48	-	-	-	-	R	R	R	S	R	S	S	S	-
3043	ERR3143125	19	08/02/2016	IRE213	427	*C. freundii*	Pus	23	blaOXA_48	R	R	R	R	R	R	R	S	R	S	S	S	S
3022	ERR3143121	11	16/09/2016	IRE339	419	*C. freundii*	Faeces	1	blaOXA_48	R	R	R	R	R	R	R	S	R	S	S	S	S
3021	ERR3143120	10	16/09/2016	IRE341	185	*C. freundii*	Faeces	1	blaOXA_48	R	R	R	R	R	R	I	S	R	S	S	S	S
3006	ERR3143129	5	04/05/2017	IRE625	116	*C. freundii*	Rectal	27	blaKPC_2	-	-	-	-	-	R	-	R	-	-	-	R	S
3065	ERR3143158	-	19/08/2016	IRE312	420	*C. sp. FDAARGOS_156*	Rectal	27	blaKPC_2	R	R	R	R	R	R	R	I	R	S	S	R	S
3073	ERR3143188	-	18/03/2015	IRE86	420	*C. sp. FDAARGOS_156*	Rectal	27	blaKPC_2	R	R	R	R	R	R	R	I	R	R	-	R	R
3077	ERR3143210	-	16/02/2017	IRE553	420	*C. sp. FDAARGOS_156*	Envirom	27	blaKPC_2	R	R	R	R	R	R	R	R	R	R	S	S	R
3068	ERR3143173	-	08/09/2014	IRE54	420	*C. sp. FDAARGOS_156*	Rectal	27	blaKPC_2	R	R	R	R	R	R	R	R	R	R	-	R	S
3057	ERR3143144	-	15/03/2016	IRE228	420	*C. sp. FDAARGOS_156*	Rectal	27	blaKPC_2	R	R	R	R	R	R	R	S	R	R	S	R	R
3067	ERR3143166	-	08/08/2014	IRE42	420	*C. sp. FDAARGOS_156*	Rectal	27	blaKPC_2	R	R	R	R	R	R	R	I	R	R	-	R	R
3055	ERR3143142	-	27/01/2016	IRE208	420	*C. sp. FDAARGOS_156*	Toilet b	27	blaKPC_2	R	R	R	R	R	R	R	I	R	R	S	R	-
3053	ERR3143136	-	01/09/2015	IRE152	420	*C. sp. FDAARGOS_156*	Rectal	27	blaKPC_2	R	R	R	R	R	R	R	R	R	R	-	R	R
3051	ERR3143149	-	25/05/2016	IRE266	420	*C. sp. FDAARGOS_156*	Rectal	25	blaKPC_2	R	R	R	R	R	R	R	R	R	R	S	-	-
3075	ERR3143198	-	26/06/2015	IRE119	420	*C. sp. FDAARGOS_156*	Rectal	27	blaKPC_2	R	R	R	R	R	R	R	R	R	R	-	R	S
3056	ERR3143143	-	27/01/2016	IRE209	420	*C. sp. FDAARGOS_156*	Sink Dr	27	blaKPC_2	R	R	R	R	R	R	R	R	R	S	S	R	R
3061	ERR3143153	-	14/06/2016	IRE277	420	*C. sp. FDAARGOS_156*	Rectal	27	blaKPC_2	R	R	R	R	R	R	R	R	R	S	S	R	R
3049	ERR3143167	-	08/11/2016	IRE435	420	*C. sp. FDAARGOS_156*	Faeces	1	blaKPC_2	R	R	R	R	R	R	R	S	R	R	S	S	S
3048	ERR3143134	-	22/05/2017	IRE637	420	*C. sp. FDAARGOS_156*	Rectal	1	blaKPC_2	R	R	-	-	R	R	R	R	R	S	S	R	S
3063	ERR3143156	-	25/07/2016	IRE295	420	*C. sp. FDAARGOS_156*	Rectal	27	blaKPC_2	R	R	R	R	R	R	R	R	R	R	S	R	R
3054	ERR3143137	-	08/09/2015	IRE155	420	*C. sp. FDAARGOS_156*	Rectal	27	blaKPC_2	R	R	R	R	R	R	R	R	R	R	-	R	R
3078	ERR3143191	-	09/08/2016	IRE305	128	*C. sp. FDAARGOS_156*	Faeces	1	blaOXA_48	R	R	R	R	I	S	R	S	R	S	S	S	S
3069	ERR3143174	-	16/02/2017	IRE554	420	*C. sp. FDAARGOS_156*	Rectal	27	blaKPC_2	R	R	R	R	R	R	R	R	R	R	S	R	-
3059	ERR3143151	-	28/06/2016	IRE282	420	*C. sp. FDAARGOS_156*	Rectal	27	blaKPC_2	R	R	R	R	R	R	R	R	R	S	S	R	R
3060	ERR3143152	-	28/06/2016	IRE281	420	*C. sp. FDAARGOS_156*	Rectal	27	blaKPC_2	R	R	R	R	R	R	R	R	R	R	R	R	R
3074	ERR3143195	-	30/01/2017	IRE527	420	*C. sp. FDAARGOS_156*	Rectal	27	blaKPC_2	R	R	R	R	R	R	R	I	R	R	S	R	R
3072	ERR3143187	-	06/06/2013	IRE696	420	*C. sp. FDAARGOS_156*	Rectal	27	blaKPC_2	R	R	R	R	R	R	R	R	R	S	-	R	R

3066	ERR3143159	-	14/09/2016	IRE337	420	*C. sp. FDAARGOS_156*	Rectal	27	blaKPC_2	R	R	R	R	R	R	R	R	R	R	S	R	R
3076	ERR3143204	-	17/08/2015	IRE139	420	*C. sp. FDAARGOS_156*	Rectal	27	blaKPC_2	R	R	R	R	R	R	R	R	R	R	-	R	R
3050	ERR3143126	-	02/12/2014	IRE18	420	*C. sp. FDAARGOS_156*	Rectal	23	blaKPC_2	R	R	R	R	R	R	R	R	R	S	-	R	R
3052	ERR3143135	-	31/08/2015	IRE150	420	*C. sp. FDAARGOS_156*	Rectal	27	blaKPC_2	R	R	R	R	R	R	R	R	R	R	-	R	R
3064	ERR3143157	-	04/08/2016	IRE303	420	*C. sp. FDAARGOS_156*	Rectal	27	blaKPC_2	R	R	R	R	R	R	R	R	R	R	S	R	R
3058	ERR3143147	-	21/03/2016	IRE236	420	*C. sp. FDAARGOS_156*	Rectal	27	blaKPC_2	R	R	R	R	R	R	R	R	R	R	S	R	R
3070	ERR3143177	-	22/03/2017	IRE586	420	*C. sp. FDAARGOS_156*	Rectal	27	blaKPC_2	R	R	R	R	R	R	R	R	R	R	S	R	-
3062	ERR3143154	-	24/06/2014	IRE29	420	*C. sp. FDAARGOS_156*	Rectal	27	blaKPC_2	R	R	R	R	R	R	R	R	R	S	-	R	S
3071	ERR3143186	-	06/11/2014	IRE69	420	*C. sp. FDAARGOS_156*	Rectal	27	blaKPC_2	R	R	R	R	R	R	R	R	R	R	-	R	R
3080	ERS8674504	-	20/02/2017	IRE556	24	*E. asburiae*	Environ	27	blaKPC_2	R	R	R	R	R	R	R	R	R	S	S	S	-
3079	ERS8674464	-	11/04/2017	IRE611	24	*E. asburiae*	Rectal	2	blaIMP_4	-	-	-	-	-	R	-	I	-	-	-	R	S
3095	ERS8674501	4	04/01/2017	IRE493	135	*E. cloacae*	Leg	12	blaOXA_48	R	R	R	R	R	R	R	S	R	S	S	S	S
3085	ERS8674507	3	07/04/2017	IRE610	110	*E. cloacae*	Urine	23	blaIMP_4	-	-	-	-	-	R	-	S	-	-	-	R	S
3097	ERR2107334	1	27/03/2017	IRE591	93	*E. cloacae*	Sputum	22	blaOXA_48	R	R	R	R	R	-	R	S	R	R	S	R	S
3109	ERR2124551	6	27/07/2016	IRE297	1132	*E. cloacae*	Left hip	13	blaVIM_1	R	R	R	R	R	S	R	S	R	S	S	R	R
3090	ERS8674442	1	14/07/2016	IRE289	190	*E. cloacae*	HVS	1	blaOXA_48	R	R	R	R	R	R	R	I	R	S	S	R	R
3091	ERS8674463	12	09/02/2017	IRE545	45	*E. cloacae*	Rectal	4	blaOXA_48	R	R	R	R	R	R	R	S	R	S	S	S	S
3106	ERS8952692	1	31/01/2017	IRE532	1125	*E. cloacae*	Rectal	28	blaOXA_48	R	R	R	R	R	R	R	I	R	S	S	S	S
3092	ERS8674465	1	09/02/2017	IRE545	1125	*E. cloacae*	Rectal	4	blaOXA_48	R	R	R	R	R	R	R	S	R	S	S	S	S
3086	ERS8674485	1	20/09/2016	IRE346	93	*E. cloacae*	Urine	3	blaNDM_1	R	R	R	R	R	R	R	R	R	R	R	R	R
3110	ERR2124554	2	06/10/2016	IRE382	78	*E. cloacae*	Urine	13	blaVIM_1	R	R	R	R	R	-	R	S	R	R	S	R	S
3111	ERR2124557	5	22/12/2016	IRE491	796	*E. cloacae*	Rectal	26	blaVIM_1	R	R	R	R	R	-	R	S	R	S	S	R	S
3105	ERS8674528	1	31/01/2017	IRE531	1125	*E. cloacae*	Rectal	28	blaOXA_48	R	R	R	R	R	R	R	I	R	S	S	S	S
3083	ERS8674518	3	30/09/2013	IRE706	110	*E. cloacae*	Wound	2	blaIMP_4	R	R	R	R	R	R	R	S	S	R	-	-	-
3082	ERS8674469	11	11/09/2015	IRE157	32	*E. cloacae*	Blood	2	blaIMP_4	R	R	R	R	R	R	R	S	R	R	-	R	R
3096	ERS8952744	1	01/09/2016	IRE318	93	*E. cloacae*	Abdomi	22	blaOXA_48	R	R	R	R	R	R	R	S	R	R	S	R	R
3094	ERS8674456	2	13/01/2017	IRE457	78	*E. cloacae*	Urine	11	blaOXA_48	R	R	R	R	R	R	R	R	R	R	S	R	S
3098	ERS8674474	1	04/02/2016	IRE210	190	*E. cloacae*	Urine	23	blaOXA_48	R	R	R	R	R	R	R	S	R	S	S	S	R
3104	ERS8674527	1	27/01/2017	IRE525	1125	*E. cloacae*	Sputum	28	blaOXA_48	R	R	R	R	R	R	R	I	R	S	S	S	S
3099	ERS8674435	10	12/12/2016	IRE482	1126	*E. cloacae*	Urine	23	blaOXA_48	R	R	R	R	R	R	R	S	R	S	S	R	S
3103	ERS8674482	2	18/07/2016	IRE290	78	*E. cloacae*	Stool	28	blaOXA_48	R	R	R	R	R	R	R	S	R	S	S	S	R
3101	ERR2107337	3	27/04/2016	IRE261	110	*E. cloacae*	Rectal	26	blaOXA_48	R	R	R	R	R	R	R	S	R	I	S	R	R
3107	ERS8952695	1	01/06/2017	IRE648	1125	*E. cloacae*	Urine	28	blaOXA_48	-	-	-	-	R	R	R	S	R	S	S	S	-
3089	ERS8674510	1	27/04/2017	IRE621	93	*E. cloacae*	Rectal	1	blaOXA_48	-	-	-	-	R	R	R	S	R	R	S	R	-
3084	ERS8674520	3	02/02/2014	IRE9	110	*E. cloacae*	MSU	12	blaIMP_4	R	R	R	R	R	R	R	S	R	R	-	-	-
3081	ERS8674517	8	04/11/2013	IRE688	1129	*E. cloacae*	Rectal	27	blaIMI_1	R	R	R	R	R	R	S	R	S	S	S	S	-
3093	ERS8674519	2	19/03/2015	IRE88	78	*E. cloacae*	BLC	10	blaOXA_48	R	R	R	R	R	R	R	I	R	S	-	S	R
3087	ERS8674500	9	19/12/2016	IRE487	1127	*E. cloacae*	Blood	13	blaNDM_1	R	R	R	R	R	R	R	S	R	R	R	R	S
3102	ERR2124556	4	09/12/2016	IRE480	135	*E. cloacae*	Rectal	26	blaOXA_48	R	R	R	R	R	-	R	R	R	R	R	R	S
3088	ERS8674496	1	26/10/2016	IRE418	190	*E. cloacae*	Faeces	1	blaOXA_48	R	R	R	R	R	R	R	R	R	S	S	R	S
3108	ERR2107338	7	21/01/2016	IRE204	102	*E. cloacae*	Vas Cat	13	blaVIM_1	R	R	R	R	R	R	R	S	R	R	S	R	R
3100	ERS8674462	2	11/05/2017	IRE629	78	*E. cloacae*	Wound	26	blaOXA_48	-	-	-	-	R	R	R	S	R	R	S	R	-
3112	ERS8674468	-	12/05/2017	IRE629	135	*E. cloacae complex*	Rectal	26	blaVIM_1	-	-	-	-	R	R	R	S	R	S	S	R	-
3135	ERS8674498	1	15/11/2016	IRE448	66	*E. hormaechei*	Faeces	1	blaOXA_48	R	R	R	R	R	R	R	S	R	R	S	R	S
3126	ERS8674487	1	10/10/2016	IRE384	66	*E. hormaechei*	Faeces	1	blaOXA_48	R	R	R	R	R	R	R	S	R	R	S	R	S
3131	ERS8674494	1	18/10/2016	IRE402	66	*E. hormaechei*	Faeces	1	blaOXA_48	R	R	R	R	R	R	R	S	R	R	S	R	S
3132	ERS8674493	1	18/10/2016	IRE403	66	*E. hormaechei*	Faeces	1	blaOXA_48	R	R	R	R	R	R	R	S	R	R	S	R	S
3153	ERS8674444	1	19/09/2016	IRE345	66	*E. hormaechei*	Faeces	1	blaOXA_48	R	R	R	R	R	R	R	I	R	R	R	R	R
3121	ERS8674477	1	14/03/2016	IRE227	66	*E. hormaechei*	Urine	1	blaOXA_48	R	R	R	R	R	R	R	S	R	R	S	R	R
3172	ERS8952743	5	27/07/2016	IRE291	66	*E. hormaechei*	Sputum	1	blaOXA_48	R	R	R	R	R	S	R	S	R	R	S	R	S
3163	ERS8674457	1	22/11/2016	IRE456	66	*E. hormaechei*	Faeces	1	blaOXA_48	R	R	R	R	R	R	R	S	R	I	I	R	R
3170	ERS8952804	1	04/10/2016	IRE376	66	*E. hormaechei*	Faeces	1	blaOXA_48	R	R	R	R	R	R	R	S	R	R	S	R	S
3182	ERR2124553	1	02/09/2016	IRE323	66	*E. hormaechei*	Rectal	26	blaOXA_48	R	R	R	R	R	R	R	S	R	R	S	R	R
3152	ERS8674443	1	16/08/2016	IRE309	66	*E. hormaechei*	Faeces	1	blaOXA_48	R	R	R	R	R	R	R	S	R	R	S	R	R
3125	ERS8674486	8	21/09/2016	IRE351	66	*E. hormaechei*	Faeces	1	blaOXA_48	R	R	R	R	R	S	R	S	R	R	S	R	S
3123	ERS8674478	1	04/05/2016	IRE263	66	*E. hormaechei*	Urine	1	blaOXA_48	R	R	R	R	R	R	R	S	R	R	S	R	R
3179	ERR2107336	1	02/06/2016	IRE273	66	*E. hormaechei*	Rectal	26	blaOXA_48	R	R	R	R	R	R	R	S	R	R	S	R	R
3158	ERS8674449	1	11/10/2016	IRE385	66	*E. hormaechei*	Faeces	1	blaOXA_48	R	R	R	R	R	R	R	S	R	R	S	R	S
3118	ERS8674466	1	12/05/2017	IRE630	66	*E. hormaechei*	Rectal	1	blaOXA_48	-	-	-	-	R	R	R	I	R	R	S	R	-
3155	ERS8674447	1	22/09/2016	IRE354	66	*E. hormaechei*	Faeces	1	blaOXA_48	R	R	R	R	R	R	R	S	R	R	S	R	S
3174	ERS8674445	2	15/03/2017	IRE579	108	*E. hormaechei*	Urine	3	blaOXA_48	R	R	R	R	R	R	R	S	R	R	S	R	S
3178	ERR2107332	1	04/04/2017	IRE598	66	*E. hormaechei*	Tissue	26	blaOXA_48	R	R	R	R	R	-	R	S	R	R	S	-	S
3145	ERS8674524	1	21/07/2015	IRE130	66	*E. hormaechei*	Faeces	1	blaOXA_48	R	R	R	R	R	R	R	I	R	R	-	R	R
3146	ERS8674526	1	08/09/2016	IRE329	66	*E. hormaechei*	Faeces	1	blaOXA_48	R	R	R	R	R	R	R	S	R	R	S	R	R
3159	ERS8674453	1	18/10/2016	IRE404	66	*E. hormaechei*	Faeces	1	blaOXA_48	R	R	R	R	R	R	R	S	R	R	S	R	S
3181	ERR2124552	1	15/08/2016	IRE308	66	*E. hormaechei*	Sputum	26	blaOXA_48	R	R	R	R	R	R	R	S	R	R	S	R	R
3169	ERS8952742	1	22/09/2016	IRE355	66	*E. hormaechei*	Rectal	1	blaOXA_48	R	R	R	R	R	R	R	S	R	R	S	R	S
3168	ERS8952739	1	19/09/2016	IRE343	66	*E. hormaechei*	Faeces	1	blaOXA_48	R	R	R	R	R	R	R	S	R	R	S	R	S
3162	ERS8674455	1	22/11/2016	IRE455	66	*E. hormaechei*	Faeces	1	blaOXA_48	R	R	R	R	R	R	R	S	R	S	S	R	S
3122	ERS8674479	1	22/04/2016	IRE260	66	*E. hormaechei*	Pleural	1	blaOXA_48	R	R	R	R	R	R	R	S	R	R	S	R	R
3165	ERS8674459	4	06/12/2016	IRE475	1131	*E. hormaechei*	Rectal	1	blaOXA_48	R	R	R	R	R	R	R	S	R	S	S	S	S
3188	ERR2124559	2	11/01/2017	IRE502	108	*E. hormaechei*	Rectal	26	blaVIM_1	R	R	R	R	R	-	R	I	R	I	S	R	S
3142	ERS8674511	1	04/05/2017	IRE623	66	*E. hormaechei*	Urine	1	blaOXA_48	-	-	-	-	R	R	R	I	R	R	S	R	-

3119	ERS8674472	1	21/12/2015	IRE196	66	*E. hormaechei*	Faeces	1	blaOXA_48	R	R	R	R	R	R	R	S	R	R	-	R	S
3113	ERS8674480	2	30/05/2016	IRE269	108	*E. hormaechei*	Rectal	27	blaKPC_2	R	R	R	R	R	R	R	R	R	S	S	-	-
3124	ERS8674484	1	14/09/2016	IRE334	66	*E. hormaechei*	Blood	1	blaOXA_48	R	R	R	R	R	R	R	S	R	R	S	R	S
3166	ERS8674461	1	03/01/2017	IRE492	66	*E. hormaechei*	Faeces	1	blaOXA_48	R	R	R	R	R	R	R	S	R	R	S	R	S
3175	ERS8674467	1	12/05/2017	IRE630	66	*E. hormaechei*	Rectal	23	blaOXA_48	-	-	-	-	R	R	R	S	R	R	S	R	-
3149	ERS8674438	1	27/01/2016	IRE194	66	*E. hormaechei*	Sputum	1	blaOXA_48	R	R	R	R	R	R	R	R	R	R	S	R	R
3128	ERS8674492	1	18/10/2016	IRE398	66	*E. hormaechei*	Faeces	1	blaOXA_48	R	R	R	R	R	R	R	S	R	R	S	R	S
3151	ERS8674441	1	31/05/2016	IRE272	66	*E. hormaechei*	Femoral	1	blaOXA_48	R	R	R	R	R	R	R	S	R	R	S	R	S
3147	ERS8674525	1	22/11/2016	IRE454	66	*E. hormaechei*	Faeces	1	blaOXA_48	R	R	R	R	R	R	R	S	R	R	S	R	S
3115	ERS8674515	3	08/06/2017	IRE653	269	*E. hormaechei*	Rectal	27	blaKPC_2	-	-	-	-	R	R	R	I	R	S	S	R	-
3186	ERR2107335	2	24/02/2017	IRE560	108	*E. hormaechei*	Rectal	26	blaVIM_1	R	R	R	R	R	-	R	R	R	I	S	R	S
3114	ERS8674502	3	06/01/2017	IRE494	1128	*E. hormaechei*	Rectal	27	blaKPC_2	R	R	R	R	R	R	R	R	R	S	S	R	-
3167	ERS8952745	1	05/09/2016	IRE324	66	*E. hormaechei*	Faeces	1	blaOXA_48	R	R	R	R	R	R	R	S	R	R	S	R	S
3137	ERS8674503	1	17/02/2017	IRE555	66	*E. hormaechei*	Urine	1	blaOXA_48	R	R	R	R	R	R	R	S	R	R	S	R	S
3183	ERR2124555	2	07/12/2016	IRE476	108	*E. hormaechei*	Rectal	26	blaOXA_181	R	R	R	R	R	-	R	S	R	R	S	R	S
3130	ERS8674491	1	18/10/2016	IRE400	66	*E. hormaechei*	Faeces	1	blaOXA_48	R	R	R	R	R	R	R	I	R	R	S	R	S
3141	ERS8674508	1	20/04/2017	IRE616	66	*E. hormaechei*	Rectal	1	blaOXA_48	-	-	-	-	R	R	R	I	R	R	S	R	-
3154	ERS8674446	1	20/09/2016	IRE347	66	*E. hormaechei*	Faeces	1	blaOXA_48	R	R	R	R	R	S	R	S	R	R	S	R	S
3164	ERS8674458	1	30/11/2016	IRE468	66	*E. hormaechei*	Environ	1	blaOXA_48	R	R	R	R	R	R	R	S	R	S	S	R	S
3138	ERS8674506	1	22/02/2017	IRE558	66	*E. hormaechei*	Rectal	1	blaOXA_48	R	R	R	R	R	R	R	S	R	R	S	R	S
3140	ERS8674509	1	20/04/2017	IRE615	66	*E. hormaechei*	Rectal	1	blaOXA_48	-	-	-	-	R	R	R	I	R	R	S	R	-
3187	ERR2124558	4	09/01/2017	IRE495	1131	*E. hormaechei*	Urine	26	blaVIM_1	R	R	R	R	R	-	R	I	R	S	S	R	S
3117	ERS8952694	3	30/05/2017	IRE644	269	*E. hormaechei*	Rectal	27	blaKPC_2	-	-	-	-	-	R	-	S	-	-	-	S	S
3150	ERS8674440	1	24/03/2016	IRE241	66	*E. hormaechei*	Urine	1	blaOXA_48	R	R	R	R	R	R	R	S	R	R	S	R	R
3133	ERS8674495	1	18/10/2016	IRE405	66	*E. hormaechei*	Faeces	1	blaOXA_48	R	R	R	R	R	R	R	S	R	S	S	R	S
3136	ERS8674499	1	21/11/2016	IRE453	66	*E. hormaechei*	Faeces	1	blaOXA_48	R	R	R	R	R	R	S	S	R	R	R	R	S
3139	ERS8674505	1	07/04/2017	IRE669	66	*E. hormaechei*	Rectal	1	blaOXA_48	-	-	-	-	R	R	R	S	R	R	S	R	-
3144	ERS8674512	1	18/05/2017	IRE635	66	*E. hormaechei*	Rectal	1	blaOXA_48	-	-	-	-	R	R	R	S	R	R	S	R	-
3180	ERR2124550	1	08/07/2016	IRE284	66	*E. hormaechei*	Rectal	26	blaOXA_48	R	R	R	R	R	R	R	S	R	R	S	R	R
3160	ERS8674452	1	25/10/2016	IRE416	66	*E. hormaechei*	Faeces	1	blaOXA_48	R	R	R	R	R	R	R	S	R	R	S	R	S
3143	ERS8674514	1	10/05/2017	IRE503	66	*E. hormaechei*	Rectal	1	blaOXA_48	-	-	-	-	R	R	R	I	R	R	S	R	-
3177	ERS8674439	1	19/02/2016	IRE213	66	*E. hormaechei*	Abscess	23	blaOXA_48	R	R	R	R	R	R	R	R	R	R	S	R	R
3134	ERS8674497	1	14/11/2016	IRE443	66	*E. hormaechei*	Faeces	1	blaOXA_48	R	R	R	R	R	R	R	S	R	R	S	R	S
3156	ERS8674448	1	27/09/2016	IRE360	1130	*E. hormaechei*	Faeces	1	blaOXA_48	R	R	R	R	R	S	R	S	R	S	S	R	S
3157	ERS8674450	1	27/09/2016	IRE364	66	*E. hormaechei*	Rectal	1	blaOXA_48	R	R	R	R	R	S	R	S	R	R	S	R	S
3116	ERS8674460	1	19/08/2014	IRE48	66	*E. hormaechei*	Rectal	27	blaKPC_2	R	R	R	R	R	R	R	I	R	S	-	S	R
3173	ERS8674483	1	27/06/2016	IRE280	66	*E. hormaechei*	Wound	2	blaOXA_48	R	R	R	R	R	R	R	S	R	R	I	R	R
3129	ERS8674490	1	18/10/2016	IRE399	66	*E. hormaechei*	Faeces	1	blaOXA_48	R	R	R	R	R	R	R	S	R	R	S	R	S
3171	ERS8952828	1	12/10/2016	IRE392	66	*E. hormaechei*	Faeces	1	blaOXA_48	R	R	R	R	R	R	R	R	R	S	S	R	R
3184	ERS8674470	2	18/09/2015	IRE162	108	*E. hormaechei*	Rectal	26	blaVIM_1	R	R	R	R	R	R	R	R	R	R	-	R	R
3127	ERS8674489	1	17/10/2016	IRE396	66	*E. hormaechei*	Faeces	1	blaOXA_48	R	R	R	R	R	R	R	S	R	R	S	R	S
3176	ERS8674476	1	01/03/2016	IRE220	66	*E. hormaechei*	Urine	23	blaOXA_48	R	R	R	R	R	R	R	R	R	R	S	R	R
3161	ERS8674454	1	03/11/2016	IRE422	66	*E. hormaechei*	Faeces	1	blaOXA_48	R	R	R	R	R	R	R	I	R	R	S	R	R
3148	ERS8674437	6	17/12/2015	IRE194	66	*E. hormaechei*	Sputum	1	blaOXA_48	R	R	R	R	R	R	R	I	R	R	-	R	R
3120	ERS8674475	1	10/02/2016	IRE214	66	*E. hormaechei*	Abdomi	1	blaOXA_48	R	R	R	R	R	R	R	S	R	S	S	R	R
3185	ERS8674513	7	29/05/2017	IRE642	97	*E. hormaechei*	SNS	26	blaVIM_1	-	-	-	-	R	R	R	S	R	S	S	R	-
3330	ERR7221554	1	21/10/2014	IRE66	10	*E. coli*	Faeces	18	blaOXA_181	R	R	R	R	R	R	R	S	R	S	-	S	S
3199	ERR7221545	13	12/04/2017	IRE583	95	*E. coli*	Rectal	27	blaKPC_2	R	R	-	-	R	R	S	R	R	S	S	R	R
3240	ERR7221521	53	27/09/2016	IRE359	336	*E. coli*	Faeces	1	blaOXA_48	R	R	S	I	I	R	S	S	R	S	S	S	S
3360	ERR7221505	14	23/01/2015	IRE83	636	*E. coli*	Urine	28	blaOXA_48	R	R	R	R	R	R	R	S	R	S	-	R	S
3229	ERR7221486	27	25/11/2016	IRE460	1316	*E. coli*	Faeces	1	blaOXA_48	R	R	S	S	S	R	S	S	R	S	S	S	S
3265	ERR7221541	1	13/03/2017	IRE576	10	*E. coli*	Rectal	1	blaOXA_48	R	R	S	I	I	R	S	S	R	S	S	S	S
3287	ERR2124228	39	07/09/2016	IRE328	3056	*E. coli*	Faeces	1	blaOXA_48	R	R	R	R	R	R	R	R	R	S	S	S	S
3218	ERR1990254	9	28/09/2016	IRE369	401	*E. coli*	Faeces	18	blaNDM_1	R	R	R	R	R	R	R	S	R	R	R	R	S
3328	ERR7221567	4	28/11/2016	IRE462	3221	*E. coli*	Urine	16	blaOXA_181	R	R	R	R	R	R	R	S	R	R	S	R	S
3282	ERR1990253	23	28/09/2016	IRE368	1485	*E. coli*	Faeces	1	blaOXA_48	R	R	S	I	I	R	S	S	R	S	S	S	S
3326	ERR2124240	36	24/10/2016	IRE414	1611	*E. coli*	Urine	15	blaOXA_48	R	R	R	R	I	-	S	S	R	S	S	S	S
3276	ERR1987537	7	21/09/2016	IRE352	12	*E. coli*	Bile	1	blaOXA_48	R	R	S	S	S	S	S	S	R	S	S	S	S
3277	ERR1987539	30	22/09/2016	IRE354	1727	*E. coli*	Faeces	1	blaOXA_48	R	R	R	R	R	R	S	S	R	S	S	S	S
3266	ERR1837598	32	19/05/2016	IRE264	7236	*E. coli*	Faeces	1	blaOXA_48	R	R	S	S	S	R	S	I	R	S	S	S	S
3191	ERR7221467	63	03/09/2015	IRE144	1170	*E. coli*	Rectal	27	blaKPC_2	R	R	R	R	R	R	R	R	R	S	-	S	S
3320	ERR7221568	3	25/01/2017	IRE521	38	*E. coli*	Urine	12	blaOXA_48	R	R	R	R	R	R	R	S	R	R	S	R	S
3216	ERR7221470	62	15/10/2015	IRE169	2083	*E. coli*	Rectal	14	blaNDM_1	R	R	R	R	R	R	R	R	R	R	-	R	S
3270	ERR1837602	1	28/07/2016	IRE298	10	*E. coli*	Faeces	1	blaOXA_48	R	R	S	I	S	R	S	R	R	S	S	S	S
3249	ERR7221562	9	12/09/2016	IRE320	401	*E. coli*	Faeces	1	blaOXA_48	R	R	S	I	S	S	S	S	R	S	S	S	S
3303	ERR7221557	3	11/11/2014	IRE71	38	*E. coli*	Rectal	3	blaOXA_48	R	R	R	R	R	R	R	S	R	S	-	S	S
3211	ERR7221564	15	26/11/2015	IRE184	405	*E. coli*	Blood	33	blaNDM_5	R	R	R	R	R	R	R	R	R	R	-	-	-
3209	ERR7221463	1	09/06/2015	IRE113	617	*E. coli*	Faeces	18	blaNDM_5	R	R	R	R	R	R	R	R	R	R	-	R	S
3355	ERR7221584	4	23/09/2014	IRE61	88	*E. coli*	Urine	27	blaOXA_48	R	R	S	I	S	R	S	S	R	S	-	S	S
3239	ERR7221520	4	28/10/2016	IRE420	423	*E. coli*	Faeces	1	blaOXA_48	R	R	S	I	I	R	S	S	R	S	S	S	S
3359	ERR7221490	26	31/01/2017	IRE534	6958	*E. coli*	Rectal	28	blaOXA_48	R	R	R	R	R	R	S	I	R	S	S	S	S
3246	ERR7221544	24	07/04/2017	IRE608	73	*E. coli*	Rectal	1	blaOXA_48	R	R	-	-	S	R	S	I	R	S	S	S	-
3289	ERR2124231	8	04/10/2016	IRE377	7401	*E. coli*	Faeces	1	blaOXA_48	R	R	S	S	S	R	S	S	R	S	S	S	S

3353	ERR7221524	2	26/01/2016	IRE207	131	*E. coli*	Urine	27	blaOXA_48	R	R	R	R	R	S	S	S	R	S	S	S	S
3215	ERR2124241	35	02/12/2016	IRE246	2015	*E. coli*	Urine	10	blaNDM_1	R	R	R	R	R	-	R	S	R	S	S	S	S
3272	ERR1987533	8	04/10/2016	IRE375	7401	*E. coli*	Rectal	1	blaOXA_48	R	R	S	S	S	R	S	S	R	S	S	S	S
3358	ERR7221472	2	17/11/2015	IRE181	506	*E. coli*	Rectal	28	blaOXA_48	R	R	S	I	I	S	R	S	R	R	-	R	S
3204	ERR7221583	1	26/09/2014	IRE58	167	*E. coli*	Rectal	2	blaNDM_5	R	R	R	R	R	R	R	R	R	R	-	S	S
3377	ERR2124250	2	25/01/2017	IRE519	131	*E. coli*	Rectal	28	blaOXA_48	R	R	R	R	R	-	S	S	R	S	S	S	S
3356	ERR1981378	2	01/09/2016	IRE322	131	*E. coli*	Urine	27	blaOXA_48	R	R	R	R	R	R	S	S	R	R	S	R	S
3340	ERR7221471	61	20/10/2015	IRE172	542	*E. coli*	Sputum	25	blaOXA_48	R	R	S	S	S	R	S	S	R	S	-	S	S
3364	ERR7221465	51	23/06/2015	IRE118	5778	*E. coli*	Blood	28	blaOXA_48	R	R	R	R	R	R	S	S	R	S	-	S	S
3361	ERR7221506	3	04/02/2015	IRE83	38	*E. coli*	Urine	28	blaOXA_48	R	R	R	R	R	R	R	S	R	S	-	S	S
3223	ERR7221480	29	02/12/2016	IRE422	5317	*E. coli*	Rectal	1	blaOXA_48	R	R	R	R	R	R	R	I	R	S	S	S	S
3322	ERR1987530	1	09/09/2016	IRE326	10	*E. coli*	Rectal	12	blaOXA_48	R	R	S	S	I	R	S	S	R	S	S	S	S
3256	ERR7221577	2	07/09/2016	IRE327	131	*E. coli*	Faeces	1	blaOXA_48	R	R	R	R	I	R	R	I	R	S	S	S	S
3267	ERR1837599	18	07/06/2016	IRE241	101	*E. coli*	Bile	1	blaOXA_48	R	R	S	I	I	R	S	S	R	S	S	S	S
3232	ERR7221494	9	30/03/2017	IRE594	401	*E. coli*	Rectal	1	blaOXA_48	R	R	S	I	S	S	S	S	R	S	S	S	S
3304	ERR7221595	21	11/11/2013	IRE714	348	*E. coli*	Rectal	3	blaOXA_162	R	R	R	R	R	R	R	I	R	S	-	S	S
3250	ERR7221566	50	21/12/2015	IRE197	501	*E. coli*	Faeces	1	blaOXA_48	R	R	S	I	R	R	S	I	R	S	-	S	S
3220	ERR7221572	20	19/08/2014	IRE48	540	*E. coli*	Rectal	27	blaNDM_1	R	R	R	R	R	R	R	R	R	S	-	R	S
3194	ERR7221492	9	16/03/2017	IRE582	401	*E. coli*	Rectal	27	blaKPC_2	R	R	R	R	R	S	R	S	R	S	S	R	S
3262	ERR7221602	28	09/11/2016	IRE437	399	*E. coli*	Faeces	1	blaOXA_48	R	R	R	R	I	R	R	I	R	S	S	S	S
3371	ERR7221589	12	02/12/2016	IRE469	940	*E. coli*	Rectal	28	blaOXA_181	R	R	R	R	S	R	S	S	R	S	S	S	S
3352	ERR7221504	25	14/01/2015	IRE81	1079	*E. coli*	Rectal	27	blaOXA_48	R	R	S	S	I	R	S	S	R	S	-	S	S
3237	ERR7221517	11	03/11/2016	IRE429	127	*E. coli*	Faeces	1	blaOXA_48	R	R	R	R	R	R	S	S	R	S	S	S	S
3189	ERR7221496	1	20/04/2017	IRE617	167	*E. coli*	Faeces	1	blaNDM_5	R	R	-	-	R	R	R	R	R	R	R	R	-
3349	ERR1981374	3	15/08/2016	IRE307	38	*E. coli*	Rectal	26	blaOXA_48	R	R	R	R	R	R	S	S	R	S	S	S	S
3313	ERR7221594	3	04/01/2013	IRE681	38	*E. coli*	Urine	7	blaOXA_48	R	R	R	R	R	R	R	S	R	S	-	S	S
3193	ERR7221488	2	08/09/2014	IRE51	131	*E. coli*	Rectal	27	blaKPC_2	R	R	R	R	R	R	R	I	R	R	-	S	S
3269	ERR1837601	2	19/07/2016	IRE291	131	*E. coli*	Urine	1	blaOXA_48	R	R	S	I	I	R	R	S	R	S	S	S	S
3202	ERR1837597	6	30/05/2016	IRE268	162	*E. coli*	Rectal	27	blaKPC_2	R	R	R	R	R	R	R	I	R	S	S	S	S
3195	ERR7221503	27	17/12/2014	IRE78	409	*E. coli*	Rectal	27	blaKPC_2	R	R	R	R	R	R	S	I	R	S	-	S	S
3367	ERR7221535	2	02/03/2017	IRE564	131	*E. coli*	Rectal	28	blaOXA_48	R	R	R	R	R	R	S	S	R	S	S	S	S
3219	ERR7221473	2	30/12/2015	IRE182	131	*E. coli*	Urine	23	blaNDM_1	R	R	R	R	R	R	R	S	R	R	-	R	S
3261	ERR7221599	4	17/11/2015	IRE180	88	*E. coli*	Urine	1	blaOXA_48	R	R	R	R	I	R	R	R	R	S	-	S	S
3279	ERR1987773	2	27/09/2016	IRE362	131	*E. coli*	Faeces	1	blaOXA_48	R	R	R	R	R	S	S	S	R	S	S	S	S
3259	ERR7221591	3	07/02/2017	IRE538	38	*E. coli*	Rectal	1	blaOXA_48	R	R	R	R	I	R	S	S	R	R	S	R	S
3260	ERR7221539	11	09/03/2017	IRE574	127	*E. coli*	Rectal	1	blaOXA_48	R	R	S	S	S	S	S	S	R	S	S	S	S
3308	ERR7221475	31	07/06/2017	IRE652	648	*E. coli*	Rectal	4	blaOXA_48	S	S	-	-	S	S	S	S	S	S	S	S	-
3286	ERR2124227	40	05/09/2016	IRE325	973	*E. coli*	Faeces	1	blaOXA_48	R	R	R	R	R	R	R	S	R	S	S	S	S
3301	ERR2124251	34	25/01/2017	IRE289	297	*E. coli*	Rectal	1	blaOXA_48	R	R	R	R	I	-	I	S	R	S	S	S	S
3242	ERR7221526	1	14/11/2016	IRE440	10	*E. coli*	Faeces	1	blaOXA_48	R	R	S	S	S	S	S	S	R	S	S	R	S
3343	ERR7221500	4	27/10/2014	IRE68	410	*E. coli*	Rectal	26	blaOXA_181	R	R	R	R	R	R	R	S	R	R	-	R	-
3318	ERR7221513	55	09/12/2015	IRE191	2599	*E. coli*	Rectal	11	blaOXA_48	R	R	S	S	S	S	S	S	R	S	-	S	S
3316	ERR7221603	1	14/11/2016	IRE447	167	*E. coli*	Rectal	9	blaOXA_244	R	R	R	R	R	R	R	S	R	S	S	R	S
3329	ERR7221464	3	09/06/2015	IRE113	38	*E. coli*	Faeces	18	blaOXA_244	R	R	R	R	R	R	R	S	R	S	-	S	S
3305	ERR7221600	8	25/02/2016	IRE216	69	*E. coli*	Rectal	3	blaOXA_48	R	R	R	R	R	S	R	S	R	S	S	S	S
3370	ERR7221579	48	27/02/2017	IRE563	68	*E. coli*	Rectal	28	blaOXA_48	R	R	R	R	I	R	S	S	R	S	S	S	S
3212	ERR7221546	15	27/04/2017	IRE618	405	*E. coli*	Urine	34	blaNDM_5	-	-	-	-	-	R	-	R	-	-	-	S	R
3255	ERR7221575	1	07/12/2016	IRE392	10	*E. coli*	Rectal	1	blaOXA_48	R	R	S	I	R	R	S	S	R	S	S	S	S
3372	ERR7221604	4	06/12/2016	IRE474	410	*E. coli*	Rectal	28	blaOXA_48	R	R	R	R	R	R	S	S	R	S	S	S	S
3319	ERR7221519	3	02/11/2016	IRE423	38	*E. coli*	Urine	12	blaOXA_48	R	R	R	R	I	R	S	S	R	S	S	S	S
3333	ERR7221510	12	02/09/2015	IRE153	940	*E. coli*	Rectal	22	blaOXA_181	R	R	R	R	R	R	R	S	R	S	-	S	S
3295	ERR2124237	7	18/10/2016	IRE408	12	*E. coli*	Faeces	1	blaOXA_48	R	R	R	R	R	-	S	I	R	S	S	S	S
3230	ERR7221487	1	17/01/2017	IRE499	10	*E. coli*	Rectal	1	blaOXA_48	R	R	S	S	S	R	S	S	R	S	S	S	S
3291	ERR2124233	2	18/10/2016	IRE327	131	*E. coli*	Faeces	1	blaOXA_48	R	R	S	I	S	-	R	S	R	S	S	S	S
3197	ERR7221511	1	29/09/2015	IRE166	744	*E. coli*	Rectal	27	blaKPC_2	R	R	R	R	R	R	R	R	R	S	-	R	R
3244	ERR7221529	16	21/12/2016	IRE489	404	*E. coli*	Rectal	1	blaOXA_48	R	R	S	I	I	R	S	S	R	S	S	S	S
3331	ERR7221507	24	20/05/2015	IRE101	73	*E. coli*	Urine	22	blaOXA_48	R	R	S	S	S	R	S	S	R	S	-	S	S
3307	ERR1981602	4	26/02/2016	IRE218	410	*E. coli*	Urine	3	blaOXA_48	R	R	R	R	R	R	R	S	R	S	S	S	S
3338	ERR7221553	1	13/10/2014	IRE65	167	*E. coli*	Abdomi	23	blaOXA_244	R	R	R	R	R	R	R	S	R	R	-	R	S
3257	ERR7221538	1	08/03/2017	IRE572	10	*E. coli*	Rectal	1	blaOXA_48	R	R	S	I	I	R	S	S	R	S	S	S	S
3357	ERR1981606	2	12/07/2016	IRE288	131	*E. coli*	Rectal	27	blaOXA_48	R	R	R	R	R	R	S	S	R	R	S	R	S
3251	ERR7221570	7	04/04/2017	IRE599	12	*E. coli*	Rectal	1	blaOXA_48	R	R	-	-	S	R	S	S	R	S	S	S	S
3198	ERR7221466	2	17/08/2015	IRE137	131	*E. coli*	Wound	27	blaKPC_2	R	R	R	R	R	R	R	R	R	R	-	S	R
3290	ERR2124232	5	11/10/2016	IRE388	58	*E. coli*	Faeces	1	blaOXA_48	R	R	S	S	S	-	S	S	R	S	S	S	S
3192	ERR7221468	32	03/09/2015	IRE154	8819	*E. coli*	Rectal	27	blaKPC_2	R	R	R	R	R	R	R	R	R	S	-	-	-
3217	ERR7221597	1	10/08/2012	EM677	4981	*E. coli*	Faeces	18	blaNDM_1	R	R	R	R	R	R	R	S	R	R	-	-	S
3236	ERR7221516	54	07/11/2016	IRE432	57	*E. coli*	Faeces	1	blaOXA_48	R	R	S	S	I	R	S	S	R	S	S	R	S
3368	ERR7221560	2	19/03/2015	IRE87	131	*E. coli*	Rectal	28	blaOXA_48	R	R	S	S	S	R	S	S	R	R	-	R	S
3283	ERR1990255	42	04/10/2016	IRE372	135	*E. coli*	Faeces	1	blaOXA_48	R	R	S	R	R	R	S	I	R	S	S	S	S
3341	ERR7221477	3	12/11/2016	IRE366	38	*E. coli*	Rectal	26	blaOXA_48	R	R	R	R	R	R	R	R	R	S	S	S	S
3300	ERR2124247	2	18/01/2017	IRE511	131	*E. coli*	Rectal	1	blaOXA_48	R	R	R	R	I	-	R	S	R	R	S	R	S
3263	ERR7221540	1	13/03/2017	IRE575	10	*E. coli*	Rectal	1	blaOXA_48	R	R	S	I	I	R	S	S	R	S	S	S	S
3206	ERR7221563	21	13/11/2015	IRE178	156	*E. coli*	urine	16	blaNDM_5	R	R	R	R	R	R	R	R	R	S	S	S	S

3273	ERR1987534	10	19/09/2016	IRE343	538	*E. coli*	Faeces	1	blaOXA_48	R	R	S	I	I	R	S	I	R	S	S	S	S
3378	ERR7221533	52	10/09/2014	IRE55	120	*E. coli*	Urine	12	blaVIM_4	R	R	R	R	R	R	R	S	R	R	-	R	S
3314	ERR2124248	19	23/01/2017	IRE515	141	*E. coli*	Urine	7	blaOXA_48	R	R	S	S	S	-	S	S	R	S	S	S	S
3342	ERR7221497	5	13/10/2014	IRE63	155	*E. coli*	Anal	26	blaOXA_48	R	R	S	S	S	R	S	S	R	S	-	S	S
3366	ERR7221551	2	25/05/2017	IRE641	131	*E. coli*	SNS	28	blaOXA_48	R	R	-	-	R	R	R	S	R	S	S	S	-
3208	ERR7221569	1	13/06/2016	IRE276	167	*E. coli*	Blood	17	blaNDM_5	R	R	R	R	R	R	R	I	R	R	R	R	S
3374	ERR1981603	2	09/03/2016	IRE223	131	*E. coli*	Gallblad	28	blaOXA_48	R	R	S	I	S	R	S	I	R	S	S	S	S
3222	ERR7221476	17	08/12/2016	IRE345	349	*E. coli*	Rectal	1	blaOXA_48	R	R	R	R	R	R	R	R	R	S	S	S	S
3350	ERR2124243	2	11/01/2017	IRE480	131	*E. coli*	Drain	26	blaOXA_48	R	R	S	R	I	-	S	I	R	S	S	S	S
3324	ERR1987538	5	21/09/2016	IRE339	155	*E. coli*	Rectal	12	blaOXA_48	R	R	S	S	S	S	S	S	R	S	S	S	S
3296	ERR2124238	37	21/10/2016	IRE411	46	*E. coli*	Faeces	1	blaOXA_48	R	R	S	S	S	-	S	S	R	S	S	S	S
3210	ERR1981377	1	01/09/2016	IRE319	1702	*E. coli*	Faeces	18	blaNDM_5	R	R	R	R	R	R	R	R	R	R	R	R	S
3238	ERR7221518	14	03/11/2016	IRE428	2622	*E. coli*	Faeces	1	blaOXA_48	R	R	R	I	R	R	S	S	R	S	S	S	S
3369	ERR7221578	19	24/11/2016	IRE316	141	*E. coli*	Urine	28	blaOXA_48	R	R	R	R	I	R	S	S	R	R	I	R	S
3312	ERR7221598	3	29/07/2015	IRE132	38	*E. coli*	Rectal	30	blaOXA_48	R	R	R	R	R	S	S	S	R	R	-	R	S
3347	ERR1837604	3	18/08/2016	IRE311	38	*E. coli*	Rectal	26	blaOXA_48	R	R	R	R	R	S	S	S	R	R	S	R	S
3231	ERR7221491	15	22/02/2017	IRE557	405	*E. coli*	Rectal	1	blaOXA_48	R	R	R	R	R	R	S	S	R	S	S	S	S
3253	ERR7221537	6	08/03/2017	IRE248	162	*E. coli*	Rectal	1	blaOXA_48	R	R	S	I	I	R	S	S	R	S	I	S	S
3299	ERR2124244	1	11/01/2017	IRE499	10	*E. coli*	Rectal	1	blaOXA_48	R	R	S	S	S	-	S	S	R	S	S	S	S
3336	ERR7221495	4	19/04/2017	IRE614	23	*E. coli*	Bile	23	blaOXA_48	R	R	-	-	S	R	R	I	R	S	S	R	-
3362	ERR7221514	23	07/01/2016	IRE202	1485	*E. coli*	Rectal	28	blaOXA_48	R	R	R	R	R	R	R	R	R	S	S	S	R
3325	ERR7221601	47	27/10/2016	IRE419	361	*E. coli*	NA	15	blaOXA_244	R	R	R	R	R	R	R	S	R	S	S	S	S
3323	ERR1987532	44	14/09/2016	IRE335	1722	*E. coli*	Urine	12	blaOXA_244	R	R	R	R	R	R	R	S	R	S	S	S	S
3339	ERR1981375	3	16/08/2016	IRE310	38	*E. coli*	Urine	23	blaOXA_48	R	R	R	R	R	S	S	S	R	S	S	R	S
3365	ERR7221543	2	06/04/2017	IRE601	131	*E. coli*	Blood	28	blaOXA_48	R	R	-	-	R	R	R	I	R	R	S	R	-
3274	ERR1987535	43	16/09/2016	IRE341	7400	*E. coli*	Faeces	1	blaOXA_48	R	R	S	S	S	R	S	S	R	S	S	S	S
3346	ERR7221593	4	28/10/2014	IRE68	410	*E. coli*	Rectal	26	blaOXA_181	R	R	R	R	R	R	R	S	R	R	-	R	S
3375	ERR2124245	10	17/01/2017	IRE507	357	*E. coli*	Rectal	28	blaOXA_48	R	R	S	S	I	-	S	S	R	S	S	S	S
3280	ERR1987774	5	27/09/2016	IRE363	1049	*E. coli*	Faeces	1	blaOXA_48	R	R	S	S	I	R	S	I	R	S	S	S	S
3247	ERR7221548	4	04/05/2017	IRE409	88	*E. coli*	Blood	1	blaOXA_48	R	R	-	-	S	R	I	S	R	R	S	R	-
3281	ERR1987775	17	27/09/2016	IRE364	349	*E. coli*	Rectal	1	blaOXA_48	R	R	S	S	I	R	S	S	R	S	S	S	S
3248	ERR7221550	16	22/05/2017	IRE636	1193	*E. coli*	Rectal	1	blaOXA_48	R	R	-	-	S	R	S	S	R	S	S	S	-
3332	ERR7221509	1	29/08/2015	IRE148	10	*E. coli*	Urine	22	blaOXA_48	R	R	S	S	S	R	R	S	R	S	-	S	S
3310	ERR7221501	3	20/12/2013	IRE681	38	*E. coli*	Rectal	30	blaOXA_48	R	R	R	R	R	R	R	S	R	S	-	S	R
3213	ERR7221565	12	01/12/2015	IRE186	940	*E. coli*	Rectal	27	blaOXA_48, blaNDM_5	R	R	R	R	R	R	R	I	R	S	-	S	S
3205	ERR7221547	14	09/10/2014	IRE62	636	*E. coli*	Blood	5	blaNDM_5	R	R	R	R	R	R	R	I	R	S	-	-	-
3354	ERR7221559	25	20/01/2015	IRE82	1079	*E. coli*	Rectal	27	blaOXA_48	R	R	R	R	R	R	R	S	R	S	-	S	S
3321	ERR1981373	2	10/08/2016	IRE306	131	*E. coli*	Urine	12	blaOXA_48	R	R	S	I	I	R	S	S	R	R	S	R	S
3363	ERR7221532	6	08/02/2017	IRE542	4360	*E. coli*	Rectal	28	blaOXA_48	R	R	I	I	I	R	S	S	R	S	S	S	S
3252	ERR7221571	1	31/01/2017	IRE529	10	*E. coli*	Faeces	1	blaOXA_48	R	R	S	I	I	R	S	S	R	S	S	S	S
3285	ERR2118033	29	11/01/2017	IRE500	1236	*E. coli*	Rectal	1	blaOXA_48	R	R	R	R	R	-	S	I	R	S	S	S	S
3214	ERR7221555	4	08/03/2013	IRE686	410	*E. coli*	Sputum	1	blaNDM_1	R	R	R	R	R	R	R	R	R	R	-	R	R
3294	ERR2124236	1	18/10/2016	IRE406	7696	*E. coli*	Faeces	1	blaOXA_48	R	R	S	S	I	-	S	S	R	S	S	S	S
3227	ERR7221484	58	23/11/2016	IRE455	3018	*E. coli*	Faeces	1	blaOXA_48	R	R	R	R	R	R	S	S	R	S	S	S	S
3241	ERR7221525	7	09/11/2016	IRE439	12	*E. coli*	Rectal	1	blaOXA_48	R	R	S	I	I	R	S	I	R	S	S	S	S
3297	ERR2124239	5	21/10/2016	IRE412	58	*E. coli*	Faeces	1	blaOXA_48	R	R	S	S	S	-	S	S	R	S	S	S	S
3190	ERR2124229	5	28/09/2016	IRE370	1049	*E. coli*	Rectal	9	blaKPC_2	R	R	R	R	R	R	R	S	R	S	S	-	-
3200	ERR7221552	17	02/06/2017	IRE425	349	*E. coli*	Rectal	27	blaKPC_2	-	-	-	-	-	R	-	S	-	-	-	S	S
3334	ERR7221588	3	11/05/2015	IRE99	38	*E. coli*	Abdomi	22	blaOXA_48	R	R	R	R	R	R	R	I	R	S	-	S	S
3207	ERR7221590	1	25/01/2017	IRE520	617	*E. coli*	Urine	16	blaNDM_5	R	R	R	R	R	R	R	R	R	S	S	R	S
3235	ERR7221515	13	08/11/2016	IRE434	95	*E. coli*	Faeces	1	blaOXA_48	R	R	S	S	S	S	S	S	R	S	S	S	S
3264	ERR7221605	46	07/02/2017	IRE539	8818	*E. coli*	Environ	1	blaOXA_48	R	R	S	I	I	R	R	S	R	S	S	S	-
3268	ERR1837600	18	20/06/2016	IRE279	101	*E. coli*	Faeces	1	blaOXA_48	R	R	S	I	I	R	S	S	R	S	S	S	S
3376	ERR2124246	10	17/01/2017	IRE508	357	*E. coli*	Wound	28	blaOXA_48	R	R	R	R	R	-	S	S	R	S	S	S	S
3293	ERR2124235	5	18/10/2016	IRE404	155	*E. coli*	Faeces	1	blaOXA_48	R	R	S	I	I	-	R	S	R	S	S	S	S
3292	ERR2124234	38	18/10/2016	IRE401	1442	*E. coli*	Rectal	1	blaOXA_48	R	R	S	S	S	-	S	S	R	S	S	S	S
3337	ERR7221549	22	17/05/2017	IRE634	4959	*E. coli*	Rectal	23	blaOXA_48	R	R	-	-	S	R	S	S	R	S	S	S	-
3202	ERR1837597	6	30/05/2016	IRE268	162	*E. coli*	Rectal	27	blaKPC_2	R	R	R	R	R	R	R	I	R	S	S	S	S
3224	ERR7221481	60	07/11/2016	IRE431	8820	*E. coli*	Faeces	1	blaOXA_48	R	R	R	R	I	R	S	S	R	S	S	S	S
3345	ERR7221523	4	30/06/2014	IRE30	410	*E. coli*	Drain	26	blaOXA_181	R	R	R	R	R	R	R	S	R	R	-	R	S
3284	ERR1990256	41	05/09/2016	IRE324	7416	*E. coli*	Faeces	1	blaOXA_48	R	R	S	R	I	R	R	S	R	S	S	S	S
3306	ERR7221542	7	20/01/2014	IRE6	12	*E. coli*	Rectal	3	blaOXA_48	R	R	S	S	R	R	S	S	R	S	-	S	S
3315	ERR7221558	6	11/12/2014	IRE77	162	*E. coli*	Rectal	9	blaOXA_48	R	R	R	R	I	R	R	S	R	S	-	S	S
3245	ERR7221534	2	27/02/2017	IRE562	131	*E. coli*	Rectal	1	blaOXA_48	R	R	R	I	I	R	S	S	R	S	S	S	S
3317	ERR2124249	3	24/01/2017	IRE517	38	*E. coli*	Blood	10	blaOXA_48	R	R	R	R	R	-	S	S	R	R	S	R	S
3309	ERR7221556	3	15/11/2013	IRE715	38	*E. coli*	MSU	29	blaOXA_48	R	R	R	R	R	R	R	I	R	S	-	S	R
3234	ERR7221502	57	31/07/2013	IRE698	8822	*E. coli*	Rectal	1	blaOXA_48	R	R	S	R	I	R	S	S	R	S	-	S	S
3298	ERR2124242	26	03/01/2017	IRE492	6958	*E. coli*	Faeces	1	blaOXA_48	R	R	S	I	S	-	S	S	R	S	S	S	S
3373	ERR7221606	2	14/03/2017	IRE578	131	*E. coli*	Rectal	28	blaOXA_48	R	R	R	R	R	R	S	S	R	S	S	S	S
3379	ERR7221478	30	17/10/2016	IRE397	1727	*E. coli*	Rectal	13	blaVIM_1	R	R	R	R	R	S	R	S	R	S	S	R	S
3196	ERR7221508	13	17/08/2015	IRE141	95	*E. coli*	Rectal	27	blaKPC_2	R	R	R	R	R	R	R	I	R	S	-	S	S
3201	ERR7221576	1	15/07/2014	IRE35	10	*E. coli*	Rectal	27	blaKPC_2	R	R	R	R	R	R	R	I	R	S	-	S	R

3348	ERR1837605	20	27/08/2016	IRE308	4093	*E. coli*	Rectal	26	blaOXA_48	R	R	S	S	S	R	S	R	R	S	S	S	S
3271	ERR1837603	45	16/08/2016	IRE309	295	*E. coli*	Faeces	1	blaOXA_48	R	R	S	I	R	R	S	S	R	S	S	S	S
3226	ERR7221483	59	14/11/2016	IRE441	372	*E. coli*	Faeces	1	blaOXA_48	R	R	S	I	R	R	S	S	R	S	S	S	S
3288	ERR2124230	22	04/10/2016	IRE379	216	*E. coli*	Faeces	1	blaOXA_48	R	R	R	R	R	R	S	S	R	S	S	S	S
3302	ERR7221512	56	29/09/2015	IRE167	354	*E. coli*	Rectal	2	blaOXA_48	R	R	R	R	R	R	R	S	R	S	-	S	S
3221	ERR7221469	8	15/09/2015	IRE161	69	*E. coli*	Urine	1	blaOXA_48	R	R	S	S	S	S	S	S	R	S	-	S	S
3351	ERR2124252	33	31/01/2017	IRE528	6178	*E. coli*	Rectal	26	blaOXA_48	R	R	R	R	I	-	S	S	R	S	S	S	S
3275	ERR1987536	8	16/09/2016	IRE340	69	*E. coli*	Faeces	1	blaOXA_48	R	R	S	S	S	S	S	S	R	S	S	S	S
3278	ERR1987772	5	27/09/2016	IRE360	1049	*E. coli*	Faeces	1	blaOXA_48	R	R	S	I	I	R	S	S	R	S	S	S	S
3228	ERR7221485	28	06/12/2016	IRE456	399	*E. coli*	Rectal	1	blaOXA_48	R	R	S	S	S	S	S	S	R	S	S	S	S
3327	ERR7221530	4	17/01/2017	IRE509	367	*E. coli*	Hvs	31	blaOXA_48	R	R	S	I	I	R	S	S	R	S	S	S	S
3233	ERR7221499	2	12/06/2017	IRE657	131	*E. coli*	Rectal	1	blaOXA_48	R	R	-	-	S	R	I	S	R	R	S	R	-
3344	ERR7221522	31	23/09/2016	IRE357	648	*E. coli*	Rectal	26	blaOXA_48	R	R	R	R	I	R	R	S	R	S	S	S	S
3225	ERR7221482	16	09/11/2016	IRE438	1193	*E. coli*	Faeces	1	blaOXA_48	R	R	S	I	I	R	S	S	R	S	S	S	S
3335	ERR1981376	11	01/09/2016	IRE317	127	*E. coli*	Urine	22	blaOXA_48	R	R	S	S	S	S	S	S	R	S	S	S	S
3258	ERR7221585	5	24/05/2017	IRE638	58	*E. coli*	Rectal	1	blaOXA_48	R	R	-	-	S	R	S	S	R	S	S	S	-
3311	ERR7221561	2	08/04/2015	IRE89	131	*E. coli*	Rectal	30	blaOXA_48	R	R	R	R	R	R	S	I	R	R	-	R	S
3243	ERR7221528	1	02/12/2016	IRE472	10	*E. coli*	Rectal	1	blaOXA_48	R	R	S	I	S	R	S	S	R	S	S	S	S
3254	ERR7221574	49	06/12/2016	IRE385	28	*E. coli*	Rectal	1	blaOXA_48	R	R	R	R	S	R	R	S	R	S	S	S	S
3723	ERR7221403	-	12/06/2017	IRE658	85	*K. sp.LTGPAF-6F*	SNS	22	blaOXA_48	R	R	-	-	S	R	S	S	R	S	S	S	-
3445	ERR7221412	3	19/09/2016	IRE344	36	*K. oxytoca*	Faeces	1	blaOXA_48	R	R	S	S	I	R	S	S	R	S	S	S	S
3403	ERR7221389	1	18/02/2014	IRE14	225	*K. oxytoca*	Blood	27	blaKPC_2	R	R	R	R	R	R	R	R	R	S	-	S	S
3443	ERR7221409	2	28/07/2016	IRE299	58	*K. oxytoca*	Pleural	1	blaOXA_48	R	R	S	S	S	R	S	S	R	S	S	S	S
3425	ERR7221450	6	02/08/2016	IRE300	77	*K. oxytoca*	Faeces	1	blaOXA_48	R	R	R	R	R	R	S	S	R	S	S	S	S
3416	ERR7221433	1	10/04/2015	IRE91	225	*K. oxytoca*	Rectal	27	blaKPC_2	R	R	R	R	R	R	R	S	R	S	-	S	S
3435	ERR7221366	3	25/11/2016	IRE460	36	*K. oxytoca*	Faeces	1	blaOXA_48	R	R	R	R	R	R	S	S	R	I	I	S	S
3436	ERR7221367	2	06/12/2016	IRE475	58	*K. oxytoca*	Rectal	1	blaOXA_48	R	R	S	I	S	R	S	S	R	S	S	S	S
3390	ERR7221353	1	10/02/2014	IRE13	225	*K. oxytoca*	Rectal	27	blaKPC_2	R	R	R	R	R	R	R	R	R	S	-	S	S
3463	ERR7221452	5	08/09/2016	IRE330	199	*K. oxytoca*	Rectal	26	blaVIM_1	R	R	R	R	R	S	R	S	R	S	S	R	S
3433	ERR7221362	3	07/12/2016	IRE416	36	*K. oxytoca*	Faeces	1	blaOXA_48	R	R	S	I	R	R	R	S	R	S	S	S	S
3411	ERR7221410	1	30/06/2014	IRE31	225	*K. oxytoca*	Peritone	27	blaKPC_2	R	R	R	R	R	R	R	I	R	S	-	R	S
3395	ERR7221378	1	26/05/2015	IRE104	225	*K. oxytoca*	Sputum	27	blaKPC_2	R	R	R	R	R	R	R	I	R	S	-	S	S
3461	ERR7221418	7	08/12/2016	IRE433	223	*K. oxytoca*	Blood	1	blaVIM_1	R	R	R	R	R	S	R	S	R	R	S	R	S
3446	ERR7221414	2	28/09/2016	IRE367	58	*K. oxytoca*	Rectal	1	blaOXA_48	R	R	S	S	S	R	R	S	R	S	S	S	R
3391	ERR7221354	1	18/08/2015	IRE146	225	*K. oxytoca*	Rectal	27	blaKPC_2	R	R	R	R	R	R	R	S	R	S	-	S	R
3405	ERR7221391	1	18/08/2015	IRE143	225	*K. oxytoca*	Rectal	27	blaKPC_2	R	R	R	R	R	R	R	R	R	S	-	S	S
3399	ERR7221383	1	09/06/2015	IRE112	225	*K. oxytoca*	Rectal	27	blaKPC_2	R	R	R	R	R	R	R	I	R	S	-	S	S
3397	ERR7221381	1	02/06/2015	IRE108	225	*K. oxytoca*	Rectal	27	blaKPC_2	R	R	R	R	R	R	R	S	R	S	-	S	S
3429	ERR7221358	3	20/09/2016	IRE347	36	*K. oxytoca*	Faeces	1	blaOXA_48	R	R	S	S	S	R	S	S	R	S	S	S	S
3400	ERR7221386	1	26/06/2015	IRE121	225	*K. oxytoca*	Rectal	27	blaKPC_2	R	R	R	R	R	R	R	I	R	S	-	S	R
3387	ERR7221447	1	02/02/2014	IRE7	225	*K. oxytoca*	Rectal	27	blaKPC_2	R	R	R	R	R	R	R	S	R	S	-	S	R
3458	ERR7221368	19	31/01/2017	IRE534	229	*K. oxytoca*	Rectal	12	blaOXA_48	R	R	S	I	S	R	S	S	S	S	S	S	S
3402	ERR7221388	4	10/08/2015	IRE135	225	*K. oxytoca*	Rectal	27	blaKPC_2	R	R	R	R	R	R	R	I	R	S	-	S	R
3420	ERR7221441	28	11/01/2017	IRE501	141	*K. oxytoca*	Rectal	1	blaOXA_48	R	R	S	S	I	R	S	S	R	S	S	S	S
3447	ERR7221421	2	02/12/2016	IRE471	58	*K. oxytoca*	Rectal	1	blaOXA_48	R	R	S	S	S	R	S	S	R	S	S	S	S
3413	ERR7221425	1	19/12/2014	IRE56	225	*K. oxytoca*	Rectal	27	blaKPC_2	R	R	S	S	R	R	R	I	R	S	-	S	S
3448	ERR7221422	10	16/12/2016	IRE485	34	*K. oxytoca*	Environ	1	blaOXA_48	R	R	R	R	R	R	S	S	R	R	S	R	-
3398	ERR7221382	4	04/06/2015	IRE109	225	*K. oxytoca*	Rectal	27	blaKPC_2	R	R	R	R	R	R	R	I	R	S	-	S	S
3389	ERR7221351	1	15/07/2015	IRE128	225	*K. oxytoca*	Drain	27	blaKPC_2	R	R	R	R	R	R	R	I	R	S	-	R	R
3437	ERR7221369	18	21/03/2017	IRE584	231	*K. oxytoca*	Rectal	1	blaOXA_48	R	R	S	I	I	R	S	S	R	S	I	S	S
3453	ERR7221430	2	25/11/2016	IRE461	58	*K. oxytoca*	Faeces	1	blaOXA_48	R	R	S	S	I	R	S	S	R	S	S	S	S
3388	ERR7221448	4	13/11/2015	IRE177	225	*K. oxytoca*	Rectal	27	blaKPC_2	R	R	R	R	R	R	R	R	R	S	-	R	R
3404	ERR7221390	1	17/08/2015	IRE142	225	*K. oxytoca*	Rectal	27	blaKPC_2	R	R	R	R	R	R	R	R	R	S	-	S	S
3452	ERR7221428	2	09/05/2017	IRE628	58	*K. oxytoca*	Rectal	1	blaOXA_48	R	R	-	-	S	R	S	S	R	S	S	S	-
3412	ERR7221413	13	22/07/2014	IRE36	224	*K. oxytoca*	Rectal	27	blaKPC_2	R	R	R	R	R	R	R	I	R	S	-	S	R
3440	ERR7221385	16	26/06/2015	IRE120	228	*K. oxytoca*	Peritone	1	blaOXA_48	R	R	R	R	R	R	R	S	R	S	-	S	S
3419	ERR7221439	29	27/09/2016	IRE361	135	*K. oxytoca*	Faeces	1	blaOXA_48	R	R	R	R	R	R	S	S	R	S	S	S	S
3434	ERR7221364	20	02/11/2016	IRE422	37	*K. oxytoca*	Faeces	1	blaOXA_48	R	R	S	S	I	R	S	S	R	S	S	S	S
3422	ERR7221443	2	16/03/2017	IRE580	58	*K. oxytoca*	Rectal	1	blaOXA_48	R	R	S	S	S	R	S	S	R	S	S	S	S
3459	ERR7221446	26	12/06/2017	IRE659	199	*K. oxytoca*	Rectal	22	blaOXA_48	R	R	-	-	R	R	I	S	R	S	S	S	-
3462	ERR7221396	5	25/01/2016	IRE206	199	*K. oxytoca*	Sacrum	13	blaVIM_1	R	R	R	R	R	S	R	S	R	R	S	R	R
3418	ERR7221436	2	31/01/2017	IRE530	58	*K. oxytoca*	Urine	1	blaOXA_48	S	S	S	S	S	R	S	I	R	S	S	S	S
3386	ERR7221373	1	15/05/2017	IRE128	225	*K. oxytoca*	Rectal	12	blaKPC_2	-	-	-	-	-	R	-	R	-	-	-	R	R
3457	ERR7221355	23	22/12/2015	IRE187	230	*K. oxytoca*	Drain	12	blaOXA_48	R	R	S	S	S	R	S	S	R	S	-	S	S
3424	ERR7221449	2	07/07/2016	IRE283	58	*K. oxytoca*	Urine	1	blaOXA_48	R	R	S	S	S	S	S	S	R	S	S	S	S
3394	ERR7221377	1	25/05/2015	IRE103	225	*K. oxytoca*	Rectal	27	blaKPC_2	R	R	R	R	R	R	R	I	R	S	-	S	S
3414	ERR7221431	1	01/08/2015	IRE80	225	*K. oxytoca*	rectal	27	blaKPC_2	R	R	R	R	R	R	R	I	R	S	-	S	R
3407	ERR7221393	1	11/09/2015	IRE156	225	*K. oxytoca*	NA	27	blaKPC_2	R	R	R	R	R	R	R	R	R	S	-	-	-
3423	ERR7221445	2	12/06/2017	IRE657	58	*K. oxytoca*	Rectal	1	blaOXA_48	R	R	-	-	S	R	S	S	R	S	S	S	-
3426	ERR7221451	2	01/09/2016	IRE321	58	*K. oxytoca*	Faeces	1	blaOXA_48	R	R	S	S	S	R	S	S	R	S	S	S	S
3449	ERR7221423	2	18/01/2017	IRE512	58	*K. oxytoca*	Rectal	1	blaOXA_48	R	R	S	I	I	R	S	S	R	S	S	S	S
3428	ERR7221357	22	15/09/2016	IRE338	21	*K. oxytoca*	Urine	1	blaOXA_48	R	R	S	S	S	R	S	S	R	S	S	S	S
3451	ERR7221426	2	08/03/2017	IRE571	58	*K. oxytoca*	Rectal	1	blaOXA_48	R	R	S	I	I	R	S	S	R	S	S	S	S

3396	ERR7221380	1	27/05/2015	IRE106	225	*K. oxytoca*	Rectal	27	blaKPC_2	R	R	R	R	R	R	R	I	R	S	-	S	S
3408	ERR7221397	4	14/06/2016	IRE278	225	*K. oxytoca*	Rectal	27	blaKPC_2	R	R	R	R	R	R	R	R	R	S	S	S	R
3417	ERR7221434	1	13/04/2015	IRE92	225	*K. oxytoca*	Rectal	27	blaKPC_2	R	R	R	R	R	R	R	R	R	S	-	R	R
3427	ERR7221453	10	07/02/2017	IRE537	34	*K. oxytoca*	Rectal	1	blaOXA_48	R	R	R	R	R	R	S	S	R	R	S	R	S
3454	ERR7221454	25	07/06/2017	IRE651	88	*K. oxytoca*	Rectal	3	blaOXA_48	R	R	-	-	S	S	S	S	R	S	S	S	-
3392	ERR7221375	1	03/02/2014	IRE10	225	*K. oxytoca*	Rectal	27	blaKPC_2	R	R	R	R	R	R	R	I	R	S	-	S	S
3455	ERR7221419	11	14/11/2016	IRE442	184	*K. oxytoca*	Urine	3	blaOXA_48	R	R	R	R	R	R	R	S	R	S	S	S	S
3401	ERR7221387	1	05/08/2015	IRE133	225	*K. oxytoca*	Rectal	27	blaKPC_2	R	R	R	R	R	R	R	S	R	S	-	S	S
3439	ERR7221374	2	22/05/2017	IRE636	58	*K. oxytoca*	Rectal	1	blaOXA_48	R	R	-	-	S	R	S	S	R	S	S	S	-
3456	ERR7221427	2	07/04/2017	IRE607	58	*K. oxytoca*	Rectal	4	blaOXA_48	R	R	-	-	S	R	S	S	R	-	S	S	-
3382	ERR7221416	9	17/01/2014	IRE4	178	*K. oxytoca*	Faeces	18	blaIMP_4	R	R	R	R	R	R	R	S	R	S	-	-	-
3384	ERR7221399	8	06/01/2016	IRE201	222	*K. oxytoca*	Rectal	26	blaIMP_70	R	R	R	R	R	R	R	S	R	S	S	-	-
3438	ERR7221370	2	30/03/2017	IRE595	58	*K. oxytoca*	Rectal	1	blaOXA_48	R	R	S	I	I	R	S	S	R	S	S	S	S
3393	ERR7221376	1	25/05/2015	IRE102	225	*K. oxytoca*	Rectal	27	blaKPC_2	R	R	R	R	R	R	R	I	R	S	-	S	S
3460	ERR7221444	27	14/04/2017	IRE598	183	*K. oxytoca*	Rectal	26	blaOXA_48	R	R	-	-	S	R	S	I	R	S	S	S	-
3444	ERR7221411	14	19/09/2016	IRE342	201	*K. oxytoca*	Urine	1	blaOXA_48	R	R	S	S	R	R	S	S	R	S	S	S	S
3466	ERR7221429	7	16/05/2017	IRE632	223	*K. oxytoca*	Faecal	26	blaVIM_1	R	R	-	-	R	S	R	S	R	S	S	R	-
3441	ERR7221405	15	22/11/2016	IRE455	49	*K. oxytoca*	Faeces	1	blaOXA_48	R	R	S	I	I	R	R	S	R	S	S	S	S
3465	ERR7221394	5	18/09/2015	IRE163	199	*K. oxytoca*	Rectal	26	blaVIM_1	R	R	R	R	R	S	R	S	R	S	-	R	S
3450	ERR7221424	2	14/02/2017	IRE550	58	*K. oxytoca*	Rectal	1	blaOXA_48	R	R	S	I	S	S	S	I	R	S	S	S	S
3421	ERR7221442	2	09/03/2017	IRE573	58	*K. oxytoca*	Environ	1	blaOXA_48	R	R	S	I	I	R	S	S	R	S	S	S	-
3464	ERR7221352	24	16/05/2017	IRE631	172	*K. oxytoca*	Rectal	26	blaVIM_1	R	R	-	-	R	S	R	S	R	S	S	R	-
3381	ERR7221371	9	14/04/2017	IRE613	178	*K. oxytoca*	Vulval	18	blaIMP_4	-	-	-	-	-	R	-	S	-	-	-	R	S
3415	ERR7221432	1	09/03/2015	IRE85	225	*K. oxytoca*	Rectal	27	blaKPC_2	R	R	R	R	R	R	R	I	R	S	-	S	S
3380	ERR7221372	17	19/04/2017	IRE610	50	*K. oxytoca*	Urine	2	blaIMP_4	-	-	-	-	-	R	-	S	-	-	-	R	R
3383	ERR7221384	8	19/06/2015	IRE117	222	*K. oxytoca*	Rectal	26	blaIMP_70	R	R	R	R	R	R	R	S	R	S	-	R	R
3385	ERR7221415	12	04/10/2016	IRE374	180	*K. oxytoca*	NA	1	blaKPC_2	R	R	R	R	R	R	S	I	R	S	S	R	R
3430	ERR7221359	3	22/09/2016	IRE354	36	*K. oxytoca*	Faeces	1	blaOXA_48	R	R	S	S	S	R	S	S	R	S	S	S	S
3409	ERR7221400	1	23/04/2014	IRE20	225	*K. oxytoca*	Rectal	27	blaKPC_2	R	R	R	R	R	R	R	I	R	S	-	S	S
3431	ERR7221360	6	26/09/2016	IRE358	77	*K. oxytoca*	Faeces	1	blaOXA_48	R	R	R	S	R	R	S	S	R	S	S	S	S
3432	ERR7221361	21	04/10/2016	IRE372	40	*K. oxytoca*	Faeces	1	blaOXA_48	R	R	S	S	S	S	S	S	R	S	S	S	S
3442	ERR7221406	6	16/09/2016	IRE339	77	*K. oxytoca*	Faeces	1	blaOXA_48	R	R	R	R	R	R	S	S	R	S	S	S	S
3410	ERR7221401	1	29/06/2015	IRE122	225	*K. oxytoca*	Rectal	27	blaKPC_2	R	R	R	R	R	R	R	I	R	S	-	S	S
3406	ERR7221392	4	31/08/2015	IRE151	225	*K. oxytoca*	Rectal	27	blaKPC_2	R	R	R	R	R	R	R	R	R	S	-	S	S
3640	ERR2118039	56	27/03/2017	IRE589	528	*K. pneumoniae*	Rectal	22	blaOXA_48	R	R	R	R	R	-	R	R	R	S	S	S	S
3583	ERR7221316	3	29/10/2013	IRE712	133	*K. pneumoniae*	MSU	1	blaOXA_48	R	R	R	R	R	R	S	R	R	R	-	R	R
3534	ERR2106615	1	27/02/2017	IRE24	512	*K. pneumoniae*	Sputum	27	blaKPC_3	R	R	R	R	R	-	R	R	R	S	R	R	S
3506	ERR7221345	1	03/12/2014	IRE74	258	*K. pneumoniae*	Rectal	27	blaKPC_2	R	R	R	R	R	R	R	R	R	S	-	R	R
3648	ERR7221177	22	02/06/2017	IRE650	2974	*K. pneumoniae*	Rectal	26	blaOXA_48	R	R	-	-	-	R	S	I	-	S	S	S	-
3516	ERR7221232	10	11/07/2014	IRE33	922	*K. pneumoniae*	NA	27	blaKPC_2	R	R	R	R	R	R	R	R	R	S	-	R	S
3622	ERR7221168	3	19/08/2013	IRE34	133	*K. pneumoniae*	Urine	5	blaOXA_48	R	R	R	R	R	R	S	I	R	R	-	R	R
3620	ERR1813604	1	11/03/2016	IRE224	11	*K. pneumoniae*	Rectal	3	blaOXA_48	R	R	R	R	R	R	R	R	R	R	S	R	R
3562	ERR7221342	6	21/12/2013	IRE718	323	*K. pneumoniae*	MSU	22	blaNDM_1	R	R	R	R	R	R	R	R	R	S	-	R	R
3667	ERR7221332	12	06/04/2017	IRE602	23	*K. pneumoniae*	Urine	28	blaOXA_48	R	R	-	-	S	R	R	R	R	R	R	R	-
3526	ERR1811837	8	01/03/2016	IRE219	26	*K. pneumoniae*	Rectal	27	blaKPC_2	R	R	R	R	R	R	R	R	R	S	S	R	R
3632	ERR1840990	18	30/08/2016	IRE315	45	*K. pneumoniae*	NA	12	blaOXA_48	R	R	R	R	R	R	S	I	R	S	S	R	R
3596	ERR7221269	7	02/12/2016	IRE470	37	*K. pneumoniae*	Rectal	1	blaOXA_48	R	R	S	I	I	R	R	I	R	S	S	S	S
3655	ERR2109157	-	27/01/2017	IRE524	3520	*K. pneumoniae*	Rectal	26	blaOXA_181	R	R	S	S	S	-	S	S	R	S	S	S	S
3704	ERR7221274	2	19/08/2014	IRE49	111	*K. pneumoniae*	Rectal	27	blaNDM_1	R	R	R	R	R	R	R	R	R	R	-	R	S
3473	ERR7221437	-	28/11/2016	IRE463	250	*K. pneumoniae*	Rectal	13	blaKPC_2	R	R	R	R	R	R	R	R	R	S	S	R	S
3603	ERR1830485	9	01/04/2016	IRE249	834	*K. pneumoniae*	Urine	1	blaOXA_48	R	R	S	S	S	R	S	I	R	S	S	S	R
3686	ERR1813606	4	11/03/2016	IRE226	20	*K. pneumoniae*	Rectal	28	blaOXA_48	R	R	R	R	R	R	S	S	R	S	S	S	R
3611	ERR2118032	4	11/01/2017	IRE499	336	*K. pneumoniae*	Rectal	1	blaOXA_48	R	R	R	R	R	-	S	R	R	S	I	R	S
3652	ERR7221202	1	15/06/2015	IRE115	11	*K. pneumoniae*	Rectal	26	blaOXA_48	R	R	R	R	R	R	R	I	R	R	-	R	S
3651	ERR7221218	1	07/10/2015	IRE168	11	*K. pneumoniae*	Rectal	26	blaOXA_48	R	R	R	R	R	R	R	S	R	R	-	R	R
3658	ERR7221289	48	06/04/2017	IRE604	191	*K. pneumoniae*	NA	27	blaOXA_48	R	R	-	-	-	R	S	R	-	S	S	S	-
3558	ERR1981103	26	21/09/2016	IRE353	3181	*K. pneumoniae*	BAL	12	blaNDM_1	R	R	R	R	R	R	R	R	R	S	S	R	R
3606	ERR1840996	4	27/09/2016	IRE324	336	*K. pneumoniae*	Faeces	1	blaOXA_48	R	R	R	R	R	R	S	R	R	S	S	R	S
3510	ERR7221216	8	23/09/2015	IRE164	26	*K. pneumoniae*	Rectal	27	blaKPC_2	R	R	R	R	R	R	S	R	R	S	-	R	R
3661	ERR7221186	23	25/01/2017	IRE507	240	*K. pneumoniae*	Rectal	28	blaOXA_48	R	R	S	S	I	R	S	S	R	S	S	S	S
3595	ERR7221265	4	14/11/2016	IRE444	336	*K. pneumoniae*	Faeces	1	blaOXA_48	R	R	R	R	R	R	R	S	R	S	I	R	S
3671	ERR7221214	3	16/09/2015	IRE649	133	*K. pneumoniae*	Illeosto	28	blaOXA_48	R	R	S	S	S	R	S	S	R	R	-	S	R
3525	ERR7221294	1	21/10/2014	IRE67	512	*K. pneumoniae*	Rectal	27	blaKPC_3	R	R	R	R	R	R	R	R	R	I	-	S	R
3647	ERR7221158	5	16/12/2015	IRE172	15	*K. pneumoniae*	Rectal	25	blaOXA_48	R	R	R	R	R	R	S	S	R	R	-	R	R
3623	ERR7221169	3	14/07/2014	IRE34	133	*K. pneumoniae*	Urine	5	blaOXA_48	R	R	R	R	R	R	S	R	R	R	-	R	S
3567	ERR7221153	5	01/09/2015	IRE134	15	*K. pneumoniae*	Rectal	27	blaNDM_1	R	R	R	R	R	R	R	R	R	R	-	R	R
3630	ERR7221315	15	21/10/2013	IRE711	392	*K. pneumoniae*	Rectal	12	blaOXA_48	R	R	R	R	R	R	R	S	R	R	-	R	R
3673	ERR7221254	5	09/11/2015	IRE176	3514	*K. pneumoniae*	urine	28	blaOXA_48	R	R	R	R	S	R	R	S	R	S	-	S	-
3664	ERR7221321	5	20/04/2015	IRE94	3514	*K. pneumoniae*	BLC	28	blaOXA_48	R	R	S	S	S	R	S	S	R	S	-	S	R
3530	ERR1829912	20	21/03/2016	IRE237	27	*K. pneumoniae*	Rectal	27	blaKPC_2	R	R	R	R	R	R	R	R	R	S	S	R	R
3531	ERR1829913	1	14/04/2016	IRE255	512	*K. pneumoniae*	Urine	27	blaKPC_3	R	R	R	R	R	R	R	R	R	S	R	S	S
3571	ERR7221176	30	04/05/2017	IRE409	551	*K. pneumoniae*	Blood	1	blaOXA_48	R	R	-	-	R	R	S	S	R	S	S	S	-
3682	ERR7221293	19	08/06/2017	IRE655	1322	*K. pneumoniae*	Urine	28	blaOXA_48	R	R	-	-	S	R	S	I	R	S	S	S	-

3523	ERR7221267	1	11/08/2014	IRE46	258	*K. pneumoniae*	Urine	27	blaKPC_2	R	R	R	R	R	R	R	R	R	S	-	S	S
3653	ERR1829922	50	08/08/2016	IRE304	160	*K. pneumoniae*	Rectal	26	blaOXA_48	R	R	S	I	I	R	S	R	R	S	S	S	R
3646	ERR1811835	-	08/02/2016	IRE213	2981	*K. pneumoniae*	Pus	23	blaOXA_48	R	R	S	S	S	R	S	S	R	S	S	S	R
3543	ERR7221287	26	14/03/2017	IRE363	3181	*K. pneumoniae*	Urine	6	blaNDM_1	R	R	R	R	R	R	R	R	R	S	I	R	S
3467	ERR7221276	2	28/08/2014	IRE50	111	*K. pneumoniae*	Urine	10	blaNDM_1, blaKPC_2	R	R	R	R	R	R	R	R	R	R	-	R	R
3551	ERR1829914	2	18/04/2016	IRE257	111	*K. pneumoniae*	Rectal	10	blaNDM_1	R	R	R	R	R	R	R	R	R	S	S	S	R
3593	ERR7221253	5	07/07/2015	IRE125	14	*K. pneumoniae*	blood	1	blaOXA_48	R	R	S	I	I	R	R	S	R	S	-	S	R
3668	ERR7221333	39	06/04/2017	IRE603	1307	*K. pneumoniae*	Urine	28	blaOXA_48	R	R	-	-	R	R	S	I	R	S	S	S	-
3624	ERR7221208	1	13/08/2015	IRE136	11	*K. pneumoniae*	Rectal	7	blaOXA_48	R	R	R	R	R	R	R	S	R	R	-	R	R
3552	ERR1829916	2	21/04/2016	IRE259	111	*K. pneumoniae*	Rectal	10	blaNDM_1	R	R	R	R	R	R	R	R	R	S	S	S	R
3517	ERR7221234	1	28/07/2014	IRE38	258	*K. pneumoniae*	Urine	27	blaKPC_2	R	R	R	R	R	R	R	R	R	I	-	S	S
3519	ERR7221245	14	30/03/2017	IRE593	29	*K. pneumoniae*	Rectal	27	blaKPC_2	R	R	R	R	R	R	R	R	R	S	S	R	S
3490	ERR7221335	11	29/05/2017	IRE643	307	*K. pneumoniae*	Urine	25	blaKPC_3	-	-	-	-	R	R	-	R	R	-	-	R	R
3497	ERR7221192	34	16/12/2014	IRE78	490	*K. pneumoniae*	Rectal	27	blaKPC_2	R	R	R	R	R	R	R	I	R	R	-	S	R
3582	ERR7221307	3	30/07/2013	IRE700	133	*K. pneumoniae*	Rectal	1	blaOXA_48	R	R	R	R	R	R	S	I	R	R	-	R	R
3509	ERR7221215	1	31/03/2014	IRE16	512	*K. pneumoniae*	Rectal	27	blaKPC_3	R	R	R	R	R	R	R	I	R	S	-	S	R
3621	ERR7221190	4	26/04/2017	IRE618	20	*K. pneumoniae*	Rectal	4	blaOXA_48	R	R	-	-	S	R	R	I	R	-	S	R	-
3545	ERR7221334	9	08/05/2017	IRE627	36	*K. pneumoniae*	Rectal	10	blaNDM_1	R	R	-	-	S	R	R	S	R	S	S	S	-
3579	ERR7221191	3	15/07/2013	IRE698	133	*K. pneumoniae*	Urine	1	blaOXA_48	R	R	R	R	R	R	S	S	R	R	-	R	R
3518	ERR7221201	25	09/06/2015	IRE111	1380	*K. pneumoniae*	Rectal	27	blaKPC_2	R	R	R	R	R	R	R	R	R	S	-	R	S
3573	ERR7221179	-	09/11/2016	IRE438	3516	*K. pneumoniae*	Faeces	1	blaOXA_48	R	R	S	S	S	R	R	S	R	S	S	S	S
3706	ERR7221285	24	18/09/2014	IRE57	101	*K. pneumoniae*	Urine	27	blaNDM_1	R	R	R	R	R	R	R	I	R	S	-	R	R
3654	ERR2106420	22	21/03/2017	IRE585	2974	*K. pneumoniae*	Rectal	26	blaOXA_48	R	R	R	R	R	-	R	S	R	S	S	S	R
3505	ERR7221211	25	31/08/2015	IRE149	1380	*K. pneumoniae*	Urine	27	blaKPC_2	R	R	R	R	R	R	S	R	R	S	-	R	R
3616	ERR7221318	15	01/06/2015	IRE79	147	*K. pneumoniae*	urine	3	blaOXA_48	R	R	R	R	R	R	R	R	R	S	-	R	R
3643	ERR7221196	24	09/04/2015	IRE90	101	*K. pneumoniae*	Sputum	23	blaOXA_48	R	R	R	R	R	R	R	R	R	S	-	R	R
3568	ERR7221161	29	02/12/2015	IRE187	34	*K. pneumoniae*	Drainag	1	blaOXA_48	R	R	R	R	R	R	R	R	R	S	-	S	R
3483	ERR7221340	1	05/12/2014	IRE75	258	*K. pneumoniae*	Stool	18	blaKPC_2	R	R	R	R	R	R	R	R	R	S	-	S	R
3639	ERR7221167	21	07/11/2016	IRE338	37	*K. pneumoniae*	Sacral a	22	blaOXA_48	R	R	R	R	R	R	S	S	R	R	S	R	S
3537	ERR1981104	13	14/09/2016	IRE336	48	*K. pneumoniae*	Urine	28	blaNDM_5	R	R	R	R	R	R	R	S	R	S	S	S	S
3599	ERR7221280	46	26/01/2017	IRE523	252	*K. pneumoniae*	Rectal	1	blaOXA_48	R	R	S	S	I	R	S	I	R	S	S	S	S
3578	ERR7221189	33	30/03/2017	IRE597	113	*K. pneumoniae*	Nephros	1	blaOXA_48	R	R	S	S	I	R	R	I	R	S	S	S	S
3676	ERR7221263	7	02/11/2016	IRE426	37	*K. pneumoniae*	Wound	28	blaOXA_48	R	R	R	R	R	R	R	I	R	S	S	R	R
3666	ERR7221329	38	13/02/2017	IRE549	2891	*K. pneumoniae*	Tip	28	blaOXA_48	R	R	R	S	I	R	S	S	R	S	S	S	S
3584	ERR7221336	3	19/07/2013	IRE699	133	*K. pneumoniae*	Rectal	1	blaOXA_48	R	R	R	R	R	R	S	S	R	R	-	R	R
3609	ERR1841001	4	04/10/2016	IRE378	3515	*K. pneumoniae*	Faeces	1	blaOXA_48	R	R	R	R	I	R	R	R	R	S	S	S	S
3670	ERR7221343	12	13/04/2015	IRE93	23	*K. pneumoniae*	Sputum	28	blaOXA_48	R	R	S	I	I	R	S	S	R	R	-	R	R
3511	ERR7221221	1	23/04/2014	IRE19	258	*K. pneumoniae*	Rectal	27	blaKPC_2	R	R	R	R	R	R	R	R	R	S	-	R	R
3594	ERR7221257	14	02/12/2015	IRE188	29	*K. pneumoniae*	Sputum	1	blaOXA_48	R	R	S	S	I	R	S	R	R	S	S	S	-
3485	ERR7221288	1	29/09/2014	IRE60	258	*K. pneumoniae*	Faeces	18	blaKPC_2	R	R	R	R	R	R	R	I	R	S	-	R	S
3561	ERR7221252	5	09/06/2015	IRE114	14	*K. pneumoniae*	Faeces	18	blaNDM_1	R	R	R	R	R	R	R	R	R	R	-	R	R
3541	ERR7221247	1	25/09/2014	IRE39	11	*K. pneumoniae*	NA	3	blaNDM_1	R	R	R	R	R	R	R	R	R	R	-	R	R
3645	ERR7221235	3	05/08/2014	IRE40	133	*K. pneumoniae*	Urine	23	blaOXA_48	R	R	R	R	R	R	S	I	R	R	-	R	S
3614	ERR7221310	3	09/02/2013	IRE704	133	*K. pneumoniae*	Line Tip	3	blaOXA_48	R	R	R	R	R	R	S	S	R	R	-	R	R
3689	ERR1829918	7	30/05/2016	IRE267	37	*K. pneumoniae*	Stoma	28	blaOXA_48	R	R	R	R	R	R	R	I	R	R	S	R	R
3491	ERR7221222	6	17/12/2015	IRE195	323	*K. pneumoniae*	Rectal	26	blaKPC_2	R	R	R	R	R	R	R	R	R	R	-	S	S
3528	ERR1815068	1	30/05/2016	IRE271	512	*K. pneumoniae*	Rectal	27	blaKPC_3	R	R	R	R	R	R	R	R	R	S	R	R	R
3495	ERR7221159	1	16/04/2014	IRE18	512	*K. pneumoniae*	Rectal	27	blaKPC_3	R	R	R	R	R	R	R	R	R	S	-	R	R
3617	ERR7221338	3	30/07/2013	IRE701	133	*K. pneumoniae*	NA	3	blaOXA_48	R	R	R	R	R	R	S	S	R	R	-	R	S
3607	ERR1840997	17	27/09/2016	IRE365	1373	*K. pneumoniae*	Faeces	1	blaOXA_48	R	R	R	R	R	R	R	I	R	R	S	R	S
3691	ERR1830484	4	22/03/2016	IRE240	20	*K. pneumoniae*	Rectal	28	blaOXA_48	R	R	R	R	R	R	R	S	R	S	S	S	R
3508	ERR7221213	8	14/09/2015	IRE158	26	*K. pneumoniae*	Rectal	27	blaKPC_2	R	R	R	R	R	R	R	R	R	S	-	R	R
3576	ERR7221185	4	13/01/2017	IRE499	336	*K. pneumoniae*	Faeces	1	blaOXA_48	R	R	R	R	R	R	R	I	R	S	I	R	S
3625	ERR7221273	3	15/12/2016	IRE484	133	*K. pneumoniae*	Urine	8	blaOXA_48	R	R	R	R	R	R	S	R	R	R	S	R	S
3469	ERR1840995	1	26/09/2016	IRE358	258	*K. pneumoniae*	Faeces	1	blaKPC_3	R	R	R	R	R	R	R	I	R	S	R	R	R
3487	ERR7221302	1	08/05/2013	IRE691	258	*K. pneumoniae*	Tip	21	blaKPC_2	R	R	R	R	R	R	R	R	R	S	-	R	R
3557	ERR1981101	2	21/03/2016	IRE238	111	*K. pneumoniae*	Rectal	10	blaNDM_1	R	R	R	R	R	R	R	I	R	S	S	S	R
3631	ERR1813608	27	15/03/2016	IRE233	3519	*K. pneumoniae*	Urine	12	blaOXA_48	R	R	R	R	R	R	S	S	R	S	S	S	R
3672	ERR7221242	19	18/11/2016	IRE452	1322	*K. pneumoniae*	Urine	28	blaOXA_48	R	R	S	R	S	R	S	S	R	S	S	S	S
3587	ERR7221347	3	24/12/2013	IRE720	133	*K. pneumoniae*	Urine	1	blaOXA_48	R	R	R	R	R	R	S	I	R	R	-	R	R
3590	ERR7221219	41	27/11/2015	IRE185	2891	*K. pneumoniae*	Urine	1	blaOXA_48	R	R	S	S	R	R	S	I	R	S	-	S	R
3479	ERR2118037	6	25/01/2017	IRE522	323	*K. pneumoniae*	Urine	13	blaKPC_2	R	R	R	R	R	R	R	S	R	R	I	S	R
3555	ERR1830479	2	30/03/2016	IRE247	111	*K. pneumoniae*	Rectal	10	blaNDM_1	R	R	R	R	R	R	R	I	R	S	S	S	R
3697	ERR7221220	42	20/10/2015	IRE173	274	*K. pneumoniae*	Rectal	27	blaNDM_1	R	R	R	R	R	R	R	R	R	R	-	R	R
3480	ERR7221311	36	09/04/2013	IRE705	252	*K. pneumoniae*	NA	16	blaKPC_2	R	R	R	R	R	R	R	R	R	S	-	R	R
3575	ERR7221182	4	25/11/2016	IRE460	336	*K. pneumoniae*	Faeces	1	blaOXA_48	R	R	R	I	R	R	S	I	R	I	I	R	S
3559	ERR7221157	11	09/10/2015	IRE169	307	*K. pneumoniae*	Urine	14	blaNDM_1	R	R	R	R	R	R	R	R	R	R	-	R	R
3588	ERR7221348	3	19/11/2013	IRE717	133	*K. pneumoniae*	R heel	1	blaOXA_48	R	R	R	R	R	R	S	R	R	R	-	R	R
3560	ERR7221200	1	04/02/2014	IRE11	11	*K. pneumoniae*	Urine	16	blaNDM_1	R	R	R	R	R	R	R	R	R	R	-	R	R
3544	ERR7221309	5	30/08/2013	IRE703	15	*K. pneumoniae*	Urine	9	blaNDM_1	R	R	R	R	R	R	R	R	R	S	-	R	R
3586	ERR7221344	17	07/06/2017	IRE291	1373	*K. pneumoniae*	Urine	1	blaOXA_48	R	R	-	-	S	R	S	I	R	R	S	R	-
3665	ERR7221328	37	10/02/2017	IRE546	661	*K. pneumoniae*	Sacral	28	blaOXA_48	R	R	R	R	R	R	R	R	R	S	S	S	R

3477	ERR1811831	6	19/04/2016	IRE258	323	*K. pneumoniae*	Blood	13	blaKPC_2	R	R	R	R	R	R	R	R	R	R	S	R	S
3605	ERR1840993	52	21/09/2016	IRE350	857	*K. pneumoniae*	Faeces	1	blaOXA_48	R	R	R	R	R	R	S	I	R	S	S	R	S
3612	ERR7221327	6	08/12/2016	IRE478	323	*K. pneumoniae*	Rectal	2	blaOXA_48	R	R	S	I	I	R	S	I	R	R	S	R	S
3522	ERR7221204	1	04/02/2014	IRE12	258	*K. pneumoniae*	Rectal	27	blaKPC_2	R	R	R	R	R	R	R	R	R	S	-	R	S
3496	ERR7221163	1	30/05/2014	IRE24	512	*K. pneumoniae*	Wound	27	blaKPC_3	R	R	R	R	R	R	R	I	R	S	-	S	S
3507	ERR7221346	1	24/11/2014	IRE73	258	*K. pneumoniae*	Rectal	27	blaKPC_2	R	R	R	R	R	R	R	R	R	I	-	S	S
3680	ERR7221283	19	10/02/2017	IRE547	1322	*K. pneumoniae*	Skin	28	blaOXA_48	R	R	S	S	I	R	S	S	R	S	S	S	S
3693	ERR2118030	7	10/01/2017	IRE497	309	*K. pneumoniae*	Plaque	28	blaOXA_48	R	R	S	I	I	-	S	I	R	S	S	S	S
3674	ERR7221258	12	09/12/2015	IRE190	23	*K. pneumoniae*	rectal	28	blaOXA_48	R	R	S	R	I	R	R	R	R	R	-	R	R
3548	ERR1810609	2	15/03/2016	IRE232	111	*K. pneumoniae*	Urine	10	blaNDM_1	R	R	R	R	R	R	R	I	R	S	I	S	R
3471	ERR7221299	1	14/03/2013	IRE687	258	*K. pneumoniae*	Rectal	3	blaKPC_2	R	R	R	R	R	R	R	S	R	S	-	S	S
3701	ERR7221239	2	11/08/2014	IRE43	111	*K. pneumoniae*	Rectal	27	blaNDM_1	R	R	R	R	R	R	R	R	R	R	-	R	S
3694	ERR2118036	55	17/01/2017	IRE510	151	*K. pneumoniae*	Sputum	28	blaOXA_48	R	R	R	R	R	-	R	R	R	S	S	S	R
3556	ERR1830480	2	31/03/2016	IRE248	111	*K. pneumoniae*	Rectal	10	blaNDM_1	R	R	R	R	R	R	R	I	R	S	S	S	R
3638	ERR2118031	54	11/01/2017	IRE498	3521	*K. pneumoniae*	Rectal	19	blaOXA_48	R	R	S	S	S	-	S	S	R	S	S	S	S
3538	ERR2118027	13	30/11/2016	IRE465	48	*K. pneumoniae*	Rectal	28	blaNDM_5	R	R	R	R	R	-	R	I	R	S	S	S	S
3657	ERR7221194	9	19/01/2015	IRE81	268	*K. pneumoniae*	Sputum	27	blaOXA_48	R	R	S	S	I	R	S	S	R	S	-	S	S
3695	ERR2118025	13	24/11/2016	IRE458	48	*K. pneumoniae*	Rectal	28	blaOXA_48, blaNDM_5	R	R	R	R	R	-	R	I	R	S	S	S	R
3669	ERR7221339	40	20/11/2013	IRE716	791	*K. pneumoniae*	Urine	28	blaOXA_48	R	R	S	I	R	R	R	I	R	S	-	S	R
3699	ERR7221228	2	24/06/2014	IRE27	111	*K. pneumoniae*	Rectal	27	blaNDM_1	R	R	R	R	R	R	R	R	R	R	-	R	R
3468	ERR1815070	1	23/08/2016	IRE313	258	*K. pneumoniae*	Faeces	1	blaKPC_3	R	R	R	R	R	R	R	I	R	S	R	R	R
3503	ERR7221322	1	23/04/2015	IRE97	258	*K. pneumoniae*	Rectal	27	blaKPC_2	R	R	R	R	R	R	R	R	R	S	-	R	R
3550	ERR1815067	9	30/03/2016	IRE244	36	*K. pneumoniae*	Urine	10	blaNDM_1	R	R	R	R	R	R	R	S	R	S	S	S	S
3527	ERR1813607	1	15/03/2016	IRE231	512	*K. pneumoniae*	Urine	27	blaKPC_3	R	R	R	R	R	R	R	R	R	S	R	R	R
3546	ERR7221225	2	14/05/2014	IRE23	111	*K. pneumoniae*	Urine	10	blaNDM_1	R	R	R	R	R	R	R	R	R	R	-	R	R
3642	ERR7221172	4	12/12/2016	IRE378	3515	*K. pneumoniae*	Rectal	23	blaOXA_48	R	R	S	S	S	R	S	I	R	S	S	S	S
3513	ERR7221226	1	17/06/2014	IRE25	512	*K. pneumoniae*	Rectal	27	blaKPC_3	R	R	R	R	R	R	R	R	R	S	-	R	R
3707	ERR2118026	2	29/11/2016	IRE464	111	*K. pneumoniae*	Rectal	27	blaNDM_1	R	R	R	R	R	-	R	R	R	R	R	R	S
3619	ERR7221275	3	20/01/2014	IRE5	133	*K. pneumoniae*	Rectal	3	blaOXA_48	R	R	R	R	R	R	S	S	R	R	-	R	R
3644	ERR7221324	15	12/05/2015	IRE39	147	*K. pneumoniae*	Blood	23	blaOXA_48	R	R	R	R	R	R	R	R	R	R	-	R	R
3566	ERR2106419	1	25/03/2017	IRE587	11	*K. pneumoniae*	Rectal	26	blaNDM_1	R	R	R	R	R	-	R	I	R	R	R	R	S
3678	ERR7221278	23	25/01/2017	IRE518	240	*K. pneumoniae*	Rectal	28	blaOXA_48	R	R	S	I	I	R	S	S	R	S	S	S	S
3484	ERR7221236	-	08/08/2014	IRE41	3517	*K. pneumoniae*	Faeces	18	blaKPC_2	R	R	R	R	R	R	R	R	R	S	-	R	R
3598	ERR7221277	4	23/01/2017	IRE516	336	*K. pneumoniae*	Rectal	1	blaOXA_48	R	R	R	R	R	R	S	S	R	S	S	R	S
3675	ERR7221262	7	20/09/2016	IRE348	37	*K. pneumoniae*	Urine	28	blaOXA_48	R	R	R	R	R	R	S	S	R	R	S	R	R
3628	ERR7221438	16	09/09/2016	IRE331	13	*K. pneumoniae*	Rectal	12	blaOXA_48	R	R	R	R	R	S	S	S	R	S	S	S	S
3474	ERR7221240	6	04/11/2016	IRE430	323	*K. pneumoniae*	Blood	13	blaKPC_2	R	R	R	R	R	R	R	R	R	R	S	R	R
3633	ERR7221295	16	08/01/2013	IRE682	13	*K. pneumoniae*	Urine	15	blaOXA_48	R	R	S	S	S	R	S	S	R	S	-	S	R
3659	ERR7221151	5	23/06/2015	IRE118	3514	*K. pneumoniae*	Blood	28	blaOXA_48	R	R	S	I	R	R	R	S	R	S	-	S	R
3601	ERR1811833	3	25/02/2016	IRE217	133	*K. pneumoniae*	Urine	1	blaOXA_48	R	R	S	S	I	R	S	S	R	R	S	R	R
3641	ERR2118040	5	27/03/2017	IRE592	14	*K. pneumoniae*	Blood	22	blaOXA_48	R	R	R	R	R	-	R	R	R	S	S	S	S
3488	ERR7221349	1	30/10/2013	IRE713	258	*K. pneumoniae*	Rectal	21	blaKPC_2	R	R	R	R	R	R	R	I	R	I	-	R	R
3705	ERR7221281	2	28/08/2014	IRE53	111	*K. pneumoniae*	Rectal	27	blaNDM_1	R	R	R	R	R	R	R	R	R	R	-	R	S
3524	ERR7221205	8	03/07/2015	IRE124	26	*K. pneumoniae*	Rectal	27	blaKPC_2	R	R	R	R	R	R	R	R	R	S	-	R	R
3494	ERR7221156	8	18/08/2015	IRE146	26	*K. pneumoniae*	Rectal	27	blaKPC_2	R	R	R	R	R	R	R	R	R	S	-	R	R
3489	ERR7221296	1	15/01/2013	IRE683	258	*K. pneumoniae*	Rectal	21	blaKPC_2	R	R	R	R	R	R	R	R	R	S	-	R	R
3486	ERR7221297	1	31/01/2013	IRE684	258	*K. pneumoniae*	Rectal	21	blaKPC_2	R	R	R	R	R	R	R	R	R	S	-	R	R
3574	ERR7221180	-	06/12/2016	IRE456	3516	*K. pneumoniae*	Rectal	1	blaOXA_48	R	R	S	S	S	R	R	R	R	S	S	S	S
3549	ERR1815066	2	30/03/2016	IRE243	111	*K. pneumoniae*	Rectal	10	blaNDM_1	R	R	R	R	R	R	R	I	R	S	S	S	R
3692	ERR2118029	7	10/01/2017	IRE496	309	*K. pneumoniae*	Toe	28	blaOXA_48	R	R	S	R	R	-	S	R	R	S	S	S	S
3618	ERR7221246	15	05/08/2014	IRE39	147	*K. pneumoniae*	Rectal	3	blaOXA_48	R	R	R	R	R	R	R	R	R	R	-	R	R
3679	ERR7221282	47	31/01/2017	IRE533	188	*K. pneumoniae*	Rectal	28	blaOXA_48	R	R	S	S	S	R	S	S	R	S	S	S	S
3592	ERR7221250	45	22/02/2017	IRE558	219	*K. pneumoniae*	Rectal	1	blaOXA_48	R	R	R	R	R	R	S	I	R	S	S	S	S
3677	ERR7221270	10	06/12/2016	IRE473	922	*K. pneumoniae*	Urine	28	blaOXA_48	R	R	S	I	I	R	S	S	R	S	S	S	S
3602	ERR1829921	10	02/08/2016	IRE301	922	*K. pneumoniae*	Faeces	1	blaOXA_48	R	R	S	S	S	S	S	S	R	S	S	S	R
3577	ERR7221188	32	22/02/2017	IRE559	719	*K. pneumoniae*	Rectal	1	blaOXA_48	R	R	S	I	I	R	S	I	R	S	S	S	S
3708	ERR7221256	11	23/11/2015	IRE183	307	*K. pneumoniae*	Faeces	28	blaNDM_1	R	R	R	R	R	R	R	S	R	R	-	R	R
3687	ERR1815293	7	22/03/2016	IRE239	37	*K. pneumoniae*	Sputum	28	blaOXA_48	R	R	R	R	R	R	S	S	R	R	S	R	R
3512	ERR7221224	1	06/05/2014	IRE21	258	*K. pneumoniae*	Rectal	27	blaKPC_2	R	R	R	R	R	R	R	R	R	S	-	R	S
3580	ERR7221365	35	14/11/2016	IRE441	465	*K. pneumoniae*	Faeces	1	blaOXA_48	R	R	S	S	I	R	S	S	R	S	S	S	S
3702	ERR7221241	2	11/08/2014	IRE44	111	*K. pneumoniae*	Rectal	27	blaNDM_1	R	R	R	R	R	R	R	R	R	R	-	R	S
3554	ERR1830478	9	30/03/2016	IRE246	36	*K. pneumoniae*	Rectal	10	blaNDM_1	R	R	R	R	R	R	R	S	R	S	S	S	R
3685	ERR1813605	4	11/03/2016	IRE225	20	*K. pneumoniae*	Rectal	28	blaOXA_48	R	R	R	R	R	R	S	S	R	S	S	S	R
3656	ERR2109180	5	11/01/2017	IRE480	14	*K. pneumoniae*	Blood	26	blaOXA_48	R	R	R	R	R	-	R	S	R	S	S	S	S
3660	ERR7221165	12	01/09/2016	IRE316	23	*K. pneumoniae*	Rectal	28	blaOXA_48	R	R	R	R	I	R	S	I	R	R	R	R	R
3520	ERR7221249	44	29/08/2014	IRE51	412	*K. pneumoniae*	Urine	27	blaKPC_2	R	R	R	R	R	R	R	R	R	S	-	R	S
3515	ERR7221231	1	01/07/2014	IRE32	512	*K. pneumoniae*	Rectal	27	blaKPC_3	R	R	R	R	R	R	R	R	R	S	-	S	S
3472	ERR7221320	1	05/03/2015	IRE84	258	*K. pneumoniae*	Sputum	12	blaKPC_2	R	R	R	R	R	R	R	R	R	S	-	S	R
3565	ERR7221160	11	19/11/2015	IRE182	307	*K. pneumoniae*	Faeces	24	blaNDM_1	R	R	R	R	R	R	R	S	R	R	-	R	R
3498	ERR7221298	1	03/01/2013	IRE685	258	*K. pneumoniae*	Sputum	27	blaKPC_2	R	R	R	R	R	R	R	R	R	S	-	R	R
3636	ERR2118035	1	16/01/2017	IRE506	11	*K. pneumoniae*	Urine	17	blaOXA_48	R	R	R	R	R	-	R	I	R	R	S	R	S
3627	ERR7221284	1	15/02/2017	IRE552	11	*K. pneumoniae*	Rectal	11	blaOXA_48	R	R	R	R	R	R	R	I	R	R	R	R	S

3476	ERR1811831	6	05/02/2016	IRE212	323	*K. pneumoniae*	Urine	13	blaKPC_2	R	R	R	R	R	R	R	I	R	R	S	R	R
3475	ERR1811831	6	25/01/2016	IRE205	323	*K. pneumoniae*	Urine	13	blaKPC_2	R	R	R	R	R	R	R	R	R	R	S	R	R
3649	ERR7221183	18	14/12/2016	IRE480	45	*K. pneumoniae*	Rectal	26	blaOXA_48	R	R	S	S	S	R	S	S	R	S	S	S	S
3504	ERR7221323	1	29/04/2015	IRE98	258	*K. pneumoniae*	Rectal	27	blaKPC_2	R	R	R	R	R	R	R	R	R	S	-	S	R
3521	ERR7221251	14	21/03/2017	IRE583	29	*K. pneumoniae*	Urine	27	blaKPC_2	R	R	R	R	R	R	R	R	R	S	S	R	R
3493	ERR7221155	20	18/08/2015	IRE144	27	*K. pneumoniae*	Rectal	27	blaKPC_2	R	R	R	R	R	R	R	I	R	S	-	S	S
3514	ERR7221230	1	13/01/2014	IRE3	258	*K. pneumoniae*	Rectal	27	blaKPC_2	R	R	R	R	R	R	R	R	R	I	-	R	R
3529	ERR1815069	1	08/07/2016	IRE286	258	*K. pneumoniae*	SNS	27	blaKPC_3	R	R	R	R	R	R	R	R	R	S	R	S	R
3563	ERR7221292	27	13/10/2014	IRE64	3518	*K. pneumoniae*	Abdomi	23	blaNDM_1	R	R	R	R	R	R	R	R	R	R	-	R	R
3613	ERR7221308	3	08/09/2013	IRE702	133	*K. pneumoniae*	BAL	3	blaOXA_48	R	R	R	R	R	R	S	I	R	R	-	R	R
3684	ERR1813603	4	07/03/2016	IRE222	20	*K. pneumoniae*	Rectal	28	blaOXA_48	R	R	R	R	R	R	S	S	R	S	S	S	R
3688	ERR1829917	7	03/05/2016	IRE262	37	*K. pneumoniae*	Rectal	28	blaOXA_48	R	R	R	R	R	R	R	R	R	R	S	R	R
3696	ERR7221209	2	17/08/2015	IRE140	111	*K. pneumoniae*	Urine	27	blaNDM_1	R	R	R	R	R	R	R	R	R	R	-	R	R
3597	ERR7221271	9	09/12/2016	IRE481	834	*K. pneumoniae*	Faeces	1	blaOXA_48	R	R	R	R	R	R	S	S	R	S	S	S	S
3662	ERR7221187	14	31/01/2017	IRE534	29	*K. pneumoniae*	Rectal	28	blaOXA_48	R	R	R	R	R	R	S	I	R	S	S	S	S
3681	ERR7221286	7	14/03/2017	IRE577	37	*K. pneumoniae*	Rectal	28	blaOXA_48	R	R	R	R	R	R	R	S	R	S	S	R	S
3532	ERR1830481	10	05/04/2016	IRE251	922	*K. pneumoniae*	Urine	27	blaKPC_2	R	R	R	R	R	R	R	I	R	S	S	S	R
3540	ERR2130428	13	24/11/2016	IRE459	48	*K. pneumoniae*	Rectal	28	blaNDM_5	R	R	R	R	R	R	R	R	R	S	S	S	R
3542	ERR1815292	49	11/04/2016	IRE252	883	*K. pneumoniae*	Rectal	3	blaNDM_1	R	R	R	R	R	R	R	I	R	I	S	R	R
3634	ERR1813609	1	16/03/2016	IRE234	11	*K. pneumoniae*	Rectal	17	blaOXA_48	R	R	R	R	R	R	S	I	R	R	S	R	R
3690	ERR1830483	51	12/04/2016	IRE223	610	*K. pneumoniae*	Rectal	28	blaOXA_48	R	R	S	S	I	R	S	R	R	R	S	R	R
3703	ERR7221266	2	11/08/2014	IRE45	111	*K. pneumoniae*	Rectal	27	blaNDM_1	R	R	R	R	R	R	R	R	R	R	-	R	R
3539	ERR2118028	13	30/11/2016	IRE466	48	*K. pneumoniae*	Rectal	28	blaNDM_5	R	R	R	R	R	-	R	R	R	S	S	S	S
3572	ERR7221178	-	07/11/2016	IRE431	3516	*K. pneumoniae*	Faeces	1	blaOXA_48	R	R	S	S	I	R	S	S	R	I	I	S	S
3635	ERR2118034	1	16/01/2017	IRE505	11	*K. pneumoniae*	Urine	17	blaOXA_48	R	R	R	R	R	-	R	R	R	R	S	R	S
3698	ERR7221227	2	24/06/2014	IRE26	111	*K. pneumoniae*	Rectal	27	blaNDM_1	R	R	R	R	R	R	R	R	R	R	-	R	R
3535	ERR2109183	1	24/01/2017	IRE18	512	*K. pneumoniae*	Urine	27	blaKPC_3	R	R	R	R	R	-	R	R	R	S	R	R	S
3553	ERR1830477	2	30/03/2016	IRE245	111	*K. pneumoniae*	Rectal	10	blaNDM_1	R	R	R	R	R	R	R	I	R	S	S	S	S
3626	ERR7221181	31	23/11/2016	IRE457	1662	*K. pneumoniae*	Wound	11	blaOXA_48	R	R	S	I	R	R	S	S	R	S	S	S	S
3478	ERR1811831	6	24/10/2016	IRE397	323	*K. pneumoniae*	Rectal	13	blaKPC_2	R	R	R	R	R	-	R	R	R	R	S	R	S
3581	ERR7221306	3	31/05/2013	IRE695	133	*K. pneumoniae*	Urine	1	blaOXA_48	R	R	R	R	R	R	S	S	R	R	-	R	R
3600	ERR1810608	21	06/01/2016	IRE161	37	*K. pneumoniae*	Urine	1	blaOXA_48	R	R	S	S	S	R	S	I	R	S	S	S	R
3533	ERR1830482	8	14/04/2016	IRE254	26	*K. pneumoniae*	Rectal	27	blaKPC_2	R	R	R	R	R	R	S	I	R	S	S	S	S
3591	ERR7221237	43	26/10/2016	IRE417	556	*K. pneumoniae*	Faeces	1	blaOXA_48	R	R	S	I	R	R	S	R	R	S	S	S	S
3499	ERR7221300	1	19/04/2013	IRE689	258	*K. pneumoniae*	Sputum	27	blaKPC_2	R	R	R	R	R	R	R	R	R	S	-	R	R
3569	ERR7221162	3	13/05/2014	IRE22	133	*K. pneumoniae*	Faeces	1	blaOXA_48	R	R	R	R	R	R	R	S	R	R	-	R	R
3629	ERR7221166	16	05/09/2016	IRE326	13	*K. pneumoniae*	Urine	12	blaOXA_48	R	R	R	R	R	R	R	S	R	R	S	R	R
3482	ERR7221303	10	16/05/2013	IRE692	922	*K. pneumoniae*	Rectal	18	blaKPC_2	R	R	R	R	R	R	R	R	R	S	-	R	R
3608	ERR1840998	28	04/10/2016	IRE373	25	*K. pneumoniae*	Faeces	1	blaOXA_48	R	R	S	S	S	R	S	R	R	R	S	R	R
3650	ERR7221217	18	28/09/2015	IRE165	45	*K. pneumoniae*	Ears	26	blaOXA_48	R	R	S	S	I	R	S	S	R	S	S	S	-
3470	ERR7221197	1	06/12/2012	EM679	258	*K. pneumoniae*	Rectal	3	blaKPC_2	R	R	R	R	R	R	R	I	R	S	-	R	R
3610	ERR2118023	28	12/10/2016	IRE391	25	*K. pneumoniae*	Faeces	1	blaOXA_48	R	R	S	S	I	-	S	I	R	R	S	R	S
3637	ERR2118024	53	14/10/2016	IRE394	441	*K. pneumoniae*	Faeces	18	blaOXA_181	R	R	R	R	R	-	R	I	R	S	S	S	S
3683	ERR1811832	4	25/01/2016	IRE181	20	*K. pneumoniae*	Faeces	28	blaOXA_48	R	R	R	R	R	S	S	S	R	S	S	S	R
3615	ERR7221314	3	10/09/2013	IRE710	133	*K. pneumoniae*	Rectal	3	blaOXA_48	R	R	R	R	R	R	S	I	R	R	-	R	R
3536	ERR2118038	1	08/02/2017	IRE543	258	*K. pneumoniae*	Rectal	27	blaKPC_3	R	R	R	R	R	R	R	R	R	S	R	R	R
3547	ERR7221260	2	19/07/2016	IRE293	111	*K. pneumoniae*	Rectal	10	blaNDM_1	R	R	R	R	R	R	R	R	R	S	S	S	R
3589	ERR7221350	4	16/03/2017	IRE581	336	*K. pneumoniae*	Rectal	1	blaOXA_48	R	R	R	R	R	R	S	I	R	S	S	R	S
3501	ERR7221304	1	17/05/2013	IRE693	258	*K. pneumoniae*	Rectal	27	blaKPC_2	R	R	R	R	R	R	R	R	R	I	-	R	R
3663	ERR7221195	12	05/02/2015	IRE83	23	*K. pneumoniae*	BLC	28	blaOXA_48	R	R	S	I	I	R	S	S	R	R	-	R	R
3570	ERR7221171	17	04/10/2016	IRE377	1373	*K. pneumoniae*	Nephros	1	blaOXA_48	R	R	R	R	R	R	S	I	R	R	S	R	R
3502	ERR7221319	1	02/02/2014	IRE8	258	*K. pneumoniae*	Rectal	27	blaKPC_2	R	R	R	R	R	R	R	S	R	S	-	R	R
3709	ERR1815065	1	22/03/2016	IRE211	11	*K. pneumoniae*	Urine	26	blaNDM_19	R	R	R	R	R	R	R	R	R	R	R	R	R
3500	ERR7221301	1	24/04/2013	IRE690	258	*K. pneumoniae*	Rectal	27	blaKPC_2	R	R	R	R	R	R	R	R	R	I	-	R	R
3564	ERR2109184	11	22/11/2016	IRE182	307	*K. pneumoniae*	Rectal	23	blaNDM_1	R	R	R	R	R	-	R	I	R	R	R	R	R
3481	ERR7221198	1	30/12/2014	EM680	258	*K. pneumoniae*	Faeces	18	blaKPC_2	R	R	R	R	R	R	R	R	R	S	-	S	R
3585	ERR7221337	3	26/06/2013	IRE697	133	*K. pneumoniae*	Urine	1	blaOXA_48	R	R	R	R	R	R	S	S	R	R	-	R	R
3604	ERR1840992	10	16/09/2016	IRE340	922	*K. pneumoniae*	Faeces	1	blaOXA_48	R	R	S	S	S	S	S	S	R	S	S	S	R
3700	ERR7221229	2	24/06/2014	IRE28	111	*K. pneumoniae*	Urine	27	blaNDM_1	R	R	R	R	R	R	R	R	R	R	-	R	R
3492	ERR7221152	1	10/02/2014	IRE13	258	*K. pneumoniae*	Rectal	27	blaKPC_2	R	R	R	R	R	R	R	R	R	S	-	S	R
3713	ERR7221248	-	02/12/2016	IRE422	1308	*K. quasipneumoniae*	Rectal	1	blaOXA_48	R	R	S	R	I	R	S	R	R	S	S	S	S
3712	ERR7221238	-	03/11/2016	IRE427	1308	*K. quasipneumoniae*	Faeces	1	blaOXA_48	R	R	S	S	I	R	S	S	R	S	S	S	S
3714	ERR7221268	-	30/11/2016	IRE467	1308	*K. quasipneumoniae*	Faeces	1	blaOXA_48	R	R	R	S	R	R	S	S	R	S	S	S	S
3717	ERR1840989	-	23/08/2016	IRE309	1308	*K. quasipneumoniae*	Sputum	1	blaOXA_48	R	R	S	S	S	R	S	S	R	S	S	S	S
3718	ERR1840991	-	01/09/2016	IRE320	1308	*K. quasipneumoniae*	Urine	1	blaOXA_48	R	R	S	S	S	R	S	S	R	S	S	S	R
3721	ERR7221207	-	23/07/2015	IRE131	1859	*K. quasipneumoniae*	Wound	20	blaOXA_181	R	R	R	R	R	R	R	S	R	R	-	R	R
3716	ERR1829919	-	25/07/2016	IRE296	1308	*K. quasipneumoniae*	Faeces	1	blaOXA_48	R	R	S	S	R	R	R	S	R	S	S	S	S
3719	ERR1840994	-	21/09/2016	IRE352	1308	*K. quasipneumoniae*	Bile	1	blaOXA_48	R	R	S	S	S	R	S	R	R	S	S	S	S
3711	ERR7221184	-	23/12/2016	IRE490	1308	*K. quasipneumoniae*	Rectal	1	blaOXA_48	R	R	S	S	R	R	S	I	R	S	S	S	S
3715	ERR7221272	-	13/12/2016	IRE483	1308	*K. quasipneumoniae*	Urine	1	blaOXA_48	R	R	S	S	I	R	S	S	R	S	S	S	S
3722	ERR7221174	-	05/10/2016	IRE381	1308	*K. quasipneumoniae*	Rectal	22	blaOXA_48	R	R	S	I	I	R	S	S	R	S	S	S	S
3720	ERR2109179	-	25/10/2016	IRE415	1308	*K. quasipneumoniae*	Faeces	1	blaOXA_48	R	R	S	I	I	-	S	S	S	S	S	S	S
3710	ERR7221164	-	28/07/2016	IRE298	1308	*K. quasipneumoniae*	Faeces	1	blaOXA_48	R	R	S	S	I	R	S	S	R	S	S	S	S

3726	ERR7221408	-	11/07/2016	IRE287	227	*K. sp. M5al*	Urine	26	blaOXA_48	R	R	R	R	R	R	S	S	R	S	S	S	S
3724	ERR7221398	-	29/03/2016	IRE242	186	*K. sp. M5al*	Sputum	18	blaNDM_1	R	R	R	R	R	R	R	I	R	R	R	R	S
3725	ERR7221356	-	29/06/2016	IRE187	186	*K. sp. M5al*	Blood	12	blaOXA_48	R	R	R	R	R	R	S	S	R	R	S	R	R
3732	ERR7221173	-	06/10/2016	IRE383	1478	*K. variicola*	Sputum	25	blaOXA_48	R	R	R	I	I	R	S	S	R	S	S	S	S
3731	ERR7221199	-	27/05/2015	IRE107	1563	*K. variicola*	Urine	2	blaOXA_48	R	R	S	S	I	R	R	S	R	S	-	S	R
3730	ERR2109181	-	18/10/2016	IRE385	2978	*K. variicola*	Faeces	1	blaOXA_48	R	R	R	I	R	-	R	I	R	S	S	S	S
3734	ERR7221435	-	23/03/2017	IRE587	355	*K. variicola*	Urine	28	blaOXA_48	R	R	R	R	R	R	R	R	R	S	S	S	S
3729	ERR2109158	-	18/10/2016	IRE409	2978	*K. variicola*	Faeces	1	blaOXA_48	R	R	S	I	R	-	S	I	R	S	S	S	S
3733	ERR7221170	-	27/09/2016	IRE366	697	*K. variicola*	Rectal	26	blaOXA_48	R	R	S	I	I	R	S	R	R	S	S	S	S
3728	ERR1840988	-	09/08/2016	IRE305	919	*K. variicola*	Faeces	1	blaOXA_48	R	R	S	R	I	R	S	I	R	S	S	S	R
3727	ERR7221363	-	28/10/2016	IRE421	360	*K. variicola*	Faeces	1	blaOXA_48	R	R	S	S	S	R	S	S	R	S	S	S	S
3735	ERS8120866	-	24/05/2017	IRE640	NA	*L. decarboxylata*	Rectal	1	blaOXA_48	R	R	-	-	S	R	I	S	R	S	S	S	-
3736	ERS8120870	-	13/04/2015	IRE90	NA	*P. astuartii*	Sputum	23	blaNDM_1	R	R	R	R	R	R	R	R	R	R	-	R	R
3739	ERS8120868	-	18/08/2015	IRE147	NA	*R. ornithinolytica*	Rectal	12	blaOXA_48	R	R	R	R	R	R	R	R	R	S	-	S	R
3738	ERS8940896	-	03/09/2015	IRE154	NA	*R. ornithinolytica*	Rectal	27	blaKPC_2	R	R	R	R	R	R	R	I	R	S	-	S	S
3737	ERS8940887	-	02/11/2016	IRE425	NA	*R. ornithinolytica*	Sputum	27	blaKPC_2	R	R	R	R	R	R	R	R	R	S	S	S	R
3741	ERS8120865	-	22/04/2015	IRE78	NA	*S. marcescens*	Sputum	27	blaKPC_2	R	R	R	R	R	R	R	R	R	S	-	S	S
3743	ERS8120867	-	14/04/2016	IRE253	NA	*S. marcescens*	Foot	16	blaOXA_48	R	R	R	R	R	R	R	I	R	S	S	S	R
3744	ERS8940891	-	06/10/2016	IRE383	NA	*S. marcescens*	Sputum	25	blaOXA_48	R	R	R	R	R	R	R	S	R	S	S	R	S
3742	ERS8940892	-	17/12/2015	IRE187	NA	*S. marcescens*	Drainag	1	blaOXA_48	R	R	R	R	R	R	R	R	R	S	-	S	R
3740	ERS8940897	-	10/07/2015	IRE126	NA	*S. marcescens*	Abdomi	12	blaKPC_2	R	R	R	R	R	R	R	I	R	R	-	S	S
3745	ERS8940899	-	01/06/2017	IRE648	NA	*S. marcescens*	Urine	28	blaOXA_48	-	-	-	-	I	R	R	S	R	S	S	S	-

Supplementary Table S8. The distribution of carbapenemase gene variants in the collection of 746 CPE isolates from 2012-2017

**Carabapenemase group**	**Carbapenemase gene variant**	**Numbet of isolates***
KPC-like	$bla_{\text{KPC-2}}$	156
	$bla_{\text{KPC-3}}$	16
OXA-like	$bla_{\text{OXA-48}}$	459
	$bla_{\text{OXA-181}}$	12
	$bla_{\text{OXA-244}}$	5
	$bla_{\text{OXA-162}}$	1
NDM-like	$bla_{\text{NDM-1}}$	53
	$bla_{\text{NDM-5}}$	16
	$bla_{\text{NDM-19}}$	1
VIM-like	$bla_{\text{VIM-1}}$	17
	$bla_{\text{VIM-4}}$	1
IMP-like	$bla_{\text{IMP-4}}$	8
	$bla_{\text{IMP-70}}$ (2)	2
	$bla_{\text{IMI-1}}$ (1)	1

* Among the total 746 isolates included in the study, one carried both $bla_{\text{NDM-1}}$ and $bla_{\text{KPC-2}}$ and two carried both $bla_{\text{OXA-48}}$ and $bla_{\text{NDM-5}}$.

1. Andrews JM. Determination of minimum inhibitory concentrations. J Antimicrob Chemother. 2001;48 Suppl 1:5-16.
2. Lees JA, Harris SR, Tonkin-Hill G, Gladstone RA, Lo SW, Weiser JN, et al. Fast and flexible bacterial genomic epidemiology with PopPUNK. Genome Res. 2019;29(2):304-16.
3. Snitkin ES, Zelazny AM, Thomas PJ, Stock F, Henderson DK, Palmore TN, et al. Tracking a hospital outbreak of carbapenem-resistant Klebsiella pneumoniae with whole-genome sequencing. Sci Transl Med. 2012;4(148):148ra16.
4. David S, Reuter S, Harris SR, Glasner C, Feltwell T, Argimon S, et al. Epidemic of carbapenem-resistant Klebsiella pneumoniae in Europe is driven by nosocomial spread. Nat Microbiol. 2019;4(11):1919-29.
5. Coll F, Raven KE, Knight GM, Blane B, Harrison EM, Leek D, et al. Definition of a genetic relatedness cutoff to exclude recent transmission of meticillin-resistant Staphylococcus aureus: a genomic epidemiology analysis. The Lancet Microbe. 2020;1(8):e328-e35.
6. Mathers AJ, Stoesser N, Sheppard AE, Pankhurst L, Giess A, Yeh AJ, et al. Klebsiella pneumoniae carbapenemase (KPC)-producing K. pneumoniae at a single institution: insights into endemicity from whole-genome sequencing. Antimicrob Agents Chemother. 2015;59(3):1656-63.
7. Ludden C, Coll F, Gouliouris T, Restif O, Blane B, Blackwell GA, et al. Defining nosocomial transmission of Escherichia coli and antimicrobial resistance genes: a genomic surveillance study. The Lancet Microbe. 2021.
8. Reeves PR, Liu B, Zhou Z, Li D, Guo D, Ren Y, et al. Rates of Mutation and Host Transmission for an Escherichia coli Clone over 3 Years. PLOS ONE. 2011;6(10):e26907.
